# Myeloid AEG-1/MTDH drives inflammation and hepatocellular dysfunction in diet-induced steatohepatitis

**DOI:** 10.1016/j.jbc.2025.111101

**Published:** 2025-12-23

**Authors:** Suchismita Raha, Saranya Chidambaranathan Reghupaty, Xufeng Qu, Rachel G. Mendoza, Devon Farrar, Ali Gawi Ermi, Eva Davis, Rebecca K. Martin, Mark A. Subler, Paul B. Fisher, Jolene J. Windle, Jinze Liu, Devanand Sarkar

**Affiliations:** 1 Department of Cellular, Molecular and Genetic Medicine, Virginia Commonwealth University, Richmond, Virginia, USA; 2 Department of Pathology, Stanford University School of Medicine, Stanford, California, USA; 3 Massey Comprehensive Cancer Center, Virginia Commonwealth University, Richmond, Virginia, USA; 4 Department of Microbiology and Immunology, Virginia Commonwealth University, Richmond, Virginia, USA; 5 VCU Institute of Molecular Medicine (VIMM), Virginia Commonwealth University, Richmond, Virginia, USA; 6 Department of Biostatistics, Virginia Commonwealth University, Richmond, Virginia, USA

**Keywords:** gene regulation, liver metabolism, transcriptomics, myeloid cell, inflammation

## Abstract

Astrocyte elevated gene-1 (AEG-1)/metadherin plays an important role in regulating lipid metabolism and inflammation in hepatocytes and promotes metabolic dysfunction–associated steatohepatitis (MASH). Myeloid cells, such as macrophages, play a key role in regulating inflammation. Here, we investigated the role of AEG-1 in myeloid cells in regulating high fat)/high sugar diet (HF/HSD)–induced MASH. Littermates of myeloid cell–specific AEG-1 knockout mice (AEG-1^ΔMAC^) and AEG-1 floxed mice (AEG-1^fl/fl^) were fed either a control diet or an HF/HSD for 20 weeks. Both male and female AEG-1^ΔMAC^ mice were significantly protected from MASH development compared with AEG-1^fl/fl^ mice. In control diet–fed mice, spatial transcriptomics analysis revealed inhibition of hepatic steatosis and development of hepatocellular carcinoma and activation of fatty acid β-oxidation in the periportal and pericentral hepatocytes of AEG-1^ΔMAC^ livers. Single-cell RNA-Seq, performed in HF/HSD-fed mice, identified a significant decrease in the total number of Kupffer cells in AEG-1^ΔMAC^*versus* AEG-1^fl/fl^ livers. A marked inhibition of inflammation and fibrosis in Kupffer cells and stellate cells, increased fatty acid β-oxidation in stellate and endothelial cells, and inhibition of proliferation and invasion in the hepatocytes, especially in pericentral hepatocytes, were observed in AEG-1^ΔMAC^ liver *versus* AEG-1^fl/fl^ liver. Mesenteric fat weight, adipocyte size, and inflammation were significantly decreased in HF/HSD-fed AEG-1^ΔMAC^ mice compared with AEG-1^fl/fl^ mice. Inhibition of inflammation is a key feature in AEG-1^ΔMAC^ mice. AEG-1 in myeloid cells regulate gene expression in hepatocytes and other nonparenchymal cells, thereby playing an important role in regulating MASH.

In the United States, one in two adults is projected to be obese by 2030 (1). A diet containing high fat and high sugar (HF/HS) is a key factor for obesity (2), which is closely associated with metabolic dysfunction–associated steatohepatitis (MASH), the most common cause of chronic liver disease in the Western world, leading to cirrhosis and hepatocellular carcinoma (HCC) (3, 4). Some molecular players regulating MASH have been identified, leading to multiple strategies in clinical trials (5). However, optimum therapy is yet to be developed, motivating a clearer understanding of the molecular mechanism of MASH to yield effective therapeutic strategies. MASH is characterized by initial accumulation of triglycerides (TGs) in hepatocytes (hepatic steatosis) with subsequent chronic inflammation (5, 6). In the liver, macrophages, such as liver-resident Kupffer cells (KCs) and infiltrating monocytes/macrophages from circulation, release proinflammatory cytokines (PICs) and play a central role in regulating the inflammatory component of MASH.

Astrocyte elevated gene-1/metadherin is an oncogene, which is overexpressed in many cancers, including HCC and promotes HCC development and progression (7). AEG-1 is a scaffold protein, which functions by protein–protein and protein–RNA interactions and also plays an important role in promoting MASH (8). Our recent work revealed that a hepatocyte-specific AEG-1 transgenic mouse (Alb/AEG-1) develops spontaneous MASH, and a hepatocyte-specific conditional AEG-1 knockout mouse (AEG-1^ΔHEP^) is protected from high-fat diet (HFD)–induced MASH (8). AEG-1 levels are markedly high in biopsy samples of human MASH livers compared with normal livers (8). Mechanistically, we documented that there are two ways AEG-1 promotes steatosis (8). Peroxisome proliferator–activated receptor alpha (PPARα) is a master regulator of genes that control fatty acid β-oxidation (FAO) (9). Using an “LXXLL” motif, AEG-1 interacts with retinoid X receptor, the heterodimeric partner of PPARα (8, 10). This interaction inhibits coactivator recruitment to PPARα and PPARα-mediated gene regulation, thereby inhibiting FAO (8, 10). As an endoplasmic reticulum membrane–anchored RNA-binding protein, AEG-1 preferentially binds to mRNAs that code for fatty acid–synthesizing enzymes and increases their translation, which leads to increased *de novo* lipogenesis (DNL) (8, 11). NF-κB is a transcription factor that regulates the expression of PICs and functions as a master regulator of inflammation (12). AEG-1 interacts with multiple components of NF-κB signaling pathway, and it is fundamentally required for the activation of NF-κB (13, 14, 15, 16). By activating NF-κB, AEG-1 promotes the inflammatory and fibrotic components of MASH (8). These studies clearly establish AEG-1 as a central player that promotes steatosis, inflammation, and fibrosis, essential components of MASH, by multiple mechanisms. A hepatocyte-targeted nanoparticle delivering AEG-1 siRNA (PAMAM-AEG-1si) effectively blocked HFD-induced MASH in C57BL/6 mice, thereby establishing the “proof of principle” that targeting AEG-1 by RNAi strategy might be effectively used for therapeutic intervention of MASH (8).

AEG-1 is highly expressed in myeloid cells, which include macrophages (17). We have documented that AEG-1 plays a vital role in regulating macrophage activation, and mice with deletion of AEG-1 in myeloid cells (AEG-1^ΔMAC^) are profoundly resistant to *N*-nitrosodiethylamine-induced inflammatory HCC (17). RNA-Seq analysis unraveled robust inhibition of upstream regulators of inflammation, immune response, and cytokine signaling in AEG-1^ΔMAC^ macrophages compared with AEG-1^fl/fl^ macrophages (17). In the present study, we interrogated the response of AEG-1^ΔMAC^ mice to HF/HSD-induced MASH development. We observed that AEG-1^ΔMAC^ mice are also resistant to HF/HSD-induced MASH, thereby unraveling an important role of myeloid cell AEG-1 in regulating MASH.

## Results

### MASH development is inhibited in AEG-1^ΔMAC^ mice

**Figure 1:**
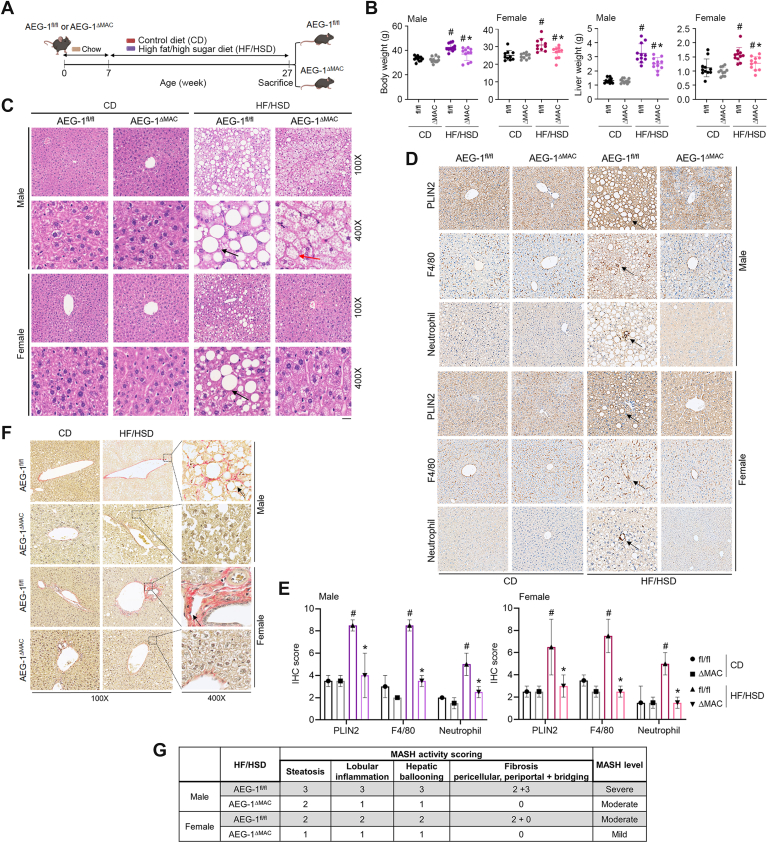
**AEG-1^ΔMAC^ mice are protected from high-fat diet/high-sugar diet (HF/HSD)-induced MASH**. *A*, study design and experimental timeline of control diet (CD)–fed and HF/HSD-fed AEG-1^fl/fl^ and AEG-1^ΔMAC^ mice. *B*, measurement of body and liver weights (n = 11 per group) in the indicated groups at the end of the experiment. Data represent mean ± SEM; #*p* < 0.05 *versus* corresponding CD-fed mice; ∗*p* < 0.05 between HF/HSD-fed AEG-1^fl/fl^ and AEG-1^ΔMAC^ mice. *C*, representative photomicrograph of H&E-stained liver sections in the indicated groups at the end of the experiment. *Arrows* indicate steatosis. The scale bar represents 20 μm. *D*, representative photomicrographs of immunohistochemical staining of the indicated proteins/markers in the liver sections in the indicated groups at the end of the experiment. Magnification: 400x. *E*, quantification of immunohistochemistry score (H score) for the indicated markers. Data represent mean ± SEM; #*p* < 0.01 *versus* corresponding CD-fed mice; ∗*p* < 0.01 between HF/HSD-fed AEG-1^fl/fl^ and AEG-1^ΔMAC^ mice. *F*, representative photomicrographs of Sirius red staining of liver sections in the indicated groups at the end of the experiment. *G*, MASH activity scoring following the Clinical Research Network (CRN) MASH Scoring System. MASH, metabolic dysfunction–associated steatohepatitis.

A detailed characterization of myeloid cell–specific conditional AEG-1 knockout mouse (AEG-1^ΔMAC^) was described in our previous publication (17). We fed 7-week-old male and female AEG-1^fl/fl^ and AEG-1^ΔMAC^ littermates (n = 11) either a control diet (CD) or an HF/HSD for 20 weeks (Fig. 1A). At the end of the experiment at 27 weeks, livers of AEG-1^fl/fl^ and AEG-1^ΔMAC^ littermates of both sexes were paler when fed HF/HSD *versus* CD (Fig. S1). At this time point, in both male and female AEG-1^fl/fl^ and AEG-1^ΔMAC^ littermates, body and liver weights on HF/HSD were significantly higher than those on CD (Fig. 1*B*). There were no significant differences in body and liver weights between AEG-1^fl/fl^ and AEG-1^ΔMAC^ littermates of both sexes on CD. However, on HF/HSD, both body and liver weights of AEG-1^ΔMAC^ mice were significantly lower than those of AEG-1^fl/fl^ littermates (Fig. 1*B*). We checked H&E-stained sections of liver tissues for steatosis following the Clinical Research Network MASH Scoring System (18). For AEG-1^fl/fl^ mice fed HF/HSD, 100% mice developed moderate (grade 2, score: 33–66%) to severe (grade 3, score: >66%) microvesicular and macrovesicular steatosis (Fig. 1*C*), whereas ∼50% female mice developed mild microvesicular and macrovesicular steatosis (grade 1, score 5–33%). However, for HF/HSD-fed AEG-1^ΔMAC^ mice, 100% mice developed mild microvesicular and macrovesicular steatosis, and only 15% female mice showed features of mild microvesicular and macrovesicular steatosis (Fig. 1*C*).

To assess the severity of MASH, we stained liver sections for steatosis marker perilipin-2 (PLIN2) and macrophage marker F4/80, and with antineutrophil antibody (Fig. 1, *D* and *E*). In HF/HSD-fed AEG-1^fl/fl^ mice, PLIN2 staining unraveled significantly high levels of microvesicular and macrovesicular steatosis and 100% large hepatocellular ballooning (HB) with strong intensity in male mice and 35% small to 25% medium HB with moderate intensity in female mice (Fig. 1, *D* and *E*). In HF/HSD-fed AEG-1^ΔMAC^ mice, 25% small to 10% medium HB with moderate intensity were detected in male mice, and 20% small HB with weak intensity was detected in female mice. These findings showed concurrence with steatosis grading in H&E-stained sections (Fig. 1*C*). Upon HF/HSD feeding, in AEG-1^fl/fl^ male mice, 30% partial to 60% complete F4/80 foci were observed, and in AEG-1^fl/fl^ female mice, 35% partial to 25% complete foci were observed, whereas in AEG-1^ΔMAC^ mice, F4/80 foci were weak and partial with 35% in male mice and 20% in female mice (Fig. 1, *D* and *E*). Antineutrophil staining showed strong intensity in male and moderate intensity in female for HF/HSD-fed AEG-1^fl/fl^ mice and moderate intensity in male and weak intensity in female for HF/HSD-fed AEG-1^ΔMAC^ mice (Fig. 1, *D* and *E*). In HF/HSD-fed AEG-1^fl/fl^ mice, Sirius red staining detected strong staining in males and moderate staining in females in pericentral (PC) and periportal (PP) zones, indicating development of fibrosis (Fig. 1*F*). While bridging fibrosis was observed in HF/HSD-fed AEG-1^fl/fl^ livers, no significant bridging fibrosis was observed in HF/HSD-fed AEG-1^ΔMAC^ mice (Fig. 1, *F* and *G*). The MASH activity, calculated according to the Clinical Research Network scoring system, showed severe MASH in male and moderate MASH in female AEG-1^fl/fl^ mice and moderate MASH in male and mild MASH in female AEG-1^ΔMAC^ mice (Fig. 1*G*). These findings indicate that the magnitude of MASH was significantly attenuated in AEG-1^ΔMAC^ mice compared with AEG-1^fl/fl^ mice.

**Figure 2:**
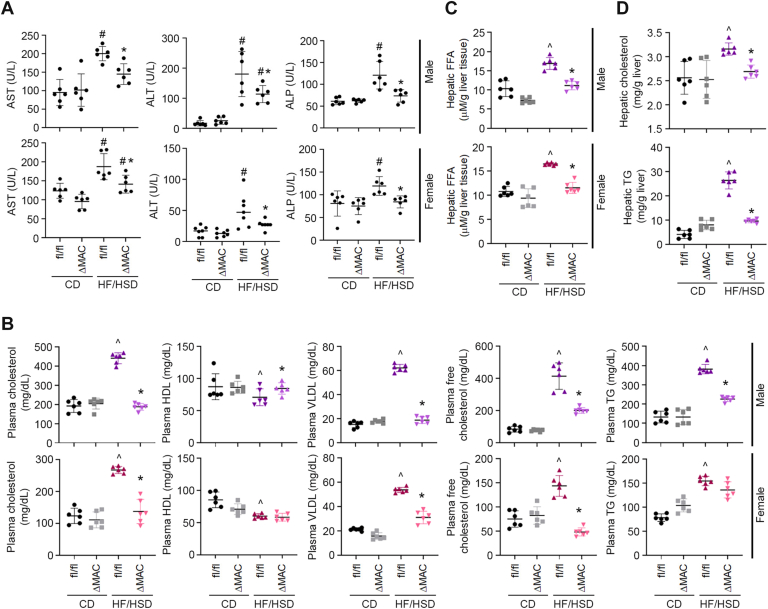
**Liver enzymes and serum and hepatic lipid profiles**. *A*, measurement of liver enzymes in mice sera at the end of the experiment. Data represent mean ± SEM; #*p* < 0.05 *versus* corresponding control diet (CD)–fed mice; ∗*p* < 0.05 between high-fat diet (HFD)/high-sugar diet (SD)–fed AEG-1^fl/fl^ and AEG-1^ΔMAC^ mice. *B*, measurement of the indicated lipids in mice sera at the end of the experiment. *C* and *D*, measurement of free fatty acid (FFA) (*C*) and cholesterol and triglyceride (TG) in the livers at the end of the experiment. For *B*–*D*, data represent mean ± SEM; ˆ*p* < 0.05 *versus* corresponding CD-fed mice; ∗*p* < 0.05 between HF/HSD-fed AEG-1^fl/fl^ and AEG-1^ΔMAC^ mice. ALP, alkaline phosphatase; ALT, alanine aminotransferase; AST, aspartate aminotransferase; HDL, high-density lipoprotein; VLDL, very low-density lipoprotein.

In both male and female AEG-1^fl/fl^ mice, a significant increase in liver enzymes, aspartate aminotransferase, alanine aminotransferase, and alkaline phosphatase, was observed with HF/HSD compared with CD (Fig. 2*A*). In male AEG-1^ΔMAC^ mice, a significant increase was observed only for alanine aminotransferase when fed HF/HSD compared with CD, and in female HF/HSD-fed AEG-1^ΔMAC^ mice, a significant increase was observed only for aspartate aminotransferase compared with CD (Fig. 2*A*). For all three liver enzymes and for both sexes, the levels were significantly lower in HF/HSD-fed AEG-1^ΔMAC^ mice *versus* HF/HSD-fed AEG-1^fl/fl^ littermates (Fig. 2*A*). Upon HF/HSD feeding, an elevation of plasma total cholesterol (TC), very low-density lipoprotein (VLDL), free cholesterol (FC), and total TG levels, and a decrease in plasma high-density lipoprotein (HDL) levels were observed in AEG-1^fl/fl^ mice of both sexes (Fig. 2*B*). On the contrary, both male and female AEG-1^ΔMAC^ mice showed a significant decrease in plasma TC, VLDL, and FC levels (Fig. 2*B*). With HF/HSD feeding, plasma HDL level showed a significant increase, and plasma TG levels showed a significant decrease only in male AEG-1^ΔMAC^ mice but not in female mice (Fig. 2*B*). Free fatty acid (FFA) plays a crucial role in steatosis development. In both male and female HF/HSD-fed AEG-1^fl/fl^ mice, hepatic FFA levels were significantly higher compared with CD, and these levels were significantly lower in HF/HSD-fed AEG-1^ΔMAC^ mice (Fig. 2*C*). In male HF/HSD-fed AEG-1^ΔMAC^ mice, hepatic cholesterol and TG levels were significantly lower than those in male HF/HSD-fed AEG-1^fl/fl^ littermates (Fig. 2*D*). These findings further document that AEG-1^ΔMAC^ mice were protected from HF/HSD-induced MASH compared with AEG-1^fl/fl^ mice.

### Spatial transcriptomics analysis of CD-fed AEG-1^fl/fl^ and AEG-1^ΔMAC^ littermates

**Figure 3:**
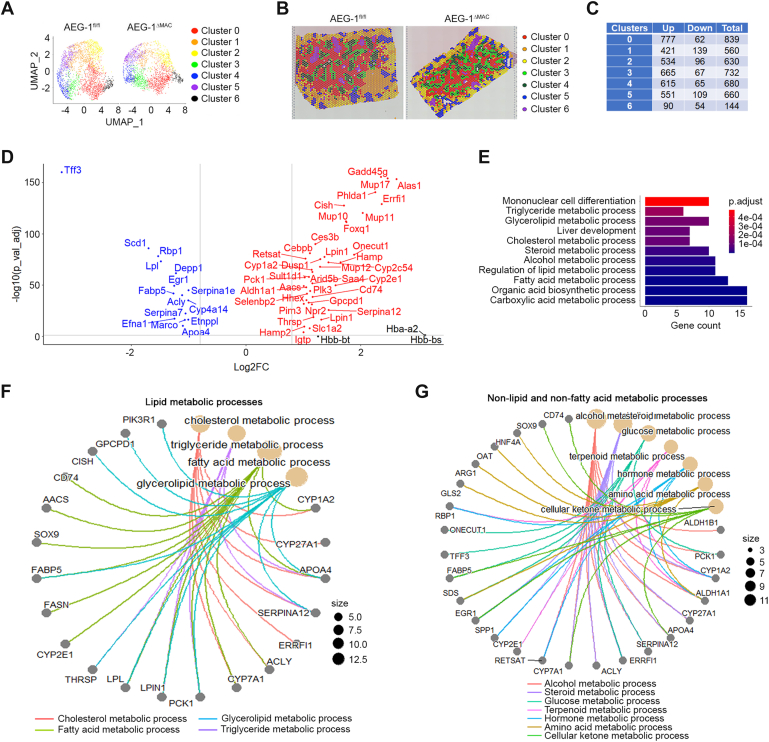
**Spatial transcriptomics (ST) analysis shows inhibition of metabolic processes in control diet (CD)–fed AEG-1^ΔMAC^ liver**. *A*, uniform manifold approximation and projection (UMAP) plots of seven clusters in AEG-1^fl/fl^ and AEG-1^ΔMAC^ livers. *B*, distribution of seven different clusters in AEG-1^fl/fl^ and AEG-1^ΔMAC^ livers identified by ST analysis. *C*, number of differentially expressed genes (DEGs) in each cluster of AEG-1^ΔMAC^ liver. Up, upregulated DEGs; Down, downregulated DEGs; Total, total number of upregulated and downregulated DEGs. *D*, volcano plot of DEGs with >+1 or <-1 log2 fold change (log2FC) in CD-fed AEG-1^ΔMAC^ mice. *E*, Gene Ontology (GO) enrichment analysis of DEGs shown in (*D*) in CD-fed AEG-1^ΔMAC^ mice. *F* and *G*, GO cnetplot showing interactions of DEGs shown in (*D*) with functional networks, such as lipid metabolic processes (*F*) and nonlipid and non–fatty acid metabolic processes (*G*) in CD-fed AEG-1^ΔMAC^ mice.

Hepatocytes in PP (zone 1), midlobular (ML, zone 2), and PC (PC, zone 3) zones have distinct metabolic functions regulating MASH (19). To obtain insights into the effects of the deletion of AEG-1 in myeloid cells on hepatocytes of different zones, we performed spatial transcriptomics (ST) on liver sections of CD-fed AEG-1^fl/fl^ and AEG-1^ΔMAC^ littermates. H&E-stained slides of liver sections are shown in Figure S2. For each sample, spots with counts greater than 50,000 and feature numbers less than 100 or greater than 7000 were removed. After this quality control, for AEG-1^fl/fl^ and AEG-1^ΔMAC^ livers, the number of spots was 2543 and 2149, respectively, and the median genes per spot were 3641 and 4096, respectively, indicating consistency between the two samples. In each sample, seven distinct clusters of spatial spots were identified, each characterized by unique gene expression profiles (Fig. 3, *A* and *B*). A list of highly expressed genes to annotate each cluster is presented in Table S1. There was no difference in cell numbers in each cluster between the two groups (data not shown). We focused our analysis on differentially expressed genes (DEGs) in each cluster between AEG-1^fl/fl^ and AEG-1^ΔMAC^ livers, using a cutoff of adjusted *p* value (p-adj) of 0.05 (Fig. 3*C*). Clusters 0 to 5 showed similar patterns of DEGs, whereas cluster 6 showed the least number of DEGs (Fig. 3*C*). DEGs in all the clusters with a log2 fold change of >+1 or <-1 and a p-adj of 0.05 are shown in a volcano plot (Fig. 3*D* and Table S2). Gene Ontology (GO) enrichment analysis of these DEGs revealed several metabolic processes, including TG, cholesterol, and fatty acid metabolic processes, mononuclear cell differentiation, and liver development to be modulated in AEG-1^ΔMAC^ livers *versus* AEG-1^fl/fl^ livers (Fig. 3*E*). To visualize the gene–pathway relationships of DEGs enriched with common biological functions, we prepared cnetplot using GO chord package of R program, showing genes associated with lipid metabolic processes (Fig. 3*F*), nonlipid and nonfatty acid metabolic processes (Fig. 3*G*), as well as biosynthesis processes, homeostasis, and cellular proliferation and differentiation (Fig. S3). A few important genes regulating lipid metabolism include ATP citrate lyase, the primary enzyme responsible for the synthesis of cytosolic acetyl-CoA, thereby contributing to lipogenesis and cholesterogenesis; lipoprotein lipase, fatty acid synthase (FASN), and fatty acid binding protein 5, which were all downregulated in AEG-1^ΔMAC^ livers (Fig. 3*F*). These analyses unraveled key genes modulated in AEG-1^ΔMAC^ livers when compared with AEG-1^fl/fl^ livers that contributed to the less aggressive MASH phenotype observed in AEG-1^ΔMAC^ mice.

We utilized the gene expression data generated by the ST analysis to determine which areas of the mouse liver samples represent each zone. Markers for zone 1 (PP) hepatocytes (*Pck1*, *Hal*, *Gls2*, *Cyp2f2*, *Hsd17b13*, and *Arg1*), zone 2 (ML) hepatocytes (*Hamp*, *Hamp2*, and *Igfbp2*), and zone 3 (PC) hepatocytes (*Cyp2e1*, *Cyp1a2*, and *Oat*) are known to sufficiently characterize hepatic zonation and thus were employed in our targeted clustering analysis (20). The spatial spots were reclustered using the gene expression profiles of the 12 zone-specific markers. This allowed us to identify the three established liver zones, each of which is enriched with distinct markers, as shown in the violin plots of zone-specific markers (Fig. S4*A*). The hepatocytes in each zone were similarly distributed between AEG-1^fl/fl^ and AEG-1^ΔMAC^ livers without showing significant differences in their numbers and any overlap (Fig. S4, *B*–*C*). These distinct patterns of expression within the PP, ML, and PC hepatocytes provided a visual representation of where the zones are localized in our mouse tissue samples.

After examining liver zonation, we subsequently aimed to localize principal nonparenchymal cell types. Cell type–specific markers for KCs (*Marco*, *Clec4f*, *Fabp5*, *Cd5l*, and *Vsig4*), cholangiocytes (*Spp1* and *Epcam*), and hepatic stellate cells (HSCs; *Reln*, *Dcn*, *Colec11*, and *Ecm1*) were utilized to determine areas of enriched spots. Violin plot of these cell type–specific markers demonstrated their expression in each specific cell type (Fig. S5*A*). The nonparenchymal cells were similarly distributed between AEG-1^fl/fl^ and AEG-1^ΔMAC^ livers without showing significant differences in their numbers (Fig. S5*B*). We observed nearly exclusive expression patterns of each cell type’s markers in AEG-1^fl/fl^ and AEG-1^ΔMAC^ livers (Fig. S5*C*).

**Figure 4:**
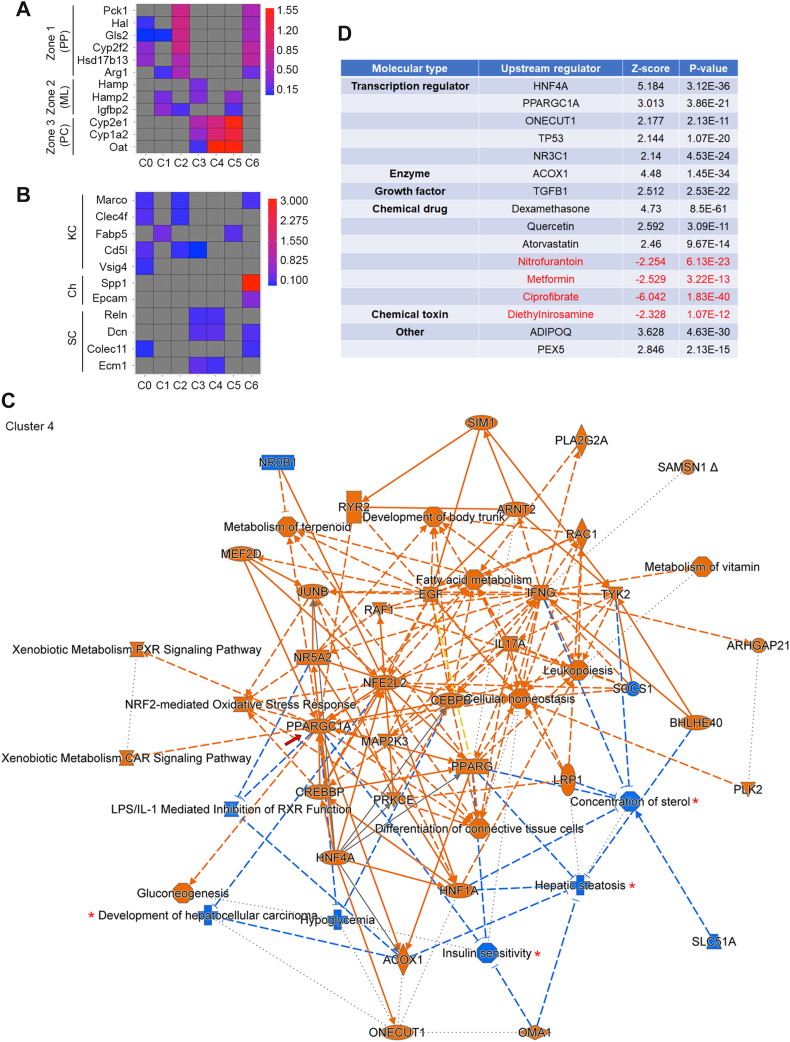
**Spatial transcriptomics (ST) analysis shows modulation of genes and pathways in pericentral hepatocytes in control diet (CD)–fed AEG-1^ΔMAC^ mice**. *A* and *B*, heatmaps showing markers of different zones (*A*) and different nonparenchymal cell types (*B*) in seven different clusters. *C*, graphical summary showing activation or inhibition of key regulatory molecules, pathways, functions, and phenotypes in cluster 4, representing pericentral hepatocytes, of AEG-1^ΔMAC^ liver compared with AEG-1^fl/fl^ liver. *Orange color* indicates activation of *z*-score >2, whereas *blue color* indicates inhibition of *z*-score <2. The shapes indicate the following: *octagon*: function; *cross*: disease; *inverted triangle*: kinase; *ellipse*: transcription regulator; *square*: cytokine; *trapezoid*: transporter; *circle*: molecule of other class; and *hourglass*: canonical pathway. *D*, upstream regulators of differentially expressed genes (DEGs) of cluster 4 of AEG-1^ΔMAC^ liver (*z*-score >2 indicates activation, and *z*-score of <-2 indicates inhibition).

The expression level of zone-specific marker genes in each cluster is shown in Figure 4*A*, whereas the expression level of cell type–specific marker genes in each cluster is shown in Figure 4*B*. Based on the expression of zone- and cell type–specific markers, the seven clusters could be classified as follows: cluster 0: PP (zone 1) with KCs; cluster 1: ML (zone 2); cluster 2: PP (zone 1) with KCs; cluster 3: PC (zone 3) close to ML (zone 2) with stellate cells; cluster 4: PC (zone 3) with stellate cells; cluster 5, PC (zone 3) close to ML (zone 2); and cluster 6: PP (zone 1) with cholangiocytes (Fig. 4, *A* and *B*). The DEGs in each cluster were subjected to ingenuity pathway analysis (IPA), which showed widespread and significant changes in AEG-1^ΔMAC^ livers *versus* AEG-1^fl/fl^ livers (Figs. 4*C*, S6, S7, S8). PP and PC hepatocytes (clusters 0, 2, 3, 4, and 5 exhibited similar features, notably inhibition of hepatic steatosis, concentration of lipids, hypoglycemia, and development of HCC, with an increase in oxidation of lipids, which are relevant to the present studies (*asterisks* in Figs. 4*C*, S6, S7, S8). One key molecule that was activated in all these clusters is peroxisome proliferative–activated receptor, gamma, coactivator 1 alpha (PPARGC1A), a key transcription coactivator of PPARs (*arrow* in Figs. 4*C*, S6, S7, S8). PPARA is a master regulator of FAO (9), and activation of PPARGC1A suggests increased FAO in AEG-1^ΔMAC^ hepatocytes. However, in cluster 1, representing ML (zone 2) hepatocytes, fatty acid metabolism was less affected (Fig. S6). Rather, there was inhibition of sterol regulatory element binding transcription factor 2, which regulates cholesterol metabolism (Fig. S6). Carbohydrate metabolism was modulated in all the clusters, including cluster 6, which showed the least number of DEGs with modulation of a few pathways (Figs. 4*C*, S6, S7, S8). Analysis of upstream regulators of these DEGS (Fig. 4*D* showing upstream regulators of DEGs of cluster 4, unraveled activation of several tumor suppressors, such as hepatocyte nuclear factor 4A, TP53, and transforming growth factor β1 (TGFB1), and inhibition of the hepatocarcinogen diethylnitrosamine in AEG-1^ΔMAC^ hepatocytes. There was activation of anti-inflammatory molecules, such as nuclear receptor subfamily 3 group C member 1, encoding glucocorticoid receptor, and its ligand dexamethasone, which correlate with the lack of inflammation observed in AEG-1^ΔMAC^ livers (Fig. 4*D* and 1D-E). Acyl-CoA oxidase 1 (ACOX1), the first enzyme in FAO, ciprofibrate, an activator of PPARA, and atorvastatin, which inhibits cholesterol synthesis, were activated in AEG-1^ΔMAC^ livers (Fig. 4*D*). These findings indicate that deletion of AEG-1 from myeloid cells affects basal gene expression in hepatocytes in a zone-specific manner. In addition, these changes in hepatocyte gene expression reflect a basal state, which is inhibitory to the development of MASH and HCC in AEG-1^ΔMAC^ mice.

### Single-cell RNA-Seq profiling of HF/HSD-fed AEG-1^fl/fl^ and AEG-1^ΔMAC^ littermates

**Figure 5:**
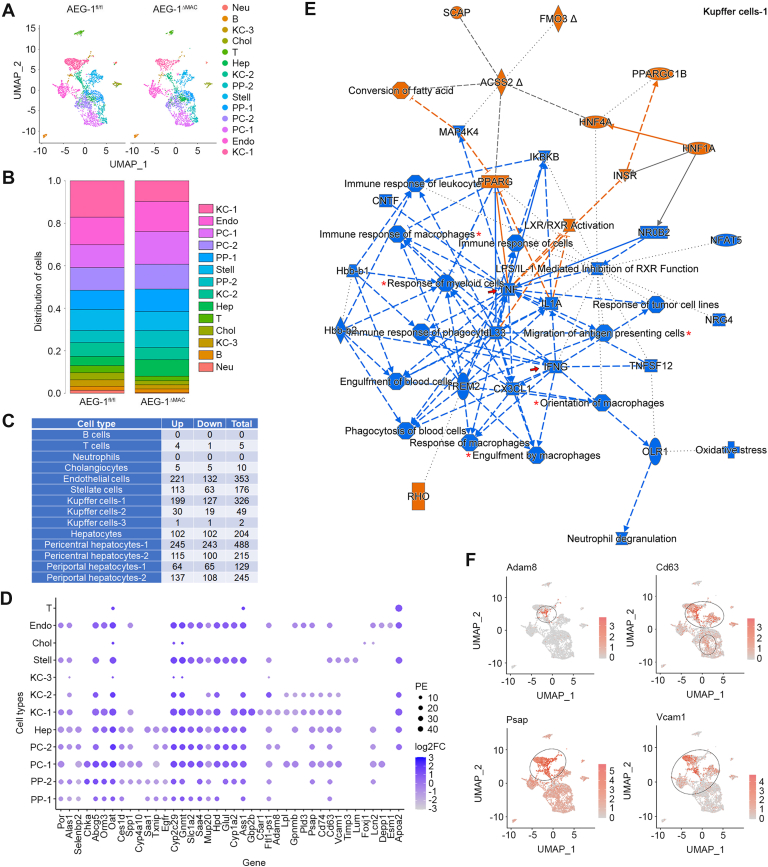
**Single-cell RNA-Seq (scRNA-Seq) identifies inhibition of Kupffer cells (KCs) in high-fat diet (HFD)/high-sugar diet (HSD)–fed AEG-1^ΔMAC^ mice**. *A*, uniform manifold approximation and projection (UMAP) plots showing 14 different cell type–specific clusters. *B*, distribution of cells in each cell type cluster. For *A* and *B*, B, B cells; Chol, cholangiocytes; Endo, endothelial cells; Hep, hepatocytes; KC-1, Kupffer cells-1; KC-2, Kupffer cells-2; KC-3, Kupffer cells-3; Neu, neutrophils; PC-1, pericentral hepatocytes-1; PC-2, pericentral hepatocytes-2; PP-1, periportal hepatocytes-1; PP-2, periportal hepatocytes-2; Stell, stellate cells; and T, T cells. *C*, number of differentially expressed genes (DEGs) in each cell type cluster of AEG-1^ΔMAC^ liver. Up, upregulated DEGs; Down, downregulated DEGs; Total, total number of upregulated and downregulated DEGs. *D*, dot plot showing expression of DEGs >+1 or <-1 _log2FC in each cell type cluster. The size of the dot indicates the percentage of expression (PE) of genes with respect to the proportion of cells within each cell type cluster. *E*, graphical summary showing activation or inhibition of key regulatory molecules, pathways, functions, and phenotypes in KC-1 of HF/HSD-fed AEG-1^ΔMAC^ liver compared with AEG-1^fl/fl^ liver. The shapes and colors are as indicated in Figure 4*C*. *F*, UMAP showing expression of the indicated genes in HF/HSD-fed AEG-1^fl/fl^ liver identified by scRNA-Seq. The *circles* show KC populations.

We next analyzed the response of AEG-1-deleted KCs, and consequently of hepatocytes and other nonparenchymal cells, to HF/HSD feeding by performing single-cell RNA-Seq (scRNA-Seq) in livers from HF/HSD-fed AEG-1^fl/fl^ and AEG-1^ΔMAC^ littermates. In AEG-1^fl/fl^ liver, there were 3400 cells representing 14,736 genes, and in AEG-1^ΔMAC^ liver, there were 2524 cells representing 14,360 genes. Fourteen clusters were identified representing different cell types (Fig. 5*A*). Markers that were used in ST to identify cell types (Fig. 4, *A* and *B*) were also used for scRNA-Seq. Additional markers used were as follows: endothelial cells: Ushbp1, Oit3, Il1a, Mmrn2, Pcdh12, and Dpp4; T cells: Trbc1, Trbc2, Cd3g, and Cd3d; B cells: Iglc2, Ighm, Igkc, Ms4a1, Cd79a, and Vpreb3; neutrophils: Cxcr2, Mmp9, Csf3r, and Srgn; and NK cells: Ncr1, Klre1, Klre1, and Nkg7. Based on gene expression profiles, we identified three clusters of KCs, 2 clusters of PP hepatocytes, 2 clusters of PC hepatocytes, and one cluster each of other hepatocytes, cholangiocytes, stellate cells, endothelial cells, neutrophils, B cells, and T cells (Fig. 5*A*). A significant decrease in the number of KC-1 and a significant increase in PC-2 were observed in AEG-1^ΔMAC^ livers *versus* AEG-1^fl/fl^ (Fig. 5*B*). No significant difference was observed for other cell types between these two groups. The numbers of DEGs in each cell type are shown in Figure 5*C*. Deletion of AEG-1 from myeloid cells had little effect on gene expression of B and T cells, neutrophils, and cholangiocytes, with notable changes in endothelial cells, stellate cells, and hepatocytes (both PP and PC) (Fig. 5*C*). Gene expression was affected more in PC hepatocytes *versus* PP hepatocytes, the former showing 703 DEGs, whereas the latter showing 374 DEGs (Fig. 5*C*). Among the KCs, KC-1 showed the most change in gene expression, whereas KC-3 showed almost no change (Fig. 5*C*). DEGs in all the cell types with a log2 fold change of >+1 or <-1 and a p-adj of 0.05 were plotted to visualize their distribution patterns (Fig. 5*D*). The majority of the genes were differentially expressed in many cell types, although the percent expression was variable, indicated by the size of the circles, depending upon the cell type (Fig. 5*D*).

To unravel the effects of the DEGs in each cell type, the DEGs, with a p-adj of 0.05, were subjected to IPA. Table S3 shows the canonical pathways activated or inhibited in each cell type in AEG-1^ΔMAC^ liver *versus* AEG-1^fl/fl^ liver. In the KC-1 of AEG-1^ΔMAC^ liver, significant inhibition of macrophages was evident, as indicated by inhibition of the response of myeloid cells, immune response of macrophages, migration of antigen-presenting cells, orientation of macrophages, and engulfment of macrophages (*asterisks* in Fig. 5*E*). This observation is in line with our previous finding that AEG-1 is required for macrophage function (17). Inflammatory molecules, such as tumor necrosis factor α (TNFα) and interferon-γ, were inhibited in KC-1 of AEG-1^ΔMAC^ livers (*arrows* in Fig. 5*E*). ADAM metallopeptidase domain 8 (Adam8), known to activate TNF signaling (21), was significantly overexpressed in KC-1 of AEG-1^fl/fl^ livers compared with KC-1 of AEG-1^ΔMAC^ livers (Fig. 5*F*). Molecules known to regulate macrophage function and inflammation, such as CD63, prosaposin, and vascular cell adhesion molecule 1 (22, 23, 24), were also overexpressed in KC-1 of AEG-1^fl/fl^ livers *versus* AEG-1^ΔMAC^ livers (Fig. 5*F*). These genes were upregulated in hepatocytes and other cell types of AEG-1^fl/fl^ livers. But the highest level of upregulation was observed in KC-1 (Fig. 5*F*). The decrease in the number of KC-1 and their inhibitory status correlate with decreased F4/80 staining and inflammation observed in liver sections (Fig. 1, *E* and *G*). Concordant with ST findings, activation of PPARGC1 was also observed in KC-1 of AEG-1^ΔMAC^ livers (Fig. 5*E*).

**Figure 6:**
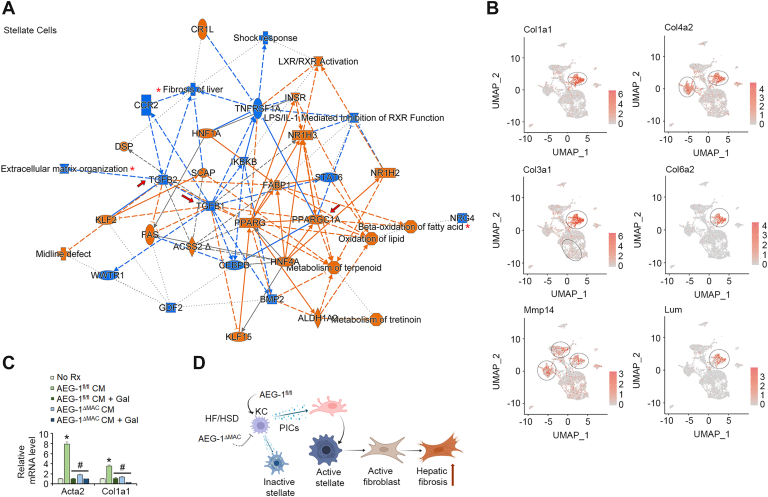
**Single-cell RNA-Seq (scRNA-Seq) identifies inhibition of hepatic fibrosis in high-fat diet (HFD)/high-sugar diet (HSD)–fed AEG-1^ΔMAC^ mice**. *A*, graphical summary showing activation or inhibition of key regulatory molecules, pathways, functions, and phenotypes in stellate cells of HF/HSD-fed AEG-1^ΔMAC^ liver compared with AEG-1^fl/fl^ liver. The shapes and colors are as indicated in Figure 4*C*. *B*, uniform manifold approximation and projection (UMAP) showing expression of the indicated genes in HF/HSD-fed AEG-1^fl/fl^ liver identified by scRNA-Seq. The *circles* show stellate cell populations. *C*, WT hepatic stellate cells (HSCs) were treated with the indicated conditioned media (CM), and expression of the indicated genes was detected by quantitative RT–PCR. Data represent mean ± SEM of triplicate experiments. Gal, galunisertib (500 nM) treatment. ∗*p* < 0.01 *versus* no Rx; #*p* < 0.01 *versus* AEG-1^fl/fl^ CM. *D*, *cartoon* showing the mechanism of increased fibrosis in HF/HSD-fed AEG-1^fl/fl^ liver. HF/HSD feeding activates KC in AEG-1^fl/fl^ liver, which releases proinflammatory cytokines (PICs). PICs activate stellate cells, which release collagens and other fibrogenic molecules, causing fibrosis. In AEG-1^ΔMAC^ liver, HF/HSD feeding does not activate KC, thereby abrogating fibrosis.

Since we observed development of fibrosis only in AEG-1^fl/fl^ livers with HF/HSD feeding (Fig. 1*F*), we focused on the response of the stellate cells. Indeed, inhibition of TGFB1 and TGFB2 and inhibition of fibrosis of liver and extracellular matrix organization were notably observed in the stellate cells of HF/HSD-fed AEG-1^ΔMAC^ livers compared with those of AEG-1^fl/fl^ littermates (*arrows* and *asterisks* in Fig. 6*A*). Interestingly, activation of PPARGC1A, and consequently FAO, was also observed in these cells (*arrow* and *asterisk* in Fig. 6*A*). Genes known to promote fibrosis, such as collagen type I alpha 1 (Col1a1), collagen type IV alpha 2 (Col4a2), collagen type III alpha 1 (Col3a1), collagen type VI alpha 2 (Col6a2), matrix metallopeptidase 14 (Mmp14), and lumican (Lum) (25), were significantly overexpressed in the stellate cells of AEG-1^fl/fl^ livers *versus* AEG-1^ΔMAC^ livers (Fig. 6*B* and Table S4). Col1a1, Col6a2, and Lum were exclusively overexpressed in the stellate cells of AEG-1^fl/fl^ livers (Fig. 6*B*). Metabolic and gene expression changes similar to stellate cells were also observed in the endothelial cells in HF/HSD-fed AEG-1^fl/fl^ livers (Fig. S9*A*). We isolated KC from AEG-1^fl/fl^ and AEG-1^ΔMAC^ mice and treated them with TGFβ inhibitor galunisertib (500 nM) for 24 h. After the treatment, the cells were washed thoroughly, and conditioned media (CM) were collected from these cells and added to HSCs isolated from WT C57BL/6 mice for 12 h. CM from AEG-1^fl/fl^ kC, but not from AEG-1^ΔMAC^ KC, induced expression of Acta2 (α-smooth muscle actin) and Col1a1 in HSCs, which were markedly downregulated upon treatment with galunisertib. These findings from KC-1 and stellate cells suggest that in HF/HSD-fed AEG-1^fl/fl^ livers, KC activation leads to activation of stellate cells and increased fibrosis, which are dampened in AEG-1^ΔMAC^ livers (Fig. 6*D*).

**Figure 7:**
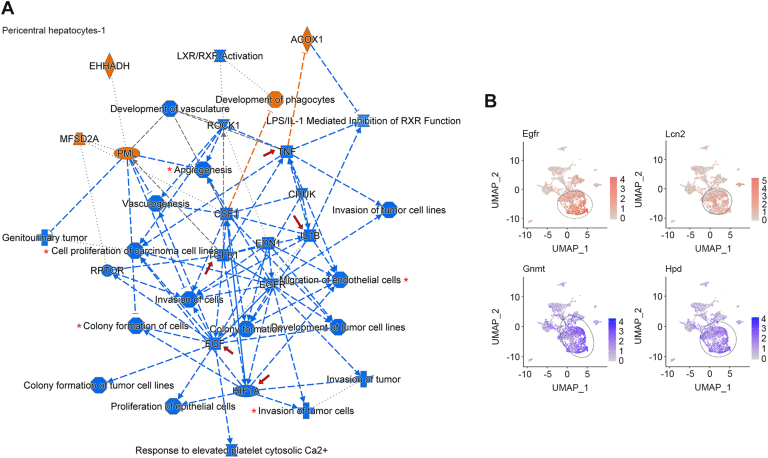
**Single-cell RNA-Seq (scRNA-Seq) identifies inhibition of hepatocyte proliferation and migration in high-fat diet (HFD)/high-sugar diet (HSD)–fed AEG-1^ΔMAC^ mice**. *A*, graphical summary showing activation or inhibition of key regulatory molecules, pathways, functions, and phenotypes in pericentral hepatocytes-1 of HF/HSD-fed AEG-1^ΔMAC^ liver compared with AEG-1^fl/fl^ liver. The shapes and colors are as indicated in Figure 4*C*. *B*, uniform manifold approximation and projection showing expression of the indicated genes in HF/HSD-fed AEG-1^fl/fl^ liver identified by scRNA-Seq. The *circles* show hepatocyte populations.

In the PC hepatocytes of HF/HSD-fed AEG-1^ΔMAC^ livers, the effect was less on metabolism. Rather, a profound inhibition of proliferation and migration and in general development of tumors, along with inhibition of epidermal growth factor and TGFB1, and inhibition of angiogenesis and its regulator HIF1A and inflammatory mediators TNF and interleukin 1 (IL) beta, were observed in PC-1 hepatocytes (*arrows* and *asterisks* in Fig. 7*A*). Similar findings were observed in PC-2 hepatocytes (Fig. S9*B*). Compared with PC hepatocytes, effects were much less pronounced in PP hepatocytes, with inhibition of interferon-γ and inhibitors of nuclear factor kappa B kinase subunit beta and gamma (IKBKB and IKBKG, respectively) regulating inflammation (Fig. S9*C*) in PP-2 hepatocytes of AEG-1^ΔMAC^ livers. No meaningful change was observed in PP-1 hepatocytes (data not shown). Accordingly, tumor-promoting genes, such as epidermal growth factor receptor (Egfr) and lipocalin 2 (Lcn2), were significantly overexpressed, and tumor suppressor genes, such as glycine *N*-methyltransferase (Gnmt) and 4-hydroxyphenylpyruvate dioxygenase (Hpd), were significantly downregulated in the hepatocytes of AEG-1^fl/fl^ livers *versus* AEG-1^ΔMAC^ livers (Fig. 7*B* and Table S4) (26, 27, 28, 29). These analyses indicate that deletion of AEG-1 from myeloid cells exerts a profound effect on other cells in the liver, resulting in inhibition of steatosis, inflammation, and fibrosis.

### Inflammation in mesenteric fat is inhibited in AEG-1^ΔMAC^ mice

**Figure 8:**
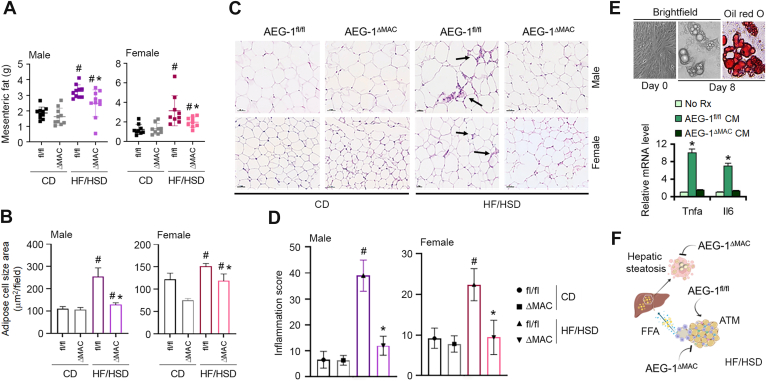
**Inflammation in mesenteric fat is abrogated in high-fat diet (HFD)/high-sugar diet (HSD)–fed AEG-1^ΔMAC^ mice**. *A* and *B*, measurement of mesenteric fat weights (*A*) and adipocyte cell size area (*B*) in the indicated groups at the end of the experiment. For *A* and *B*, data represent mean ± SEM; #*p* < 0.05 *versus* corresponding control diet (CD)–fed mice; ∗*p* < 0.05 between HF/HSD-fed AEG-1^fl/fl^ and AEG-1^ΔMAC^ mice. *C*, representative photomicrographs of formalin-fixed paraffin-embedded (FFPE) sections of mesenteric fat in the indicated groups at the end of the experiment. Magnification: 400x. *Arrows* indicate inflammatory infiltrate. *D*, quantification of inflammatory infiltrate (inflammation score) in mesenteric fat of the indicated groups at the end of the experiment. Data represent mean ± SEM; #*p* < 0.05 *versus* corresponding CD-fed mice; ∗*p* < 0.05 between HF/HSD-fed AEG-1^fl/fl^ and AEG-1^ΔMAC^ mice. *E*, *top*, representative images of stromal vascular fraction (SVF) cells differentiated into adipocytes for 8 days (*top*). Oil Red O staining on day 8 indicates lipid accumulation. *Bottom*, differentiated adipocytes were treated with the indicated conditioned media, and expression of the indicated PICs was detected by quantitative RT–PCR. Data represent mean ± SEM of triplicate experiments. ∗*p* < 0.01 *versus* the other two groups. *F*, *cartoon* showing the role of adipose tissue inflammation in the development of hepatic steatosis in AEG-1^fl/fl^ mice. HF/HSD feeding activates adipose tissue macrophages (ATMs), which induce lipolysis in adipocytes. Free fatty acids (FFAs) are liberated from the adipocytes and enter the liver, where they are stored as triglyceride, causing hepatic steatosis. These events are inhibited in AEG-1^ΔMAC^ mice. PIC, proinflammatory cytokine.

HF/HSD-induced adipose tissue (AT) inflammation plays a central role in MASH development. Mesenteric fat weight and adipocyte cell size area in HF/HSD-fed AEG-1^fl/fl^ and AEG-1^ΔMAC^ littermates of both sexes were significantly higher than those of corresponding CD-fed mice (Fig. 8, *A* and *B*). However, on HF/HSD, both these parameters of AEG-1^ΔMAC^ mice were significantly less than those of AEG-1^fl/fl^ mice (Fig. 8, *A* and *B*). A significantly increased inflammation was observed in HF/HSD-fed AEG-1^fl/fl^ mice compared with AEG-1^ΔMAC^ littermates in both males and females (Fig. 8, *C* and *D*, *arrows* in Figure 8*C* showing inflammatory infiltrates). Stromal vascular fraction was isolated from the inguinal fat pad of C57BL/6 mice and was differentiated into adipocytes (Fig. 8*E*). The adipocytes were treated with CM from macrophages isolated from AEG-1^fl/fl^ and AEG-1^ΔMAC^ mice for 12 h. CM from AEG-1^fl/fl^ macrophages, but not from AEG-1^ΔMAC^ macrophages, induced expression of Tnfa and Il6 in adipocytes (Fig. 8*E*). These findings suggest that in AEG-1^fl/fl^ mice, HF/HSD feeding activates adipose tissue macrophages (ATMs), which induce lipolysis in adipocytes. FFAs are liberated from the adipocytes and enter the liver, where they are stored as TG causing hepatic steatosis (Fig. 8*F*).

## Discussion

The role of AEG-1 in regulating inflammation and macrophage function is well established (8, 17, 30, 31, 32). As such, it is anticipated that hepatic inflammation would be inhibited in HF/HSD-fed AEG-1^ΔMAC^ mice. The intriguing finding from our study is that hepatic steatosis, inflammation, and fibrosis, all are inhibited when AEG-1^ΔMAC^ mice were fed HF/HSD. Indeed, macrophages work both in the AT and in the liver, playing a crucial role in MASH development and progression (33). Following energy imbalance in obesity, adipocyte hypertrophy and local hypoxia induce accumulation of ATMs and instigate a switch in the phenotype of ATMs from an anti-inflammatory to a proinflammatory state (33, 34). In lean AT, anti-inflammatory cytokines secreted by resident ATMs, such as ILs 4 and 10, help maintain insulin sensitivity by counteracting inflammatory responses. Following adipocyte hypertrophy, the secretion of chemoattractants, such as C-C motif chemokine ligand 2 and 3, contributes to macrophage recruitment and production of PICs, including TNFα, IL-6, and IL-1β, which act as the main effectors of impaired adipocyte function and inflammatory signals (33, 34, 35). Adipocytes are highly insulin sensitive, but PICs inhibit insulin action by activating proinflammatory kinases, such as IκB kinase and Janus N-terminal kinase, which phosphorylate and deactivate insulin receptor substrate-1 (34, 36, 37). Inhibition of insulin function results in increased lipolysis, which results in increased release of FFAs in the circulation, with subsequent accumulation in the liver as TG (36, 38, 39). Inflammation of visceral adipose tissue, rather than inflammation of inguinal or subcutaneous white adipose tissue, contributes to obesity-associated metabolic disorders (40, 41). In HF/HSD-fed AEG-1^ΔMAC^ mice, mesenteric fat weight, adipocyte size, and inflammation were significantly less compared with those of HF/HSD-fed AEG-1^fl/fl^ mice (Fig. 8). While WT adipocytes responded to CM from AEG-1^fl/fl^ macrophages by inducing expression of PICs, CM from AEG-1^ΔMAC^ macrophages failed to initiate such a response (Fig. 8*E*).

FFAs are also generated by DNL in the liver and stored as TG (42). Inhibition of FAO contributes to hepatic steatosis, and enzymes regulating DNL, such as FASN, and FAO, such as ACOX1, play an important role in promoting MASH (43, 44). Indeed, hepatic FFA was significantly decreased in the livers of HF/HSD-fed AEG-1^ΔMAC^ mice *versus* AEG-1^fl/fl^ mice (Fig. 2*C*). In addition, in hepatocytes, FASN was decreased, and ACOX1 was increased in the hepatocytes of HF/HSD-fed AEG-1^ΔMAC^ mice. Altogether, our findings suggest that the lack of systemic inflammation in AEG-1^ΔMAC^ mice protects them from HF/HSD-induced MASH.

With CD feeding, the changes observed in the hepatocytes of AEG-1^ΔMAC^ mice were mainly metabolic changes, predominantly changes in lipid and carbohydrate metabolism, leading to inhibition of hepatic steatosis and a decrease in the concentration of sterols (Figs. 3, *F* and *G*, 4*C*, S6, S7, S8). In this scenario, genes activated by the hepatocarcinogen *N*-nitrosodiethylamine were also downregulated in the hepatocytes of AEG-1^ΔMAC^ mice (Figs. 4, *C* and *D*, S6, S7, S8). In the PC hepatocytes of HF/HSD-fed AEG-1^ΔMAC^ mice, metabolic changes were less pronounced; rather, a significant inhibition in genes regulating cell proliferation and migration was observed in these cells (Figs. 7*A*, S9*B*), which is in line with the finding that PC hepatocytes predominantly give rise to HCC (45). These observations indicate that even though overt HCC formation was not detectable with 20 weeks of HF/HSD feeding in AEG-1^fl/fl^ mice, the process has already started at gene expression levels. Accordingly, genes regulating cancer hallmarks, such as proliferation and migration, are robustly upregulated in HF/HSD-fed AEG-1^fl/fl^ mice. Epidermal growth factor receptor overexpression not only contributes to proliferation but also to drug resistance in HCC patients (26), and it is strongly overexpressed in HF/HSD-fed hepatocytes in AEG-1^fl/fl^ mice (Fig. 7*B*). Similar overexpression of LCN2, an iron-sequestering cytokine, which is overexpressed in HCC and protects from ferroptosis (28), was also observed in these hepatocytes (Fig. 7*B*). GNMT, the main enzyme responsible for removing excess *S*-adenosylmethionine, is markedly downregulated in HCC patients, and Gnmt knockout mice develop steatosis and HCC (27). HPD is responsible for tyrosine catabolism, and HPD downregulation leads to tyrosine accumulation and eventually HCC development (29). Both these key enzymes, which function as tumor suppressor genes, are downregulated in HF/HSD-fed hepatocytes in AEG-1^fl/fl^ mice (Fig. 7*B*). In AEG-1^ΔMAC^ mice, the lack of inflammation precludes a favorable environment for HCC development. Concordantly, there is decreased expression of genes regulating HCC in the hepatocytes of AEG-1^ΔMAC^ mice. Under CD feeding, deletion of AEG-1 from myeloid cells, which protects from systemic inflammation, improves hepatocyte function by preventing hepatic steatosis, inflammation, and tumorigenesis. This observation further supports the dogma that neutraceuticals, having anti-inflammatory properties, exert a beneficial effect in normal individuals, protecting them from metabolic dysfunction–associated diseases (46).

One important question is how deletion of AEG-1 in myeloid cells alters gene expression in hepatocytes and other nonparenchymal cells in the liver. PPARA activation is an intrinsic AEG-1 function and is expected in AEG-1-deleted KC. However, PPAR activation is also observed in hepatocytes, stellate, and endothelial cells in the liver of AEG-1^ΔMAC^ mice. Similarly, inhibition of PICs is observed not only in KC but also in hepatocytes and stellate cells, in the livers of AEG-1^ΔMAC^ mice. One potential explanation is that AEG-1 is induced by PICs (47, 48). AEG-1 was originally cloned as a TNF-inducible gene (47). In AEG-1^fl/fl^ mice, PICs released from macrophages might induce AEG-1 in other cells, augmenting intrinsic AEG-1 activity, whereas in AEG-1^ΔMAC^ mice, the lack of PICs would preclude AEG-1 induction in hepatocytes and other nonparenchymal cells.

We previously documented that RNAi-mediated AEG-1 inhibition using hepatocyte-targeted nanoparticles protected mice from HFD-induced MASH (8). Our present findings suggest that siRNA for AEG-1 delivered *via* macrophage-targeted nanoparticles might also provide similar protection. Targeting AEG-1 in both hepatocytes and macrophages might provide added benefit. Our present studies pave the way for the evaluation of these strategies as a future direction.

## Experimental procedures

### Mouse

All animal studies were approved by the Institutional Animal Care and Use Committee at Virginia Commonwealth University (Richmond, VA) and were performed in accordance with the Animal Welfare Act and Regulations (USDA) and the PHS Policy on Humane Care and Use of Laboratory Animals. The generation and characterization of myeloid cell-specific conditional AEG-1 knockout mouse (AEG-1^ΔMAC^) using Cre-*loxP* transgenic recombinase technology has been described (17). Floxed AEG-1 mice (AEG-1^fl/fl^) were developed in the C57BL/6/J background (B6.129P2-*Lyz2*^*tm1(cre)lfo*^/J; Jackson Laboratories) (30), followed by crossing with LysM/Cre mouse (49) to generate AEG-1^ΔMAC^ mouse. For the HF/HSD experiments, both male and female (n = 11) mice were fed ad libitum with irradiated HF/HSD containing 40 kcal% fat, 20 kcal% fructose, and 2% cholesterol or matched control (CD) containing 10 kcal% fat for 20 weeks starting at 7 weeks of age (Research Diets, catalog nos.: D09100310i and D09100304i). At the end of the experiment, blood, liver, and experimental organs were collected from the sacrificed animals. Hepatic panel test was performed by the VCU Molecular Diagnostic Laboratory, Department of Pathology, using standard procedures.

### Isolation of cells and culture conditions

Stromal vascular fraction was isolated from inguinal fat pad of WT C57BL/6/J mice and differentiated into adipocytes using 5 μg/ml insulin (Sigma–Aldrich; catalog no.: I9278), 1 μM dexamethasone (Sigma–Aldrich; catalog no.: D1756), 0.5 mM 3-isobutyl1-methylxanthine (Sigma–Aldrich; catalog no.: I5879), and 125 μM indomethacin (Sigma–Aldrich; catalog no.: I7378) as described (50). Bone marrow–derived macrophages, KCs, and HSCs were isolated and cultured as described (17, 51, 52, 53). Galunisertib was obtained from MedChemExpress (catalog no.: HY-13226).

### Histopathology and immunohistochemistry

Liver tissues were fixed in 10% neutral-buffered formalin and embedded in paraffin. Formalin-fixed paraffin-embedded (FFPE) sections were prepared in VCU Tissue and Data Acquisition and Analysis Core facility and stained with H&E. Immunohistochemistry was performed on these sections as described previously (54) using primary antibodies, such as anti–PLIN2/adipophilin (rabbit; Novus Biologicals; catalog nos.: NB110-40877; 1:200 dilution); F4/80 antibody (rat; Bio-Rad; catalog no.: MCA497R; 1:100 dilution) and antineutrophil antibody (rat; Abcam; catalog no.: ab2557; 1:100 dilution). H-scoring (% positive cells x intensity) was used to quantify individual markers within each region of interest (55). Images were captured using an Olympus BX41 microscope.

### Sirius red staining

Sirius red staining was performed on FFPE tissue sections as described (8). Nuclei staining was done using Weigert’s (Abcam; catalog no.: ab24588) hematoxylin for 8 min, followed by washing with running tap water for 10 min. Incubation with 1.3% aqueous picric acid solution containing 0.1% Sirius red F3B or Direct red 80 (Sigma; catalog no.: 365548) for 1 h at room temperature was performed, followed by washing with 0.5% acidified water (glacial acetic acid solution) for two times, each for 5 min. Slides were dehydrated using 100% ethanol for three times each for 2 min and mounted.

### Measurement of hepatic cholesterol, TGs, and FFAs

Cholesterol assay (Abcam; catalog no.: ab65390) and TG assay (Abcam; catalog no.: ab65336) were performed to quantify TC, HDL, VLDL, FC, and total TG from the flash-frozen liver tissue stored at −80 °C and blood plasma samples as per the manufacturer’s protocol of fluorometric assay. The liver tissue (50 mg) was used for chloroform-free method lipid extraction analysis as per the protocol (Abcam; catalog no.: ab211044). The extracted liquid lipid was dried and resuspended in buffer and sonicated as per the protocol. To quantify the FFA, sonicated lipid samples were used with a modification in the procedure for fatty acid isolation using 150 μl of fatty acid assay buffer (Abcam; catalog no.: ab65341), as per the manufacturer’s protocol of fluorometric assay used for palmitic acid standard preparation.

### Total RNA extraction, complementary DNA preparation and quantitative RT–PCR

Total RNA was extracted using the QIAGEN miRNeasy Mini Kit (QIAGEN). Complementary DNA preparation was done using the ABI Complementary DNA Synthesis Kit (Applied Biosystems). Quantitative RT–PCR was performed using an ABI ViiA7 fast real-time PCR system and TaqMan gene expression assays according to the manufacturer’s protocol (Applied Biosystem).

### Spatial transcriptomics

ST analysis was performed on liver sections of CD-fed AEG-1^fl/fl^ and AEG-1^ΔMAC^ littermates. 10X Genomics experimental workflows were described in our previous publication (56). Mice tissue preparations, including liver tissue collection and slide mounting, were performed according to protocol CG000408 (Rev A). RNA was isolated using the RNeasy FFPE kit (Qiagen; catalog no.: 73504), and quality control was assessed by the RNA 6000 Pico Kit (Agilent; 5067-1513). Following the tissue adhesion test according to protocol CG000408 (Rev A), fixation, staining, imaging, and decrosslinking were performed with tissue sections placed on the Visium Spatial Gene Expression slide as per the protocol CG000409 (Rev C). Visium Spatial for FFPE Gene Expression Kit, Mouse Transcriptome (PN-1000339), and user guide CG000407 (Rev D) were utilized for probe hybridization steps to construct the spatial transcriptome library. The loaded library was sequenced using the Illumina NextSeq 2000 system to read pairs per sample. Bioinformatics analysis was conducted in the following steps. Briefly, raw reads including spatial context from the 10x Visium platform were processed using Space Ranger v2.0.1 with the GEX-mm10 to 2020-A reference genome and probe set to generate gene-count matrices and spatial mapping, resulting in over 2000 spatial spots per tissue sample. Filtered matrices were imported into R using the Seurat package (v5). Spots with read counts over 50,000, fewer than 100 features, or more than 7000 features were removed. Gene expression was first normalized by SCTransformation, which applied regularized negative binomial regression to remove technical variation while preserving biological signals. Samples were merged into a single R object for joint dimensionality reduction and clustering. Dimensionality was reduced to 50 principal components using principal component analysis. This is followed by nearest neighborhood graph construction with shared nearest neighbor and unsupervised clustering of spots with the Louvain algorithm. Clusters were visualized using uniform manifold approximation and projection and spatial plots. For each cell population, differential expression analysis between the two conditions was performed using MAST (https://github.com/RGLab/MAST). To identify major cell types and liver zonation, spots were reclustered using gene markers with high specificity for cell type and zonation pattern in the liver tissue, allowing identification of KCs, cholangiocytes, stellate cells, and the three liver zones. We used Seurat's FindMarkers function for identifying the DEGs for each cluster. Comparisons were performed between each cluster and all remaining cells using the default Wilcoxon rank-sum test. To mitigate the effects of sparsity and reduce computational cost, genes were prefiltered to require detection in at least 25% of cells in either of the two populations (min.pct = 0.25) and a minimum log-fold change of 0.25 (logfc.threshold = 0.25). Markers were subsequently prioritized based on their adjusted *p* values and average log-fold change. The Gene Expression Omnibus (GEO) Series accession number of the dataset is GSE302842 (reviewer token: ahqjcsouvrwbxat).

### Single-cell RNA-Seq

Liver tissues from HF/HSD-fed AEG-1^fl/fl^ and AEG-1^ΔMAC^ mice were used for scRNA-Seq analysis as described in our previous publication with minor modifications (57). Following the 10X Genomics Chromium Fixed RNA Profiling protocol CG000553 (Rev B) and CG000477 (Rev C) outlines for flash-frozen stored tissue, liver tissues were collected, weighing 20 to 25 mg, for fixation.

#### Tissue mincing, fixation, and dissociation

Tissues were minced into small pieces with a blade decontaminated using RNAaseZAP solution (Invitrogen; catalog no.: AM9780). 10X Genomics Next Gel Bead in Emulsion (GEM) Single Cell Fixed RNA Sample Preparation Kit (PN1000414) was used in the following steps. Tissue pieces were suspended in freshly prepared diluted fixation buffer for 16 to 24 h at 4 °C. After centrifugation (3200*g*, 10 min) and removal of fixation buffer, the tissue pieces were resuspended in Quenching buffer. The tissues were dissociated in 2 ml Liberase TL (Millipore, Sigma; catalog no.: 5401020001) diluted in RPMI1640 media (Gibco, Thermo Fisher Scientific; catalog no.: 11875093) to a concentration of 0.02 mg/ml and incubated at 37°C using a thermomixer for 45 min at 500 rpm. The tissue was manually triturated using a P1000 pipette approximately every 5 to 10 min, until the solution became cloudy. The sample was passed through a 40 μm filter (Corning, Lifesciences; catalog no.: 431750) to obtain a single-cell suspension. Cells were counted using a Corning CytoSMART cell counter to ensure the cell number was within the required range for hybridization.

#### Probe hybridization, GEM generation, and barcoding

The following steps were performed using the following 10X Genomics kits: Chromium Next GEM Single Cell Fixed RNA Mouse Transcriptome Probe kit (PN1000490) and Chromium Fixed RNA kit, Mouse Transcriptome (PN1000495). As per the 10X Genomics protocol (CG000477, Rev C) guidelines, probe hybridization steps were performed by resuspending the cell pellets (approximately 1 x 10^6^ cells/reaction) with Hyb Mix and Mouse WTA Probes BC001 (PN2000703) for 16 to 24 h at 42 °C in a thermomixer (heated lid, no shaking). Posthybridization procedures were completed by washing and resuspending the tissue pellet three times in Post-Hyb Buffer (PN2000533) solution. Following the wash steps, the recovered cells were quantified using Ethidium Homodimer-1 (Thermo Fisher Scientific; catalog no.: E1169) and Countess II FL Automated cell counter with red fluorescence protein positive intensity to determine the accurate concentration of the cells. GEMs were generated using the 10X Genomics Chromium Controller and the Chromium Next GEM Chip Q, with a targeted cell recovery of 4000 cells. GEMs were broken with recovery agent (PN220016), preamplification PCR was performed, and DNA purified with SPRIselect reagent (Beckman Coulter; catalog no.: B23317). Cycle number for the sample index PCR was determined using a QuantStudio 3, KAPA SYBR FAST quantitative PCR master mix/ROX Low dye (Roche; catalog no.: KK4600), and TS Primer Mix A (10X Genomics; catalog no.: 2000447). The library was constructed by running the sample index PCR program with unique primer index sets from the Dual Index Plate TS set A (PN3000511) for 14 cycles. SPRIselect reagent was used at 1X ratio to separate small fragments from the PCR product and insert size determined by Agilent Bioanalyzer high-sensitivity chip for library quantification. GEX library sequencing was performed by using Illumina NextSeq2000 and Cell Ranger, version 7.0.1 was used to align the sequence reads against the Gex-mm10 to 2020-A reference genome, producing approximately 200 million reads per sample. In summary, scRNA-Seq data were analyzed using the Seurat (V5) R package. We performed quality control and data processing by removing genes expressed in fewer than three cells and filtering out low-quality cells with fewer than 100 expressed genes or more than 10% mitochondrial transcripts. Subsequently, the count matrices were log-transformed to normalize the data. Next, we identified highly variable genes in each sample using the variance-stabilizing transformation and then applied the anchor-based integration to minimize batch effects and technical variability across samples. Following integration, we employed principal component analysis to reduce the dimensionality of the data to 50 principal components. We subsequently grouped cells into 14 distinct clusters using shared nearest neighbor and the Louvain algorithm. Cell-type identities were assigned through a combination of scType-based prediction and manual annotation using literature-based marker genes (58). Differential expression analysis was conducted with MAST, with significance defined as *p*-value <0.05 and absolute log2 fold change >0.25, revealing transcriptional differences across samples. The GEO Series accession number of the dataset is GSE302723 (reviewer token: ghajkweevxwjrop).

### R program data visualization and IPA

Heatmaps, GO bar plots, volcano plots, cnetplots, and dot plots data analyses were done using RStudio program (version 4.4.1; https://posit.co/products/open-source/rstudio) and Bioconductor packages. IPA was performed using the in-built analysis software to determine the canonical pathways and interactive gene regulators.

### Statistical analysis

Statistical analysis was performed using GraphPad Prism, version 9 software (GraphPad Software, Inc). All calculations were analyzed at the level of significance *p* < 0.05 using Tukey's multiple comparisons test of two-way ANOVA.

## Data availability

All data are contained within the article. The GEO Series accession number of the ST dataset is GSE302842 (reviewer token: ahqjcsouvrwbxat). The GEO Series accession number of the scRNA-Seq dataset is GSE302723 (reviewer token: ghajkweevxwjrop).

## Supporting information

This article contains supporting information.

## Conflict of interests

The authors declare that they have no conflicts of interest with the contents of this article
