# Supplementary material for: Myeloid AEG-1/MTDH drives inflammation and hepatocellular dysfunction in diet-induced steatohepatitis
Source: J Biol Chem. 2025 Dec 23;302(2):111101. doi: 10.1016/j.jbc.2025.111101 (PMC12818226; doi:10.1016/j.jbc.2025.111101)
Supplement: Supplementary Material [file mmc1.pdf]

Supplementary Figure Legends

Figure S1. Representative photographs of livers of the indicated groups at the end of the experiment.

Figure S2. Representative images of hematoxylin eosin (H & E)-stained formalin-fixed paraffin embedded (FFPE) liver sections of control diet (CD)-fed AEG-$1^{fl/fl}$ and AEG-$1^{\Delta MAC}$ littermates.

Figure S3. Gene Ontology Cnetplot showing interactions of DEGs shown in Fig. 3D with functional networks, such as biosynthetic processes (A), homeostasis (B) and proliferation and differentiation (C) in CD-fed AEG-$1^{\Delta MAC}$ mice.

Figure S4. Hepatocytes are distributed in three distinct zones in CD-fed AEG-$1^{fl/fl}$ and AEG-$1^{\Delta MAC}$ livers. A. Violin plots of zone-specific gene markers in different zones of AEG-$1^{fl/fl}$ mouse livers. B. Uniform manifold approximation and projection (UMAP) plots of three zones of CD-fed AEG-$1^{fl/fl}$ and AEG-$1^{\Delta MAC}$ livers. C. Distribution of zone-specific hepatocytes in CD-fed AEG-$1^{fl/fl}$ and AEG-$1^{\Delta MAC}$ livers based on expression of zone-specific gene markers.

Figure S5. Non-parenchymal cells are distributed evenly in CD-fed AEG-$1^{fl/fl}$ and AEG-$1^{\Delta MAC}$ livers. A. Violin plots of indicated cell type-specific gene markers in different cell types of AEG-$1^{fl/fl}$ mouse livers. B. Uniform manifold approximation and projection (UMAP) plots of different cell types of CD-fed AEG-$1^{fl/fl}$ and AEG-$1^{\Delta MAC}$ livers. Other indicates predominantly hepatocytes. C. Distribution of principal non-parenchymal cells in CD-fed AEG-$1^{fl/fl}$ and AEG-$1^{\Delta MAC}$ livers based on expression of cell-type specific gene markers.

Figure S6. Graphical summary showing activation or inhibition of key regulatory molecules, pathways, functions and phenotypes in Cluster 0, representing periportal (zone 1) with Kupffer cells, and Cluster 1, representing midlobular (zone 2), of CD-fed AEG-$1^{\Delta MAC}$ liver compared to AEG-$1^{fl/fl}$ liver. Orange color indicates activation z-score >2, while blue color indicates inhibition z-score <2. The shapes indicate the following: octagon: function; cross: disease; inverted triangle:

kinase; ellipse: transcription regulator; square: cytokine; trapezoid: transporter; circle: molecule of other class; hour glass: canonical pathway.

Figure S7. Graphical summary showing activation or inhibition of key regulatory molecules, pathways, functions and phenotypes in Cluster 2, representing periportal (zone 1) with Kupffer cells, and Cluster 3, representing pericentral (zone 3) close to midlobular (zone 2) with stellate cells, of CD-fed AEG-1$^{\Delta MAC}$ liver compared to AEG-1$^{fl/fl}$ liver. The shapes and colors are as indicated in Fig. S6.

Figure S8. Graphical summary showing activation or inhibition of key regulatory molecules, pathways, functions and phenotypes in Cluster 5, representing pericentral (zone 3) close to midlobular (zone 2), and Cluster 6, representing periportal (zone 1) with cholangiocytes, of CD-fed AEG-1$^{\Delta MAC}$ liver compared to AEG-1$^{fl/fl}$ liver. The shapes and colors are as indicated in Fig. S6.

Figure S9. Graphical summary showing activation or inhibition of key regulatory molecules, pathways, functions and phenotypes in endothelial cells (A), pericentral hepatocytes-2 (PC-2) and periportal hepatocytes-2 (PC-2) of HF/HSD-fed AEG-1$^{\Delta MAC}$ liver compared to AEG-1$^{fl/fl}$ liver. The shapes and colors are as indicated in Fig. S6.

Figure S1

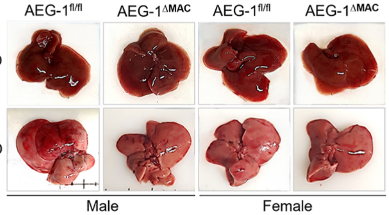

Figure S2

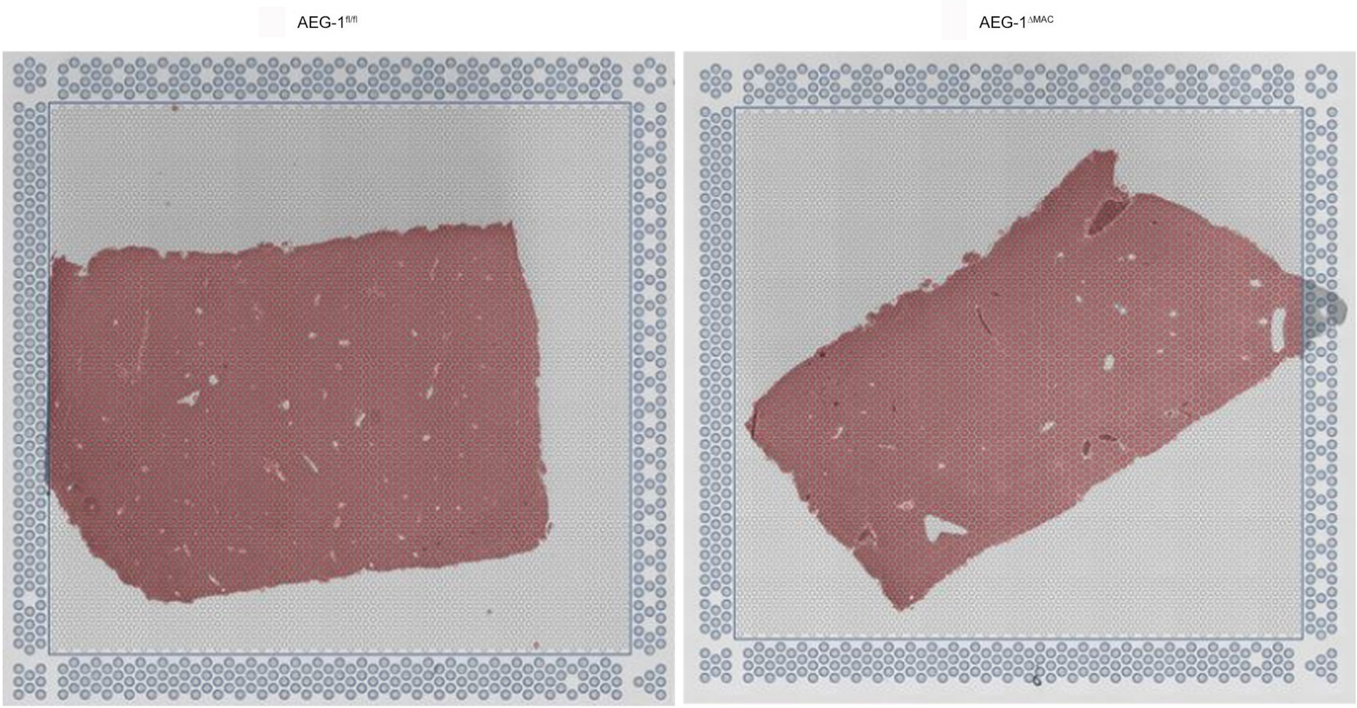

Figure S3

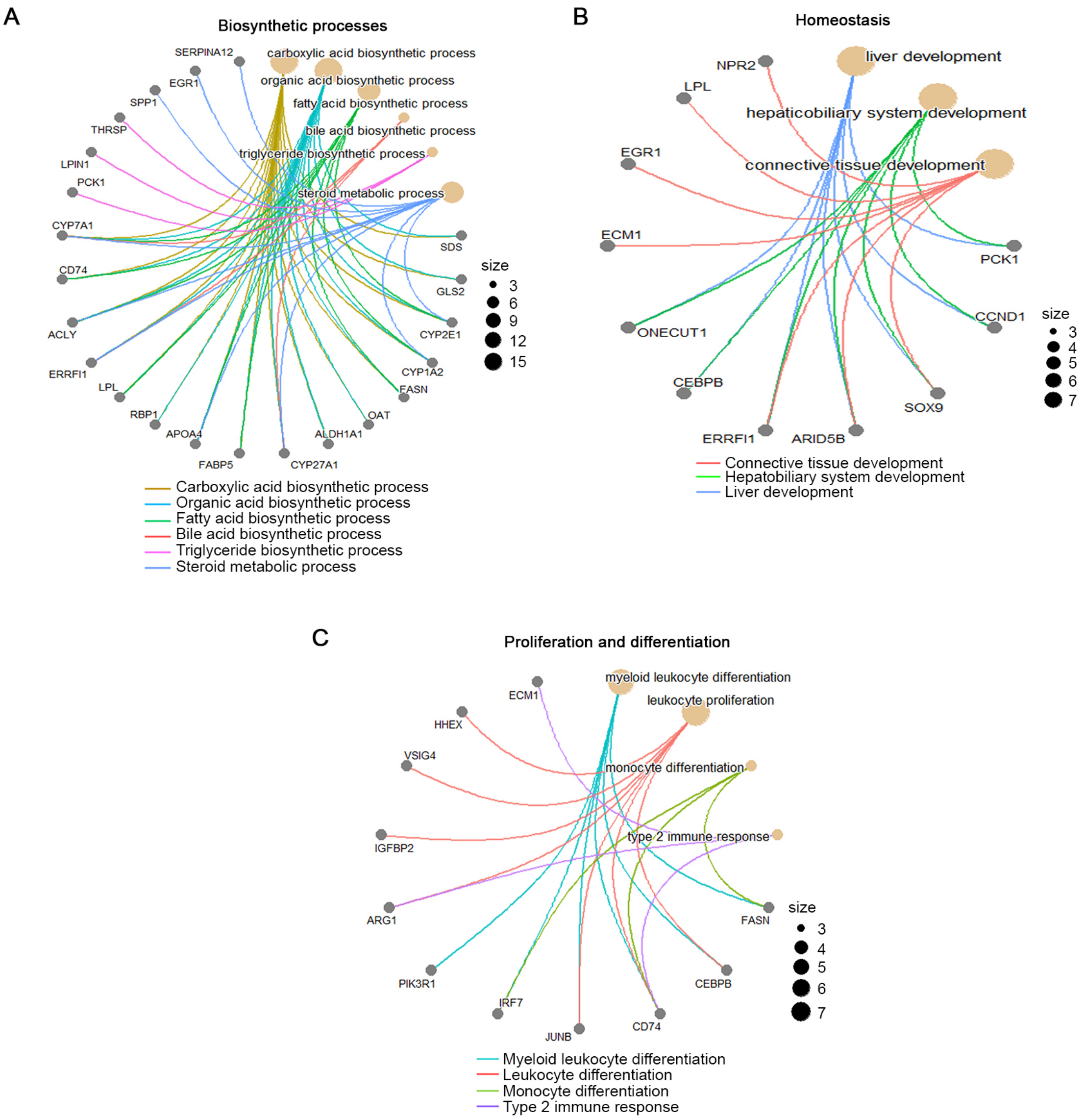

A
Biosynthetic processes
carboxylic acid biosynthetic process
organic acid biosynthetic process
fatty acid biosynthetic process
bile acid biosynthetic process
triglyceride biosynthetic process
steroid metabolic process
SERPINA12
EGR1
SPP1
THRSP
LPIN1
PCK1
CYP7A1
CD74
ACLY
ERRFI1
LPL
RBP1
APOA4
FABP5
CYP27A1
ALDH1A1
OAT
FASN
CYP1A2
CYP2E1
GLS2
SDS
size
3
6
9
12
15
Carboxylic acid biosynthetic process
Organic acid biosynthetic process
Fatty acid biosynthetic process
Bile acid biosynthetic process
Triglyceride biosynthetic process
Steroid metabolic process
B
Homeostasis
liver development
hepaticobiliary system development
connective tissue development
NPR2
LPL
EGR1
ECM1
ONECUT1
CEBPB
ERRFI1
ARID5B
SOX9
CCND1
PCK1
size
3
4
5
6
7
Connective tissue development
Hepatobiliary system development
Liver development
C
Proliferation and differentiation
myeloid leukocyte differentiation
leukocyte proliferation
monocyte differentiation
type 2 immune response
ECM1
HHEX
VSIG4
IGFBP2
ARG1
PIK3R1
IRF7
JUNB
CD74
CEBPB
FASN
size
3
4
5
6
7
Myeloid leukocyte differentiation
Leukocyte differentiation
Monocyte differentiation
Type 2 immune response

Figure S4

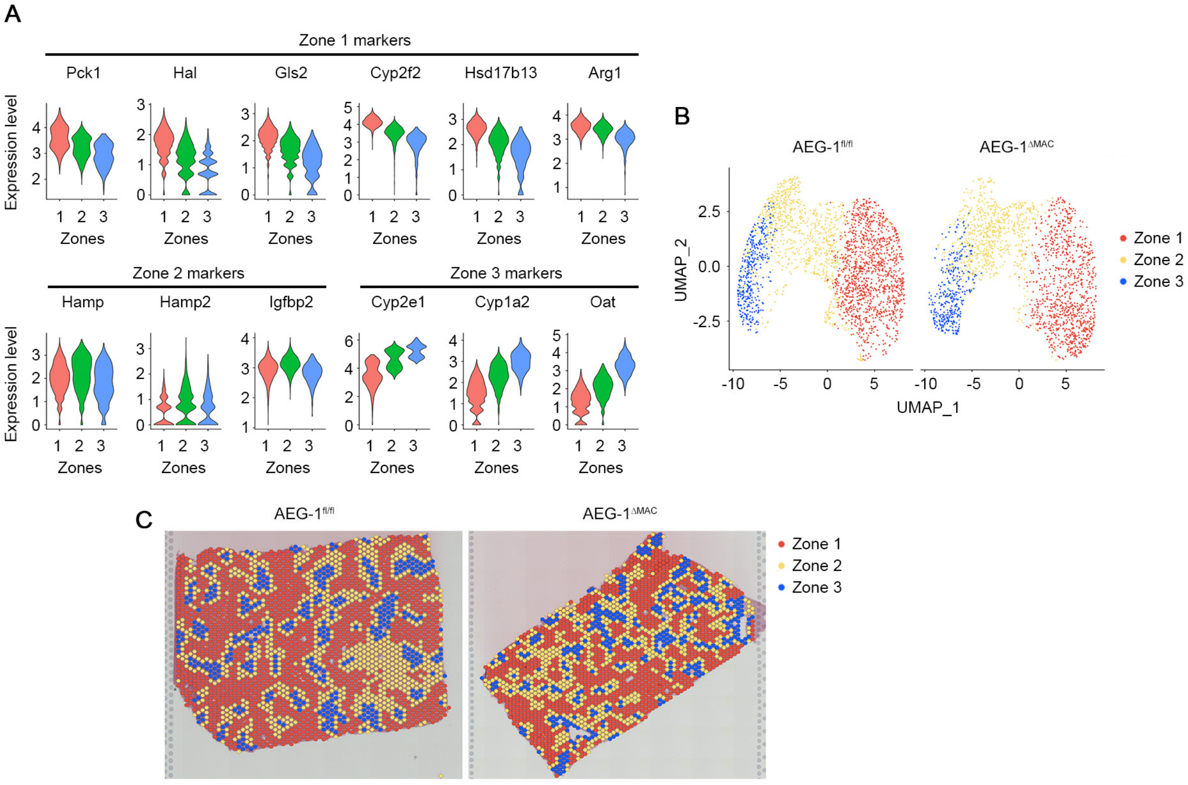

A
Zone 1 markers
Pck1
Hal
Gls2
Cyp2f2
Hsd17b13
Arg1
Expression level
Zones
Zone 2 markers
Hamp
Hamp2
Igfbp2
Zone 3 markers
Cyp2e1
Cyp1a2
Oat
B
AEG-1fl/fl
AEG-1ΔMAC
UMAP_2
UMAP_1
Zone 1
Zone 2
Zone 3
C
AEG-1fl/fl
AEG-1ΔMAC
Zone 1
Zone 2
Zone 3

Figure S5

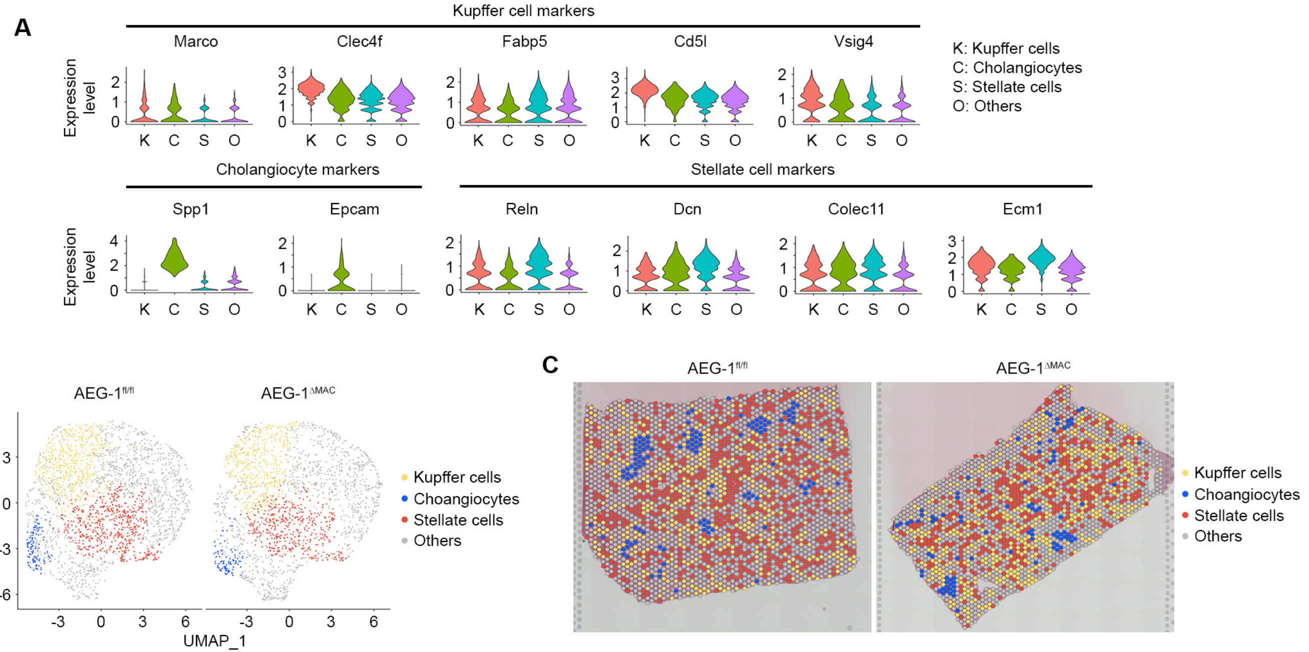
A
Kupffer cell markers
Marco
Clec4f
Fabp5
Cd5l
Vsig4
Expression level
K C S O
K: Kupffer cells
C: Cholangiocytes
S: Stellate cells
O: Others
Cholangiocyte markers
Stellate cell markers
Spp1
Epcam
Reln
Dcn
Colec11
Ecm1
B
AEG-1fl/fl
AEG-1ΔMAC
UMAP_2
UMAP_1
Kupffer cells
Choangiocytes
Stellate cells
Others
C
AEG-1fl/fl
AEG-1ΔMAC
Kupffer cells
Choangiocytes
Stellate cells
Others

Figure S6

Cluster 0

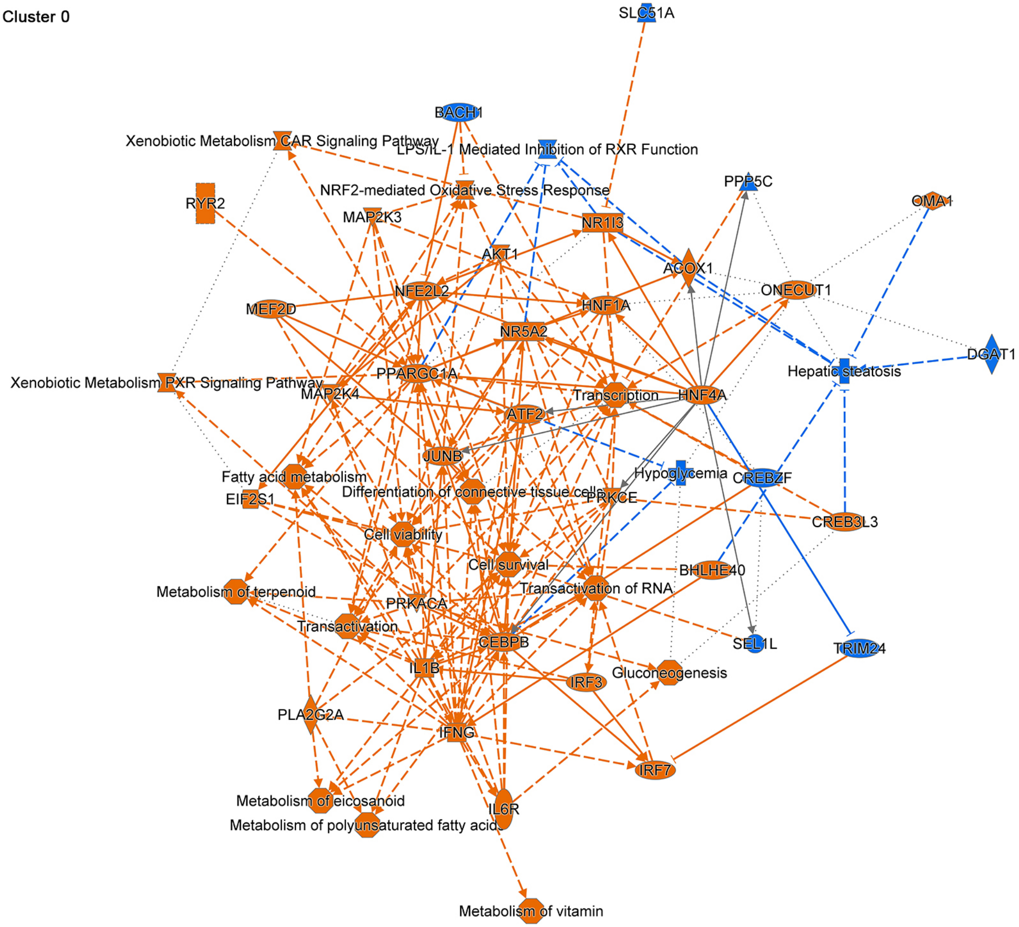

Cluster 1

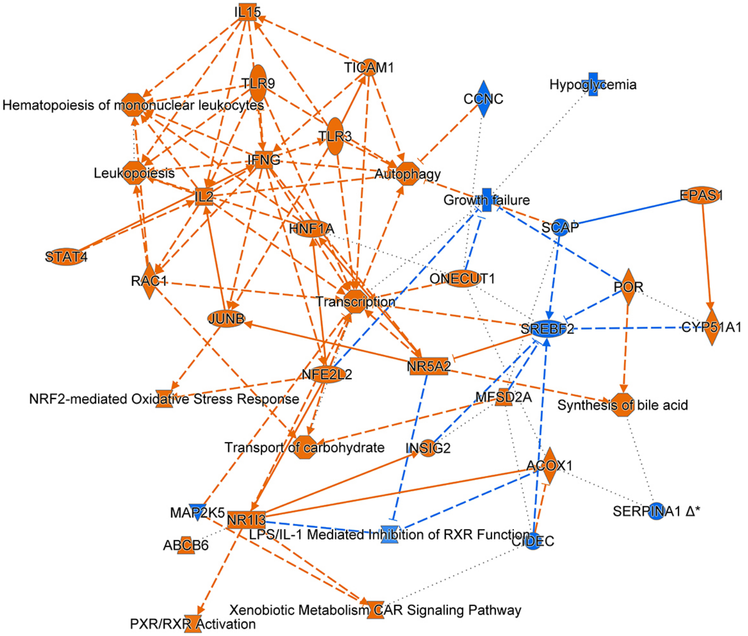

Figure S7

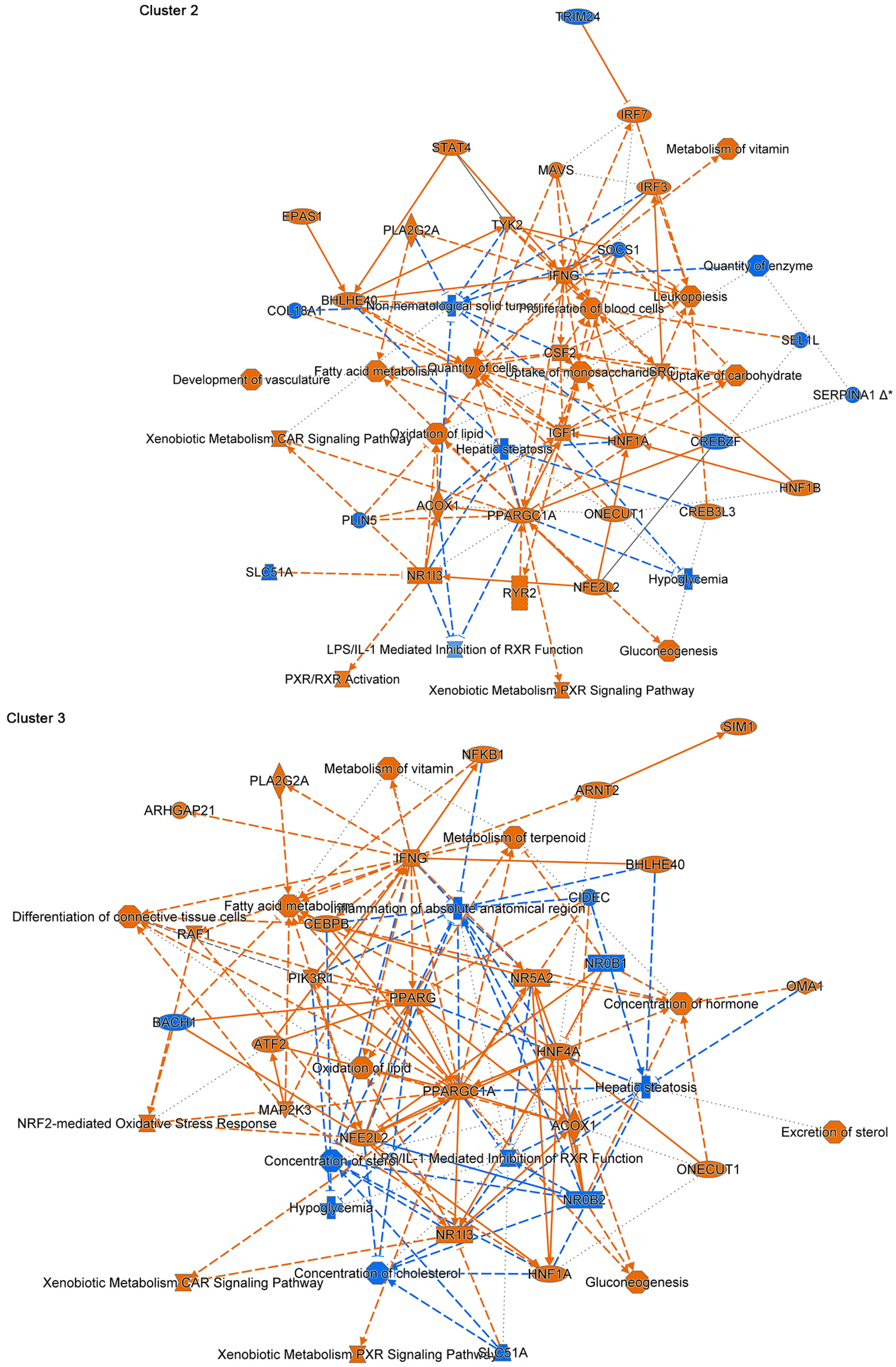
Cluster 2
TRIM24
IRF7
Metabolism of vitamin
STAT4
MAVS
IRF3
EPAS1
PLA2G2A
TYK2
SOCS1
Quantity of enzyme
IFNG
Leukopoiesis
BHLHE40
COL18A1
Non-hematological solid tumor
Proliferation of blood cells
SEL1L
CSF2
Development of vasculature
Fatty acid metabolism
Quantity of cells
Uptake of monosaccharide
SRC
Uptake of carbohydrate
SERPINA1 Δ*
Xenobiotic Metabolism CAR Signaling Pathway
Oxidation of lipid
IGF1
Hepatic steatosis
HNF1A
CREBZF
HNF1B
ACOX1
PLIN5
PPARGC1A
ONECUT1
CREB3L3
SLC51A
NR1I3
RYR2
NFE2L2
Hypoglycemia
LPS/IL-1 Mediated Inhibition of RXR Function
Gluconeogenesis
PXR/RXR Activation
Xenobiotic Metabolism PXR Signaling Pathway
Cluster 3
SIM1
NFKB1
Metabolism of vitamin
PLA2G2A
ARNT2
ARHGAP21
Metabolism of terpenoid
IFNG
BHLHE40
CIDEC
Fatty acid metabolism
Inflammation of absolute anatomical region
Differentiation of connective tissue cells
CEBPB
RAF1
NR0B1
PIK3R1
NR5A2
PPARG
OMA1
Concentration of hormone
BACH1
ATF2
HNF4A
Oxidation of lipid
Hepatic steatosis
PPARGC1A
MAP2K3
NRF2-mediated Oxidative Stress Response
ACOX1
Excretion of sterol
NFE2L2
Concentration of sterol
LPS/IL-1 Mediated Inhibition of RXR Function
ONECUT1
NR0B2
Hypoglycemia
NR1I3
Xenobiotic Metabolism CAR Signaling Pathway
Concentration of cholesterol
HNF1A
Gluconeogenesis
Xenobiotic Metabolism PXR Signaling Pathway
SLC51A

Figure S8

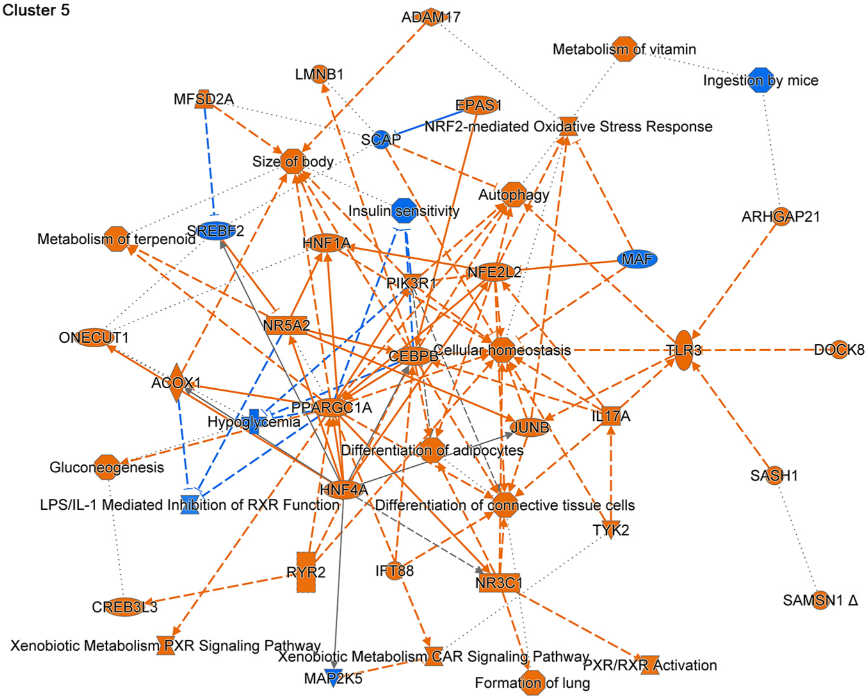
Cluster 5
ADAM17
Metabolism of vitamin
Ingestion by mice
LMNB1
MFSD2A
EPAS1
SCAP
NRF2-mediated Oxidative Stress Response
Size of body
Autophagy
Insulin sensitivity
ARHGAP21
Metabolism of terpenoid
SREBF2
HNF1A
MAF
NFE2L2
PIK3R1
NR5A2
ONECUT1
CEBPB
Cellular homeostasis
TLR3
DOCK8
ACOX1
PPARGC1A
Hypoglycemia
IL17A
JUNB
Differentiation of adipocytes
Gluconeogenesis
SASH1
HNF4A
LPS/IL-1 Mediated Inhibition of RXR Function
Differentiation of connective tissue cells
TYK2
RYR2
IFT88
NR3C1
CREB3L3
SAMSN1 Δ
Xenobiotic Metabolism PXR Signaling Pathway
Xenobiotic Metabolism CAR Signaling Pathway
PXR/RXR Activation
MAP2K5
Formation of lung

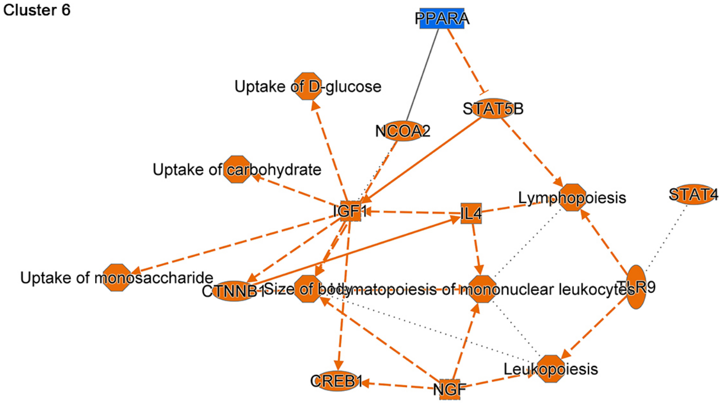
Cluster 6
PPARA
Uptake of D-glucose
STAT5B
NCOA2
Uptake of carbohydrate
Lymphopoiesis
STAT4
IGF1
IL4
Uptake of monosaccharide
CTNNB1
Size of body
Hematopoiesis of mononuclear leukocytes
TLR9
Leukopoiesis
CREB1
NGF

Figure S9

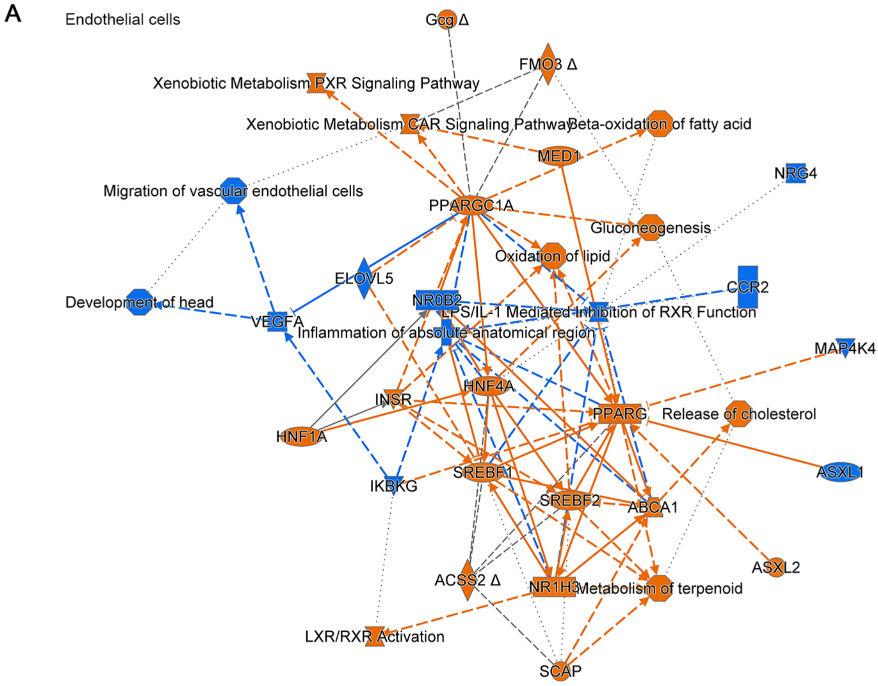
A
Endothelial cells
Gcg Δ
FMO3 Δ
Xenobiotic Metabolism PXR Signaling Pathway
Xenobiotic Metabolism CAR Signaling Pathway
Beta-oxidation of fatty acid
MED1
NRG4
Migration of vascular endothelial cells
PPARGC1A
Gluconeogenesis
Oxidation of lipid
ELOVL5
CCR2
Development of head
NR0B2
LPS/IL-1 Mediated Inhibition of RXR Function
VEGFA
Inflammation of absolute anatomical region
MAP4K4
HNF4A
INSR
PPARG
Release of cholesterol
HNF1A
ASXL1
SREBF1
IKBKG
SREBF2
ABCA1
ASXL2
ACSS2 Δ
NR1H3
Metabolism of terpenoid
LXR/RXR Activation
SCAP

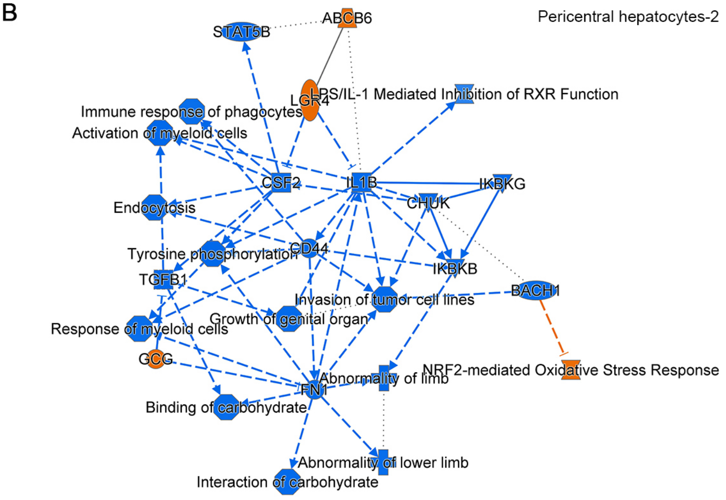
B
ABCB6
Pericentral hepatocytes-2
STAT5B
LGR4
LPS/IL-1 Mediated Inhibition of RXR Function
Immune response of phagocytes
Activation of myeloid cells
CSF2
IL1B
IKBKG
CHUK
Endocytosis
Tyrosine phosphorylation
CD44
IKBKB
TGFB1
BACH1
Invasion of tumor cell lines
Growth of genital organ
Response of myeloid cells
GCG
Abnormality of limb
NRF2-mediated Oxidative Stress Response
FN1
Binding of carbohydrate
Abnormality of lower limb
Interaction of carbohydrate

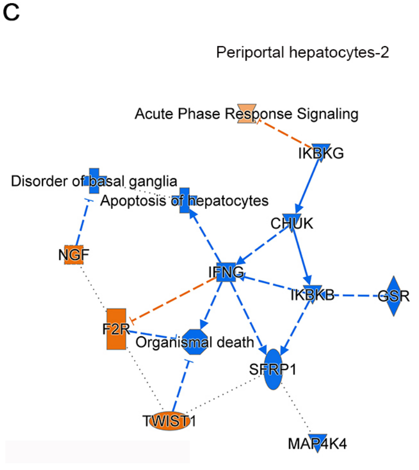
C
Periportal hepatocytes-2
Acute Phase Response Signaling
IKBKG
Disorder of basal ganglia
Apoptosis of hepatocytes
CHUK
NGF
IFNG
IKBKB
GSR
F2R
Organismal death
SFRP1
TWIST1
MAP4K4

Table S1. Highly expressed genes to annotate each cluster in spatial transcriptomics

Gene	p_val_adj	avg_log2FC	Cluster
Hsd17b13	2.25E-66	0.372681573	0
Cyp2f2	1.18E-65	0.338293538	0
Etnppl	3.78E-43	0.318872276	0
Tmsb4x	7.58E-38	0.209050574	0
Cd5l	4.69E-32	0.295288526	0
Clec4f	1.58E-28	0.294070039	0
Apoa4	1.30E-26	0.223429129	0
Hsd17b6	1.06E-20	0.18924821	0
C1qc	5.71E-19	0.253500355	0
Fcna	9.15E-17	0.233117084	0
Sfxn1	1.87E-15	0.182311572	0
Pigr	9.95E-15	0.157961901	0
Mup20	5.44E-13	0.113767446	0
C1qa	7.16E-13	0.209278095	0
Cfp	2.10E-12	0.222160434	0
Marco	6.14E-12	0.23623313	0
Lgmn	6.77E-12	0.198125362	0
Hal	2.15E-10	0.1065128	0
Sds	4.92E-09	0.119361861	0
Clec2h	5.12E-09	0.167907028	0
Vsig4	6.56E-08	0.177769222	0
Sod3	2.31E-07	0.192533456	0
Ctsc	2.45E-07	0.155065093	0
Msr1	8.27E-07	0.156425619	0
Ctss	2.31E-06	0.164216553	0
Cdh1	0.000111807	0.113900148	0
Serpina1e	0.000150441	0.056682765	0
Aldh1b1	0.000239882	0.017844119	0
Alb	0.000321725	0.044999874	0
Gls2	0.000363652	0.053827042	0
Ugt2b38	0.000404827	0.127475205	0
Cryl1	0.00054212	0.112011046	0
Ntn4	0.00067678	0.131496005	0
Adgre4	0.000697145	0.100345816	0
Laptm5	0.000855352	0.124702529	0
Cd68	0.002441264	0.129793833	0
Il18bp	0.00253971	0.116836052	0
Colec11	0.002571637	0.146523586	0
Tstd1	0.002743469	0.095751575	0
Lcp2	0.003902214	0.117028601	0
Fcgr3	0.006254197	0.100572056	0
Afm	0.009515631	0.093382555	0
Gngt2	0.012275755	0.103937892	0
Hpx	0.014717558	0.032618694	0
C1qb	0.024382838	0.113977061	0
Tiam2	0.037731474	0.089268836	0
Slc3a1	0.042041005	0.058095221	0
Csad	3.66E-60	0.538759732	1
Scd1	1.18E-58	0.621215978	1
Igfbp2	8.17E-44	0.344581006	1
Acaa1b	5.28E-40	0.315376541	1
Fabp5	3.10E-29	0.460525201	1
Ehhadh	1.19E-27	0.316083021	1
Tff3	3.75E-27	0.605993936	1
Paqr9	1.99E-26	0.331680224	1
Acss2	1.24E-24	0.349587363	1

Egr1	1.57E-23	0.457464795	1
Rnf125	4.77E-23	0.26920884	1
Acacb	4.64E-21	0.325252126	1
Gsta3	9.37E-21	0.203684069	1
Elovl5	2.27E-20	0.314334527	1
Gstm1	1.15E-19	0.195987842	1
Dbi	2.02E-19	0.194311901	1
Acox1	3.02E-19	0.200830975	1
Serpina1a	1.14E-18	0.196724433	1
Serpina1e	3.90E-18	0.216310192	1
Rgn	7.91E-18	0.182582127	1
Fads1	1.67E-17	0.273259784	1
Ces1e	2.21E-16	0.277784994	1
Osgin1	3.26E-16	0.285681366	1
Fabp1	6.62E-16	0.200746203	1
Gck	1.43E-15	0.262542883	1
Hmgcs1	3.67E-15	0.267667751	1
Coq8a	3.98E-15	0.220682707	1
Car3	1.52E-14	0.170419686	1
H2-Q10	1.73E-14	0.169004716	1
Elovl2	2.85E-14	0.257466732	1
Arrdc3	4.38E-14	0.258503646	1
Cyp4a14	6.50E-14	0.205410688	1
Pcyt2	2.03E-13	0.223509382	1
Ces1d	2.66E-13	0.220020466	1
Cyb5r3	3.52E-13	0.202555514	1
Alb	4.28E-13	0.194545569	1
Dhcr24	7.29E-13	0.219709648	1
Kng1	5.46E-12	0.164162894	1
Fasn	1.06E-11	0.2945792	1
Hgfac	1.08E-11	0.207632541	1
Mt1	1.56E-11	0.199620898	1
Apoa1	1.80E-11	0.145671417	1
Inmt	2.77E-11	0.165096297	1
Atf5	3.65E-11	0.224191323	1
Gpam	8.08E-11	0.248085274	1
Chka	9.60E-11	0.27322165	1
Ephx2	1.00E-10	0.168365852	1
Cps1	1.40E-10	0.14475399	1
Fads2	1.42E-10	0.23803866	1
Fgl1	1.46E-10	0.179183325	1
Akr1c6	1.50E-10	0.19736572	1
Rdh11	2.41E-10	0.253362093	1
Pzp	6.38E-10	0.145141691	1
Ttr	9.81E-10	0.123288142	1
Clpx	1.39E-09	0.195310697	1
Apcs	1.54E-09	0.230629842	1
Rbp1	3.51E-09	0.286491695	1
Aqp8	6.95E-09	0.221007111	1
Hp	8.01E-09	0.149582878	1
Itih2	1.07E-08	0.175369456	1
Acsl5	1.31E-08	0.20235369	1
Pmvk	1.45E-08	0.24016247	1
Scap	1.80E-08	0.217580048	1
Abcd2	3.81E-08	0.213501861	1
Sc5d	3.84E-08	0.205994989	1
Cpt1a	5.56E-08	0.206245059	1
Mbl2	7.17E-08	0.1996984	1
Aldh2	7.67E-08	0.129484296	1

Depp1	7.74E-08	0.230782182	1
Ubc	1.00E-07	0.159925506	1
Acot1	1.00E-07	0.15699467	1
Insig1	1.09E-07	0.192194131	1
Iah1	1.44E-07	0.208739296	1
Ces1g	2.04E-07	0.186220912	1
Cldn1	2.37E-07	0.207553031	1
Gclc	2.47E-07	0.169400112	1
Aldh1a7	2.52E-07	0.1939389	1
Abcd3	2.79E-07	0.146065471	1
Scp2	3.59E-07	0.122813673	1
Hgd	3.68E-07	0.149822279	1
Grb7	3.71E-07	0.212314674	1
Haao	5.35E-07	0.169108532	1
Hamp2	6.28E-07	0.244918347	1
Acly	6.88E-07	0.211662891	1
Lpl	9.94E-07	0.236653204	1
Arg1	1.20E-06	0.124123993	1
Bdh1	1.24E-06	0.164728849	1
Uox	1.58E-06	0.108489417	1
Serpina6	2.18E-06	0.19091075	1
Hspd1	2.28E-06	0.181193539	1
Enho	2.48E-06	0.187542814	1
Lgalsl	3.40E-06	0.192797972	1
Grhpr	3.82E-06	0.158232654	1
Agxt	3.84E-06	0.146197313	1
Mlxipl	4.10E-06	0.184417037	1
Ppib	5.42E-06	0.167273638	1
Psen2	6.21E-06	0.17140355	1
Idh1	6.70E-06	0.147360742	1
Agt	7.58E-06	0.127712883	1
Anp32a	9.77E-06	0.150191409	1
Pctp	1.11E-05	0.178563781	1
Sema4g	1.22E-05	0.193963686	1
Acbd5	1.46E-05	0.166113253	1
Serpina1b	1.61E-05	0.108356474	1
Papss2	1.98E-05	0.185698362	1
Crot	2.07E-05	0.112867517	1
Tm7sf2	2.20E-05	0.168530402	1
Atp5b	2.39E-05	0.131619297	1
Sar1b	2.58E-05	0.142767906	1
Sardh	2.94E-05	0.141464216	1
Lgals9	3.41E-05	0.140520306	1
Uroc1	6.44E-05	0.139065949	1
Gc	6.61E-05	0.115777924	1
Trf	8.22E-05	0.085445783	1
Pglyrp2	8.22E-05	0.16166441	1
Crat	0.000106269	0.1881959	1
Itih3	0.000124428	0.100825249	1
Angptl8	0.000127166	0.178518066	1
Bhmt2	0.000127567	0.136370981	1
Ppara	0.000136711	0.174852255	1
Upb1	0.000136874	0.153168966	1
Hmgcs2	0.000184063	0.123130462	1
Grpel1	0.000189644	0.148643134	1
Qdpr	0.000203425	0.136391135	1
Igfbp1	0.000236995	0.191227299	1
Cyp2c29	0.000243433	0.000826685	1
Gstz1	0.000267007	0.119188809	1

Hsd17b4	0.000272438	0.139329791	1
Cluh	0.000289067	0.141498308	1
Bbox1	0.00033098	0.166378099	1
Dhcr7	0.000357832	0.163441491	1
Hbb-bt	0.00044005	0.056334885	1
F10	0.000636409	0.132963612	1
Ftcd	0.000646851	0.122087018	1
Acat2	0.000747142	0.163821413	1
Plg	0.000872858	0.093434361	1
Kng2	0.000885712	0.140262153	1
Acat1	0.000924976	0.166597859	1
Gm4952	0.001084216	0.130851995	1
Apom	0.00160171	0.137738791	1
Spon2	0.001699021	0.150994916	1
Fgb	0.001706807	0.083549384	1
Cldn3	0.001717834	0.168513024	1
Ccl9	0.002170917	0.167631969	1
Amdhd1	0.002212952	0.09943928	1
Ttc36	0.002511929	0.104798784	1
Acox2	0.002525759	0.128709241	1
Cdo1	0.002597406	0.130364078	1
Bckdha	0.002894538	0.122337068	1
Keg1	0.002988217	0.12015144	1
Ppp1r3c	0.003591998	0.18127776	1
Gchfr	0.00373543	0.152506097	1
Pcbd1	0.003737644	0.124684219	1
Aspg	0.004533721	0.131100695	1
Pon1	0.004589446	0.077903562	1
Decr2	0.004924447	0.128740851	1
Col18a1	0.005713261	0.144560198	1
Khk	0.00629134	0.110638576	1
Acadvl	0.00642045	0.150792909	1
Apoc4	0.007013746	0.095925452	1
Nat8f2	0.007734419	0.125939824	1
Atp5a1	0.008138048	0.099036612	1
Lss	0.010087338	0.156375572	1
Cyp27a1	0.010168901	0.098536071	1
Mat1a	0.010175287	0.103131468	1
Cd302	0.010494381	0.112757151	1
Fgg	0.010507439	0.073182949	1
Serpinf2	0.010732081	0.096191432	1
Gcat	0.011079476	0.15288451	1
Atp5g3	0.011151252	0.091562337	1
Ide	0.01119931	0.146964163	1
Sephs2	0.011335788	0.113474222	1
Gldc	0.011347043	0.13576708	1
Adra1b	0.011824261	0.177283784	1
Pank1	0.012276415	0.124600932	1
Foxa3	0.012425354	0.1553802	1
Atp5mpl	0.013472122	0.138626529	1
Glud1	0.013522713	0.100281421	1
Nlrp6	0.013738429	0.142015244	1
Uqcr11	0.013805769	0.133553571	1
Fbp1	0.014328292	0.060613732	1
Gstm7	0.014497649	0.142063235	1
Ces1f	0.015225106	0.121131961	1
Aldh7a1	0.015369943	0.119794836	1
Aldh8a1	0.015375454	0.112139803	1
Prlr	0.015518084	0.132566188	1

Apof	0.016000339	0.088978969	1
Txndc5	0.016112805	0.127063303	1
Alas2	0.016141523	0.131745553	1
Mif4gd	0.01615812	0.146269005	1
Slc17a8	0.016241606	0.115109511	1
Ahsg	0.016304854	0.077494139	1
Hnrnpab	0.016710745	0.144281105	1
Prodh2	0.017408647	0.141475068	1
Abcg8	0.018077181	0.162906423	1
Sec14l2	0.018160576	0.102944597	1
Fga	0.018206898	0.074067385	1
Hmgcl	0.019025776	0.115110777	1
Ces2a	0.02050521	0.102470815	1
Itih1	0.020867027	0.146004401	1
Pnpla7	0.021169268	0.140141667	1
Cdkn1a	0.02214252	0.146836904	1
Msrb1	0.02349142	0.110019065	1
Eef2	0.024025158	0.127201934	1
Me1	0.025185877	0.138298089	1
Nipsnap1	0.027749484	0.122794886	1
Sntb1	0.029381937	0.123625665	1
Etfb	0.029690128	0.124532038	1
Alad	0.029782987	0.120570597	1
Spc24	0.031511361	0.150798888	1
Tmem150a	0.032068258	0.136901903	1
Copz1	0.033161292	0.144219873	1
F2	0.033979824	0.078855346	1
Dpp9	0.035812674	0.145699414	1
Nr0b2	0.037715903	0.142948365	1
Hsd3b7	0.041555588	0.109163762	1
Sptbn2	0.048320082	0.147303927	1
Nit2	0.048453843	0.131710481	1
Cyp2f2	3.48E-162	0.826596554	2
Gls2	8.68E-162	0.834471262	2
Hal	4.64E-147	0.851212193	2
Uroc1	1.92E-145	0.733776871	2
Pck1	3.28E-125	0.825262118	2
Gstp1	8.83E-125	0.570343108	2
Hpx	4.04E-122	0.565453859	2
Ftcd	1.57E-117	0.659467192	2
Amdhd1	2.31E-113	0.645079388	2
Arg1	4.19E-108	0.534807488	2
Fbp1	1.20E-107	0.528148617	2
Pigr	2.54E-97	0.624212217	2
Aspg	3.77E-95	0.607867337	2
Hsd17b6	5.46E-89	0.748334499	2
Sds	1.29E-87	0.727477257	2
Hsd17b13	7.61E-87	0.576150488	2
Got1	7.67E-87	0.626832297	2
Gldc	2.35E-85	0.66907663	2
Etnppl	2.45E-77	0.592327291	2
Cps1	2.91E-75	0.427934694	2
Asl	4.67E-71	0.506066317	2
Mup20	3.93E-70	0.507900726	2
Hp	3.46E-69	0.437465829	2
Rida	6.62E-69	0.409946004	2
Aldh1b1	2.49E-66	0.764670922	2
Aox3	2.58E-66	0.477945731	2

Uox	6.98E-62	0.343552722	2
Agxt	1.05E-61	0.493830714	2
Alb	1.11E-60	0.366540478	2
Cth	2.34E-59	0.471805877	2
Slc3a1	2.08E-58	0.598480662	2
Sephs2	7.86E-58	0.460737907	2
Gc	5.77E-55	0.404754273	2
Hbb-bs	2.01E-54	0.774957267	2
Itih3	1.36E-52	0.343422009	2
Kng1	1.37E-51	0.3702537	2
Atp5b	2.16E-50	0.378399717	2
Aldob	7.06E-50	0.344097012	2
Hba-a2	1.02E-49	0.719708772	2
Atp5a1	2.23E-49	0.376255768	2
Scp2	2.43E-49	0.346784596	2
Tdo2	5.50E-46	0.332427609	2
Sfxn1	8.23E-45	0.439665708	2
Sdsl	1.07E-44	0.405271899	2
C8a	1.27E-44	0.385497929	2
C9	1.49E-44	0.358426389	2
Trf	1.58E-43	0.274823962	2
H2-Q10	1.03E-42	0.296923028	2
Gas2	3.17E-42	0.448246284	2
Aass	4.17E-42	0.405822605	2
Slc38a3	4.42E-42	0.332308674	2
Slc25a47	1.04E-41	0.389054614	2
Selenbp2	1.50E-41	0.403212714	2
C3	2.78E-39	0.280394801	2
Otc	2.86E-39	0.400522511	2
Selenop	1.11E-38	0.289702731	2
Rhou	1.20E-37	0.406973367	2
Aldh1l1	4.30E-37	0.331107637	2
Cdh1	7.88E-37	0.44509582	2
Serpina1a	1.03E-36	0.308303342	2
Qsox1	1.33E-35	0.36058315	2
Atp5c1	1.39E-35	0.384598416	2
Herpud1	1.36E-34	0.412396322	2
Nnmt	5.27E-34	0.388445745	2
Fasn	1.21E-32	0.403300265	2
Tent5c	7.72E-32	0.320620577	2
Acly	1.74E-31	0.34449549	2
Sardh	2.05E-31	0.33696885	2
F10	5.71E-31	0.312933084	2
Atf5	9.38E-31	0.368449906	2
Apoc2	1.05E-30	0.375408918	2
Orm1	1.29E-30	0.31114798	2
Tkfc	1.92E-30	0.365862833	2
Cbs	3.43E-30	0.315446519	2
Sco2	5.03E-30	0.307053859	2
Hc	5.66E-30	0.280911084	2
Tstd1	5.75E-30	0.37518184	2
Bdh2	1.15E-29	0.346658993	2
Ctsc	1.91E-29	0.410988608	2
Ttc36	3.87E-29	0.279966934	2
Slc27a5	1.06E-28	0.301992989	2
Ugt2b38	1.19E-28	0.391712673	2
Gnmt	1.57E-28	0.251836652	2
Sema4g	1.73E-28	0.342469412	2
Haao	3.12E-28	0.296034322	2

Glyctk	5.98E-28	0.358076598	2
Hnf4a	2.23E-27	0.337507715	2
Tat	2.27E-27	0.301584579	2
Abhd2	9.92E-27	0.385362508	2
Qdpr	1.53E-26	0.309422885	2
Serpina12	1.61E-26	0.519001478	2
Vtn	2.91E-26	0.264581386	2
Nr2f6	1.05E-25	0.345673813	2
Igf1	1.09E-25	0.275439459	2
Insig1	2.84E-25	0.326780895	2
Fgb	4.32E-25	0.223530435	2
Bhmt2	2.12E-24	0.295677289	2
Lgals9	1.33E-23	0.279640284	2
Arsg	1.35E-23	0.334539375	2
Hsd11b1	2.73E-23	0.278943844	2
Cebpa	3.11E-23	0.279540181	2
Ak4	4.28E-23	0.308983331	2
Pcyt2	6.14E-23	0.323444714	2
Slc7a2	6.15E-23	0.320817864	2
Sptbn2	9.41E-23	0.336358614	2
Suclg1	1.14E-22	0.319682795	2
Osgin1	1.33E-22	0.362253569	2
Wfdc21	1.74E-22	0.260611385	2
Cyc1	2.19E-22	0.267704106	2
Eppk1	2.39E-22	0.345567525	2
Cyp2u1	2.68E-22	0.354872615	2
Hgd	5.64E-22	0.266087421	2
Ccl9	6.96E-22	0.322712416	2
Pygl	8.90E-22	0.28945404	2
Ethe1	2.27E-21	0.320358734	2
Pnpla7	5.40E-21	0.269985633	2
Mup3	7.03E-21	0.283346864	2
Serpina1b	1.36E-20	0.218677001	2
Iigp1	1.89E-20	0.385693809	2
Plxna2	2.09E-20	0.279021972	2
Atp5g3	2.10E-20	0.243536221	2
Serpina1d	2.31E-20	0.21855102	2
Got2	2.36E-20	0.259397173	2
Cib3	4.85E-20	0.253912764	2
Fga	5.35E-20	0.206549755	2
Kyat3	5.35E-20	0.264268706	2
Abcb11	8.99E-20	0.275917404	2
Col18a1	9.95E-20	0.290981454	2
Slc25a22	9.99E-20	0.30832628	2
Sqor	1.18E-19	0.290946804	2
Fads2	2.41E-19	0.293117084	2
Khk	3.65E-19	0.237545375	2
Ddah1	3.88E-19	0.327763403	2
Ctnnbip1	4.53E-19	0.287305954	2
Serpinf2	4.79E-19	0.213562988	2
Ndufv1	6.63E-19	0.283608774	2
Hgfac	7.40E-19	0.276389666	2
Creb3l3	8.11E-19	0.252937566	2
Tm4sf4	3.02E-18	0.305789152	2
Tff3	3.49E-18	0.771459535	2
Tmprss6	3.59E-18	0.259939564	2
Lsr	4.03E-18	0.292196509	2
Cluh	6.42E-18	0.25805664	2
Gm2a	8.21E-18	0.250196428	2

Aqp8	8.27E-18	0.303231461	2
Aldh7a1	1.08E-17	0.256037645	2
Cmah	1.57E-17	0.282699314	2
Tiam2	2.61E-17	0.265699595	2
Clec2h	2.71E-17	0.297047641	2
Prpf8	7.42E-17	0.290981454	2
Ulk1	9.21E-17	0.292369656	2
Foxa3	1.26E-16	0.286340438	2
C8g	1.49E-16	0.231531008	2
Pglyrp2	1.61E-16	0.278046759	2
Maob	1.85E-16	0.268305993	2
Angptl8	2.14E-16	0.277454609	2
Acad11	2.84E-16	0.284425578	2
Serpina1e	2.85E-16	0.22775827	2
Larp1b	2.86E-16	0.282929621	2
Hsd3b3	5.06E-16	0.24685544	2
Oaf	5.70E-16	0.261865022	2
Gstz1	5.81E-16	0.240861644	2
Tars	6.30E-16	0.280870237	2
Kyat1	7.47E-16	0.252396019	2
Clpx	7.82E-16	0.275154975	2
Gm33543	1.28E-15	0.260547411	2
Lpin1	1.33E-15	0.419384501	2
Hoga1	1.43E-15	0.257283256	2
Hectd1	1.66E-15	0.260767379	2
Gchfr	2.85E-15	0.242356898	2
Gpt	3.10E-15	0.286441465	2
Foxq1	3.11E-15	0.501090574	2
Hmgcs2	5.05E-15	0.20920871	2
Sult5a1	5.62E-15	0.275423199	2
Rxra	6.36E-15	0.243546058	2
Ambp	6.67E-15	0.170370695	2
Itih2	6.85E-15	0.222782462	2
Ly6e	8.56E-15	0.252057717	2
Glo1	1.11E-14	0.257358483	2
Mdh1	1.20E-14	0.200549495	2
Ndrg2	1.29E-14	0.203404623	2
Paxx	1.33E-14	0.252780409	2
Serpina1c	1.35E-14	0.236189571	2
Slco2a1	1.82E-14	0.264805524	2
Gspt1	2.79E-14	0.273085356	2
Cfb	3.47E-14	0.217853379	2
Camk2n1	3.66E-14	0.261731183	2
Kmo	4.56E-14	0.25860887	2
Sucla2	5.28E-14	0.257845922	2
Cpb2	6.78E-14	0.195749088	2
Apon	7.14E-14	0.209716653	2
Gpt2	7.16E-14	0.280676428	2
Apof	7.57E-14	0.201419671	2
Aldh8a1	8.88E-14	0.242018982	2
Calr	9.71E-14	0.219997997	2
Baat	1.44E-13	0.214821621	2
H6pd	1.67E-13	0.23362186	2
Fgg	1.73E-13	0.179374291	2
Pklr	1.95E-13	0.312568774	2
Atp1b1	2.40E-13	0.265234319	2
Zfp750	2.50E-13	0.210421711	2
G6pc	2.77E-13	0.289700436	2
Pcx	3.03E-13	0.219251847	2

St6gal1	3.31E-13	0.257841436	2
Hsd17b2	4.01E-13	0.255099178	2
Slc37a4	4.62E-13	0.289348274	2
Hagh	4.71E-13	0.233813039	2
Lap3	5.09E-13	0.204797957	2
Slc38a4	5.74E-13	0.18671608	2
Elovl2	7.95E-13	0.236959194	2
Tmem25	8.03E-13	0.279630211	2
Ppargc1b	9.65E-13	0.262223119	2
Cyp4v3	1.36E-12	0.216171678	2
Nr1h3	1.75E-12	0.260378936	2
St3gal5	1.92E-12	0.249312984	2
Ppa1	1.99E-12	0.217427783	2
Ephx2	2.01E-12	0.202700199	2
Hbb-bt	2.17E-12	0.464476689	2
Myh9	2.30E-12	0.25355045	2
Pgrmc2	2.45E-12	0.252578269	2
Fmo5	2.56E-12	0.216398138	2
Chka	3.03E-12	0.271712983	2
Sdha	3.47E-12	0.213869514	2
Errfi1	3.87E-12	0.303309292	2
Uqcrfs1	3.97E-12	0.202168378	2
Dmgdh	5.25E-12	0.225407037	2
Plg	5.44E-12	0.173148514	2
Tfr2	7.44E-12	0.230313236	2
Gss	8.26E-12	0.255417928	2
Afm	8.36E-12	0.196622138	2
Abca6	8.70E-12	0.225215799	2
Sec31a	9.03E-12	0.254355547	2
Cryl1	9.25E-12	0.245803528	2
Pink1	1.15E-11	0.234813058	2
Cpt1a	1.47E-11	0.223206994	2
Acacb	1.56E-11	0.233000462	2
Acat3	1.57E-11	0.23596223	2
H2-K1	1.78E-11	0.23789327	2
Atp5g1	1.92E-11	0.258134777	2
Elovl5	1.99E-11	0.221906424	2
Os9	2.13E-11	0.277150831	2
Fabp1	2.52E-11	0.189667338	2
1300017J02Rik	4.44E-11	0.178594177	2
Fads1	5.02E-11	0.224395107	2
Ak2	7.21E-11	0.196341796	2
Ak3	7.64E-11	0.23612351	2
AI182371	7.92E-11	0.229311081	2
Tomm40l	8.12E-11	0.235273825	2
Actb	9.08E-11	0.20782331	2
Lrp1	9.85E-11	0.241376724	2
Cyb5r3	9.91E-11	0.189338216	2
Pdcd4	1.12E-10	0.204364348	2
Acads	1.13E-10	0.224390412	2
Mfsd2a	1.18E-10	0.202108393	2
Keg1	1.34E-10	0.207644987	2
Comt	1.40E-10	0.192932745	2
Mmp15	2.06E-10	0.247227222	2
Shfl	2.28E-10	0.254361583	2
F12	2.73E-10	0.189895965	2
Trp53inp2	3.31E-10	0.23799454	2
Alkbh5	3.41E-10	0.248875609	2
Ppif	3.94E-10	0.224880879	2

Aadac	4.54E-10	0.188701727	2
Ndufs2	5.05E-10	0.208772507	2
Mat1a	5.21E-10	0.164276474	2
Anxa6	5.44E-10	0.218142178	2
Mbl2	5.63E-10	0.208276661	2
Mccc2	5.73E-10	0.216573025	2
Esrra	6.42E-10	0.205972788	2
Serpina3k	7.09E-10	0.170170263	2
Rnf125	7.57E-10	0.215858788	2
Slc39a14	1.16E-09	0.241046504	2
Sqle	1.18E-09	0.247818737	2
Cyp17a1	1.56E-09	0.183764822	2
Bhlhe40	1.57E-09	0.253754197	2
Gcdh	1.59E-09	0.186340354	2
Pecr	1.71E-09	0.215759367	2
Gclc	2.08E-09	0.214564568	2
Tex2	2.10E-09	0.19885544	2
Sec16a	2.11E-09	0.219051416	2
Ndufs1	2.22E-09	0.216488291	2
Cfi	2.38E-09	0.23495597	2
Oplah	2.53E-09	0.216433173	2
Myo18a	2.55E-09	0.22431857	2
Evi5	2.67E-09	0.238975803	2
Coq8a	3.32E-09	0.189612254	2
Gatd3a	3.39E-09	0.218606017	2
Frmd4b	3.40E-09	0.24083181	2
Sdc1	4.10E-09	0.204373261	2
Abca1	4.77E-09	0.214277469	2
Slc25a1	6.04E-09	0.219322797	2
Gcat	6.49E-09	0.213486935	2
Hnrnpl	6.66E-09	0.224063279	2
Aldh4a1	6.98E-09	0.221483913	2
Isoc2a	7.10E-09	0.240813827	2
Slc25a15	7.17E-09	0.215978329	2
Nbr1	8.14E-09	0.221535101	2
Gclm	8.79E-09	0.247699813	2
Uqcrc1	9.03E-09	0.203779653	2
Whamm	9.05E-09	0.238208015	2
Bhmt	9.22E-09	0.216990193	2
Rbbp4	9.41E-09	0.207123954	2
Lgals8	9.56E-09	0.215904558	2
Helz2	1.01E-08	0.200415156	2
Echs1	1.07E-08	0.187466142	2
C2	1.10E-08	0.225763572	2
Gamt	1.36E-08	0.190817282	2
Acy3	1.41E-08	0.22700222	2
Edem1	1.48E-08	0.182086041	2
Kif21a	1.61E-08	0.227875244	2
B4galnt1	1.71E-08	0.221822585	2
Etnk1	1.73E-08	0.209943033	2
Slc17a2	2.04E-08	0.198476087	2
Hnrnpu	2.22E-08	0.216752337	2
Gm4788	2.22E-08	0.183304997	2
Pgam1	2.47E-08	0.207461699	2
Cyp39a1	2.57E-08	0.238230738	2
Cox5b	2.59E-08	0.173868723	2
Crot	3.21E-08	0.162680949	2
Prox1	3.29E-08	0.19536425	2
Pdk2	3.65E-08	0.210344836	2

Dpyd	3.76E-08	0.217255947	2
Afmid	4.45E-08	0.197010076	2
Acmsd	4.54E-08	0.223186316	2
Acadvl	4.80E-08	0.188169543	2
Usp4	4.88E-08	0.224070094	2
Ugp2	5.01E-08	0.181860302	2
Xbp1	5.05E-08	0.243306457	2
Fh1	5.08E-08	0.1826229	2
Ddx3x	5.62E-08	0.201505791	2
Larp4b	5.83E-08	0.234064473	2
Plxnb2	5.85E-08	0.197954386	2
Pdia5	6.18E-08	0.239846477	2
Tob1	6.22E-08	0.221948763	2
Slc25a25	6.64E-08	0.251791527	2
Epn1	7.67E-08	0.227847569	2
Snrnp70	7.83E-08	0.194452333	2
Irf2bp2	8.98E-08	0.234505701	2
Jak1	9.29E-08	0.224058364	2
Sqstm1	9.71E-08	0.175436562	2
Cdk9	1.18E-07	0.225797179	2
Rrbp1	1.23E-07	0.19049805	2
Shroom1	1.26E-07	0.224329304	2
Masp2	1.42E-07	0.175818379	2
Arsa	1.43E-07	0.167937246	2
Cfh	1.46E-07	0.160092558	2
Ebp	1.77E-07	0.178261838	2
Selenbp1	1.83E-07	0.206172115	2
Ctsz	1.97E-07	0.185741404	2
Jund	1.98E-07	0.183408592	2
Ifi27	2.07E-07	0.213608675	2
Zbtb20	2.11E-07	0.195131902	2
Ero1lb	2.15E-07	0.228505006	2
Ndst1	2.25E-07	0.210513868	2
Proser2	2.25E-07	0.191753251	2
Stra6l	2.31E-07	0.200341374	2
Stard7	2.35E-07	0.204046362	2
Serpina10	2.39E-07	0.189648354	2
2810459M11Rik	2.46E-07	0.217343289	2
Snd1	2.70E-07	0.205309015	2
Insig2	2.78E-07	0.185324495	2
Vegfa	2.83E-07	0.209431855	2
Ptprf	3.19E-07	0.228733205	2
Klf15	3.28E-07	0.215762217	2
Retreg2	3.32E-07	0.200071578	2
Nr1d1	3.84E-07	0.226808838	2
Zfp598	3.98E-07	0.223780936	2
Atp1a1	4.01E-07	0.187617373	2
Phyh	4.17E-07	0.153181963	2
Ogdh	4.67E-07	0.192843467	2
Nab2	5.04E-07	0.202283716	2
Kynu	5.41E-07	0.188596875	2
Ap1g1	5.57E-07	0.203478149	2
Pi4k2a	5.74E-07	0.196697522	2
Slc27a2	5.99E-07	0.147985539	2
Gjb1	6.32E-07	0.158147203	2
Rhobtb1	7.43E-07	0.205162703	2
Slc25a5	7.79E-07	0.181407412	2
B4galt5	7.79E-07	0.214108895	2
Dyrk2	7.79E-07	0.19872116	2

Echdc2	7.81E-07	0.165239067	2
S100a1	8.08E-07	0.173283781	2
Pank1	8.19E-07	0.187452889	2
Srrm2	8.57E-07	0.180271267	2
Cat	8.92E-07	0.143300955	2
Furin	1.00E-06	0.192759795	2
Spata2l	1.08E-06	0.189574895	2
Srd5a1	1.13E-06	0.204066864	2
Fbxo9	1.18E-06	0.213083145	2
Dcaf11	1.21E-06	0.181800665	2
Azgp1	1.22E-06	0.146700994	2
Prpsap1	1.23E-06	0.205335044	2
Slc30a10	1.24E-06	0.190866774	2
Apeh	1.32E-06	0.207376459	2
Adck5	1.49E-06	0.185208042	2
Nipsnap1	1.61E-06	0.178895607	2
Igfbp4	1.65E-06	0.152801359	2
Esr1	1.69E-06	0.223940835	2
Eif4g1	1.70E-06	0.177923531	2
Tcf7l1	1.72E-06	0.170182314	2
Habp4	1.74E-06	0.19558096	2
Safb2	1.92E-06	0.202867084	2
Kalrn	1.95E-06	0.219328913	2
Acaca	2.11E-06	0.21684487	2
Itih4	2.21E-06	0.131359173	2
Pgpep1	2.30E-06	0.198878423	2
Ranbp10	2.91E-06	0.210310765	2
Akr1c19	2.94E-06	0.225800757	2
Gpx1	2.95E-06	0.155905197	2
Slc2a2	2.96E-06	0.224419129	2
Itih1	2.97E-06	0.183822385	2
Trip12	3.04E-06	0.206498284	2
Psen2	3.06E-06	0.170523574	2
Eif4b	3.08E-06	0.178682984	2
Suds3	3.10E-06	0.202839281	2
Esrp2	3.32E-06	0.202624417	2
Hnrnpab	3.63E-06	0.175757847	2
Micos13	3.65E-06	0.179638329	2
Dclk3	3.80E-06	0.147520972	2
Sdhd	3.87E-06	0.203629326	2
Csf1r	3.94E-06	0.210753223	2
Akap11	4.15E-06	0.202612446	2
Gsap	4.18E-06	0.184197752	2
Sec24a	4.26E-06	0.185236967	2
Mgam	4.37E-06	0.181048286	2
Ptms	4.68E-06	0.150097169	2
Aco1	4.80E-06	0.187707436	2
Itih5	5.00E-06	0.16284512	2
Sdhb	5.39E-06	0.154791937	2
Usp9x	5.54E-06	0.185305879	2
Srsf2	5.91E-06	0.202907982	2
Mtss1	6.42E-06	0.215353587	2
Prpf6	6.81E-06	0.183966714	2
Cxxc5	7.05E-06	0.191137422	2
Jade1	7.06E-06	0.186986402	2
Nipal1	7.09E-06	0.178404509	2
Abhd14b	8.38E-06	0.197021351	2
Cecr2	9.02E-06	0.159386038	2
Irf5	9.71E-06	0.191375412	2

Adk	9.84E-06	0.152138184	2
Cry1	9.97E-06	0.154069415	2
Klhl24	1.02E-05	0.183837782	2
Zkscan1	1.09E-05	0.213631854	2
Serpind1	1.18E-05	0.152785746	2
Rnf103	1.20E-05	0.207543202	2
Slc25a51	1.27E-05	0.167648873	2
Prodh2	1.33E-05	0.193058124	2
2900026A02Rik	1.40E-05	0.196275336	2
Serping1	1.41E-05	0.164101743	2
Garem1	1.45E-05	0.169275063	2
Grb7	1.45E-05	0.200135072	2
St3gal3	1.56E-05	0.183199325	2
Hadha	1.59E-05	0.177477843	2
Ddi2	1.82E-05	0.17203968	2
Hspa9	1.83E-05	0.160835645	2
Ndufa10	2.01E-05	0.171754023	2
Ptpn3	2.08E-05	0.182922998	2
Iah1	2.13E-05	0.177349479	2
Ces3b	2.16E-05	0.189903069	2
Anks4b	2.19E-05	0.202435347	2
Cobl	2.24E-05	0.177282401	2
Gch1	2.26E-05	0.177176996	2
Sympk	2.26E-05	0.187559336	2
Slc25a39	2.26E-05	0.151355055	2
Plxnb1	2.28E-05	0.180567592	2
Amfr	2.31E-05	0.1819567	2
Hpn	2.68E-05	0.144158462	2
Itch	2.72E-05	0.169501954	2
Il1rap	2.87E-05	0.164605467	2
Hnmt	3.03E-05	0.172389044	2
Gsto1	3.15E-05	0.183262973	2
Nlrp6	3.17E-05	0.193382728	2
Coq9	3.33E-05	0.18720588	2
Adh4	3.34E-05	0.177052416	2
Tnfaip2	3.45E-05	0.203867836	2
Fxyd1	3.67E-05	0.144294781	2
Pcca	3.72E-05	0.190127652	2
Sult1a1	3.80E-05	0.14954446	2
Myo1e	3.84E-05	0.188797192	2
Apom	3.87E-05	0.139029758	2
F7	4.10E-05	0.191041988	2
Arhgef18	4.29E-05	0.144917254	2
Trap1	4.32E-05	0.179037612	2
Dpp3	4.39E-05	0.158762607	2
Rarres2	4.41E-05	0.168560086	2
Gatad2a	4.50E-05	0.183541773	2
Klf9	4.55E-05	0.201400409	2
Nnt	4.74E-05	0.141006163	2
Egln1	4.75E-05	0.150546652	2
Rdh7	4.99E-05	0.137620706	2
Spryd4	5.15E-05	0.183785288	2
Ndufv3	5.17E-05	0.170721075	2
Scrn2	5.26E-05	0.168899266	2
Ppp6r1	5.31E-05	0.186646729	2
Egfr	5.36E-05	0.140785736	2
Eif3a	5.68E-05	0.174879441	2
Pnpla2	6.14E-05	0.162600435	2
Ppfibp1	6.55E-05	0.147100484	2

Nsun2	6.85E-05	0.175300517	2
Tnpo2	6.86E-05	0.137960684	2
St3gal1	7.03E-05	0.188976363	2
Gltpd2	7.33E-05	0.187729501	2
Eva1a	7.39E-05	0.191065053	2
Khnyn	7.84E-05	0.177713686	2
Srp68	8.00E-05	0.160801419	2
Slc41a2	8.11E-05	0.132446603	2
Mogs	8.29E-05	0.180664666	2
Wdr26	8.35E-05	0.183240265	2
Supt5	8.63E-05	0.177921132	2
Mn1	8.77E-05	0.141937004	2
Med25	0.000102047	0.178869141	2
Thrsp	0.000102189	0.244432052	2
Gpd1	0.000104872	0.158434303	2
Fggy	0.000108333	0.181031987	2
Etnk2	0.000114547	0.162299698	2
Csk	0.000114763	0.164223805	2
Wdtc1	0.000114845	0.175422964	2
Dnajc22	0.000116597	0.160858214	2
Sec61a1	0.000121134	0.174239713	2
Epas1	0.00012417	0.159191543	2
Fbxo31	0.000125376	0.17172941	2
Cald1	0.000126364	0.146323574	2
Ido2	0.000137427	0.173377554	2
Hmgcr	0.000139013	0.152756295	2
Adra1b	0.00014367	0.152273398	2
Kcnk5	0.000143896	0.170173874	2
Ndufb6	0.000147254	0.158070805	2
Tbrg4	0.000157396	0.147940128	2
Arf1	0.000180656	0.188168402	2
Hdgf	0.000203768	0.155668331	2
F11r	0.000205352	0.176494689	2
Aamp	0.000207666	0.168011244	2
Eif4h	0.000233977	0.168580029	2
Atxn1	0.000235302	0.173524388	2
Cdk5rap3	0.000245473	0.196724634	2
Preb	0.000248865	0.181798217	2
Epb41	0.000250658	0.167315363	2
Ctdsp1	0.000257943	0.164371939	2
Uqcr10	0.000264874	0.146655396	2
Slc30a9	0.000282019	0.170782771	2
Fgfr2	0.000288507	0.201329421	2
Akap8	0.000296357	0.143867773	2
Arid1a	0.000296769	0.159548161	2
Lurap1l	0.000298192	0.166044462	2
Acp1	0.000316363	0.170141749	2
Dpys	0.000332898	0.164333245	2
Lss	0.000356953	0.168625669	2
Supt6	0.000361718	0.160024828	2
Apoa5	0.000376723	0.150476585	2
Scap	0.000381206	0.190836221	2
Crebzf	0.000384255	0.177917595	2
Bpgm	0.000385003	0.13399144	2
Nudt7	0.000400629	0.152147456	2
Hspa5	0.000413017	0.144665882	2
Txndc5	0.000433021	0.170148939	2
Spata13	0.000455255	0.168769832	2
Atp7b	0.00046184	0.137670629	2

Brap	0.000495322	0.153495817	2
Bag4	0.000530082	0.149089923	2
Gck	0.00055383	0.183732847	2
Gcgr	0.000556024	0.137839806	2
Cxadr	0.000562009	0.162225359	2
Pex6	0.000574038	0.171003903	2
Arhgap35	0.000589326	0.17071657	2
Ctnna1	0.000600062	0.163308607	2
Pex5	0.000605541	0.157874415	2
Huwe1	0.000610604	0.166430015	2
Serpinf1	0.000636249	0.138161737	2
Pgk1	0.000646797	0.18209649	2
Foxp4	0.000656009	0.12801768	2
Agpat3	0.000663036	0.157581308	2
Pla2g12b	0.000684041	0.16665911	2
Prrc2a	0.000684119	0.163264916	2
Sacm1l	0.000761456	0.163249905	2
Sdr9c7	0.000766234	0.187849052	2
Asph	0.000794852	0.15268361	2
Ndufa5	0.000845272	0.152040783	2
Apoa4	0.000867369	0.11119414	2
Zdhhc9	0.000869282	0.154579995	2
Cpn2	0.00087514	0.142837411	2
Manf	0.000903088	0.184108816	2
Sh3bp2	0.000947134	0.124425265	2
Nedd4l	0.000955701	0.164139865	2
Cdip1	0.000968955	0.210528757	2
Ctsb	0.00102552	0.118818357	2
Mtor	0.001152342	0.173601599	2
Slc8b1	0.001168593	0.168010466	2
Opa1	0.001179378	0.174717117	2
Atl2	0.001194709	0.164935714	2
Dld	0.001233399	0.154873496	2
Psme2b	0.001278183	0.151450558	2
Wdr81	0.001320846	0.16673269	2
Gak	0.001324927	0.164515453	2
Spen	0.001336504	0.125087224	2
Reep6	0.001375523	0.148293137	2
Uqcrc2	0.001396293	0.151785219	2
Hyou1	0.001412377	0.166032007	2
Scfd1	0.001421248	0.164472797	2
Afg3l2	0.001461722	0.159812762	2
Tcea3	0.001525705	0.151074219	2
Clu	0.00152573	0.117435327	2
Slc25a11	0.001572222	0.156625623	2
Mid1ip1	0.001641791	0.169619681	2
Fam160b1	0.001676937	0.146419771	2
Mthfd1	0.001767665	0.146764026	2
Gns	0.001819369	0.180970918	2
Abat	0.001847279	0.172213974	2
Ctsl	0.001861031	0.128248374	2
Fpgs	0.001891346	0.187224156	2
Atp5k	0.001893247	0.139354422	2
Arhgef12	0.001932261	0.156943493	2
Itprid2	0.002228818	0.157332876	2
Sec24c	0.002263622	0.158465949	2
Spr	0.00226407	0.157101605	2
Fn1	0.002332173	0.123186135	2
Cul4a	0.002332199	0.157342778	2

Fam47e	0.002387397	0.13738001	2
Ccnf	0.002421081	0.166020496	2
Sod2	0.002443114	0.167559166	2
Lrpprc	0.002468256	0.163914884	2
Hykk	0.002470332	0.178520946	2
Myrf	0.002471441	0.149322142	2
Samd8	0.002732172	0.158233075	2
C8b	0.002788321	0.134183919	2
Safb	0.002824042	0.133831377	2
Slc35c2	0.002843643	0.147492541	2
Hdlbp	0.002890352	0.143063374	2
Egln2	0.002909366	0.160285559	2
Dhx33	0.002983384	0.105568301	2
Hspa4	0.003173732	0.151874664	2
Igfbp2	0.003184482	0.131800886	2
Copz1	0.003205118	0.137175363	2
Apol9b	0.003250031	0.196003092	2
Gigyf1	0.003286694	0.145014657	2
Txndc15	0.003341047	0.155956626	2
Lonp1	0.003389209	0.168756254	2
Gucd1	0.003554943	0.161333134	2
Uqcrh	0.00360099	0.145860847	2
Tedc2	0.003618281	0.213206745	2
Ndufs5	0.003649563	0.13169451	2
Plekhg6	0.003904647	0.140378613	2
Otub1	0.003925427	0.168358221	2
Echdc3	0.004013692	0.163149567	2
Tsc22d4	0.004049732	0.131678388	2
P2rx4	0.004051016	0.156340956	2
Sdr42e1	0.004342241	0.175303508	2
Ubr5	0.00434538	0.175170341	2
Atg2a	0.004380408	0.132643635	2
Laptm4a	0.004421572	0.123957318	2
Vps11	0.004537995	0.156290383	2
Mia2	0.004562035	0.136365794	2
Aspdh	0.004594248	0.16687014	2
Etfb	0.004606053	0.137795001	2
Sfmbt1	0.004840328	0.125501222	2
Dazap1	0.004860888	0.154707605	2
Tubb2a	0.005136833	0.161298086	2
Hint1	0.005179094	0.158743928	2
Atp13a1	0.00549474	0.1514093	2
Slc9a3r1	0.005521628	0.15120405	2
Mta2	0.005525987	0.148081646	2
Slc40a1	0.005546063	0.17802834	2
Atg13	0.005608056	0.15782281	2
Cp	0.005668121	0.116323742	2
Fgfr4	0.005710878	0.128878139	2
Carhsp1	0.005729038	0.157889111	2
Rai14	0.00585674	0.136264062	2
Ccser2	0.005889109	0.137542043	2
Yy1	0.006132249	0.142166064	2
Kng2	0.006509064	0.140198232	2
Antxr2	0.006580505	0.147422036	2
Samm50	0.006611665	0.146875913	2
AU022252	0.006685698	0.158231294	2
Txndc11	0.006859896	0.157192139	2
Wdr1	0.006907602	0.133353738	2
Mtmr3	0.007154779	0.172042387	2

Zbtb16	0.00719793	0.145883269	2
Mafb	0.007215666	0.188149941	2
Nrbp2	0.007240073	0.151378133	2
Litaf	0.00739245	0.156488917	2
Pter	0.007543432	0.147819606	2
Hectd3	0.007590014	0.147905701	2
Isoc1	0.007824513	0.155746875	2
Ier2	0.007842276	0.117408623	2
Slc6a13	0.007908745	0.153887877	2
Dennd5b	0.008095788	0.131197043	2
Hes6	0.008253469	0.171902086	2
C130074G19Rik	0.008446614	0.125753389	2
Hadh	0.008458546	0.155024984	2
Foxo4	0.008515343	0.147151427	2
Mul1	0.008681899	0.162493874	2
Ghitm	0.008800537	0.137919675	2
Mlf2	0.008839152	0.127599705	2
Cyp2j5	0.008904847	0.121653026	2
Uso1	0.009335865	0.159848492	2
Ide	0.009624857	0.129179726	2
Hspd1	0.009749122	0.120423577	2
Asb13	0.009942856	0.16128491	2
Dhcr24	0.010373095	0.156128111	2
Pex14	0.010550868	0.163247345	2
Gprc5c	0.01064854	0.156401124	2
Fat1	0.010701119	0.152585277	2
Fam20c	0.010712196	0.157537475	2
Sox9	0.011001496	0.121887819	2
Tcf25	0.011064774	0.161376347	2
Plbd2	0.011549431	0.180170684	2
Pde4dip	0.011607826	0.136053253	2
Mst1	0.012541977	0.150610326	2
Cfhr2	0.012712744	0.106438097	2
Trpc4ap	0.012740515	0.150033962	2
Epb41l5	0.013011663	0.131322738	2
Kif1c	0.013020082	0.142436744	2
Tmbim6	0.013639856	0.116280085	2
Nsmf	0.013998425	0.145299205	2
Upb1	0.014246789	0.12627391	2
Slc23a1	0.014432574	0.151276955	2
Rmnd5a	0.014522189	0.151959547	2
Pbld2	0.014790343	0.154166203	2
Marco	0.015074024	0.182527402	2
Scd1	0.015349919	0.196269529	2
Gm16286	0.015494864	0.143728746	2
Dazap2	0.015726866	0.153210969	2
Ndufa1	0.015814252	0.131584299	2
Hes1	0.015959647	0.147812226	2
Fyco1	0.015967912	0.127931757	2
Syne1	0.016178557	0.126823911	2
Zfp385a	0.016358483	0.111497715	2
Scnn1a	0.017028134	0.172636703	2
Retreg1	0.017200033	0.161885165	2
Ndufa6	0.017838156	0.140055479	2
Bag6	0.017848846	0.140503415	2
Tmem260	0.018054706	0.135026347	2
Ndufab1	0.018393179	0.148461781	2
Mindy2	0.019237301	0.130163877	2
Lars	0.019279549	0.155059408	2

Ddb1	0.019949902	0.153059596	2
Enpp2	0.020241012	0.143502667	2
Ubc	0.020250996	0.127331415	2
Hsd3b2	0.020529333	0.140382754	2
Rreb1	0.020596327	0.151464832	2
Strn3	0.020649107	0.127015706	2
Abtb2	0.02156286	0.130502204	2
Usp47	0.022042626	0.142342163	2
Zhx3	0.02234703	0.155204	2
Eif2ak1	0.0227709	0.148693943	2
Acadsb	0.023505117	0.160772703	2
Gpr146	0.023934723	0.152311797	2
Entpd8	0.024189722	0.148277764	2
Cadm1	0.024464972	0.131621592	2
Zfp655	0.024585968	0.144874305	2
Ccs	0.025324306	0.131543485	2
Tnks2	0.025796221	0.121635045	2
Mmut	0.026671113	0.141700903	2
Nadsyn1	0.026714722	0.144873318	2
Mocs3	0.026740305	0.105146295	2
Dhx38	0.027099003	0.135030364	2
Alas2	0.027460896	0.177379512	2
Sel1l	0.027691682	0.143246102	2
Timm44	0.027858386	0.120234468	2
Tnk2	0.028136966	0.13594878	2
Glud1	0.028372049	0.099557265	2
Prxl2a	0.028419025	0.162542243	2
Atp5j	0.028913653	0.113650712	2
Mief1	0.030484592	0.116174856	2
Tmem86b	0.030769624	0.150742228	2
Inppl1	0.030893	0.126535566	2
Slc20a1	0.030934244	0.153367991	2
Ndufc2	0.031753729	0.136419803	2
Retsat	0.031830889	0.197112209	2
Osbp	0.031867254	0.111868321	2
Rpusd3	0.032012286	0.129705592	2
Lasp1	0.032490759	0.158746833	2
Nr1i2	0.032678586	0.144084202	2
Tm6sf2	0.033511531	0.135080633	2
Baiap2	0.033531796	0.172413267	2
Gm4951	0.033974242	0.168838513	2
Sppl2a	0.034844769	0.147181942	2
Rnf187	0.03560244	0.146024725	2
Prrc2b	0.038140985	0.116408089	2
Myo10	0.03840153	0.141309858	2
Usp19	0.038700281	0.123490058	2
Ivd	0.03904066	0.145060329	2
Trabd	0.039581134	0.16459481	2
Srpr	0.039618286	0.142372617	2
Heca	0.039703036	0.142553829	2
Twnk	0.04036379	0.110213864	2
Wsb1	0.040746565	0.144980131	2
Zcchc24	0.04171201	0.149021848	2
H1f0	0.041926076	0.14806299	2
Bcl3	0.042100051	0.144349126	2
Parp12	0.042165941	0.145248665	2
Acnat1	0.044290734	0.13413719	2
Nampt	0.045078858	0.172969134	2
Bcr	0.045391408	0.13184153	2

Rassf3	0.046909568	0.13939315	2
Stard5	0.047259977	0.135789938	2
Ndufb9	0.047382999	0.132813911	2
Ppib	0.048747994	0.114584863	2
2310061I04Rik	0.04891172	0.137972646	2
Apoc3	0.048983007	0.106356933	2
Larp1	0.049191705	0.143079253	2
Adap2	0.049424969	0.11256159	2
Mup11	7.99E-60	0.723069194	3
Cyp2c29	2.44E-65	0.566223374	3
Cyp2e1	1.19E-50	0.561624037	3
Pon1	3.25E-49	0.422979289	3
Rnase4	7.64E-38	0.366526121	3
Cyp1a2	1.29E-36	0.393452908	3
Gulo	6.83E-36	0.320274777	3
Rgn	3.68E-34	0.310638205	3
Slco1b2	1.30E-32	0.388297601	3
Cyp2c54	2.70E-31	0.469320687	3
Oat	1.08E-29	0.115041583	3
Ang	2.14E-29	0.364800141	3
Nr1i3	6.39E-26	0.363528085	3
Aldh3a2	1.32E-22	0.238069672	3
Lect2	3.94E-21	0.291239757	3
Lifr	8.12E-20	0.309775151	3
Ugt2b1	1.81E-19	0.278483523	3
Sult2a8	6.22E-19	0.266608842	3
Mup17	9.70E-19	0.468479741	3
Ecm1	1.47E-16	0.339978641	3
Cyp7a1	1.76E-16	0.279094643	3
Aqp9	2.23E-16	0.256084183	3
Cyp3a11	6.73E-15	0.217475077	3
Dcn	2.46E-13	0.297233275	3
Cyb5a	5.73E-13	0.179394547	3
Sult1d1	2.72E-12	0.28300735	3
Gsta3	3.07E-12	0.15759184	3
Abcc2	9.26E-12	0.256546626	3
Mup10	1.49E-11	0.291810594	3
Aldh1a1	1.64E-11	0.174258628	3
Cyp2c68	3.99E-11	0.24293334	3
Cyp2c70	5.77E-11	0.184349883	3
Aqp1	1.35E-10	0.254584661	3
Tpt1	1.53E-10	0.144255222	3
Fth1	2.09E-10	0.161768575	3
Cldn2	4.05E-09	0.230936536	3
Npr2	1.67E-08	0.185922953	3
Hamp2	4.60E-08	0.287747154	3
Cyp7b1	8.25E-08	0.209259908	3
Susd4	1.24E-07	0.193259835	3
Reln	2.03E-07	0.23445027	3
Tsku	2.84E-07	0.223564862	3
Eci2	3.64E-07	0.162280945	3
Cish	4.37E-07	0.246093216	3
Slc16a10	4.72E-07	0.200571928	3
Plpp1	5.45E-07	0.209762155	3
Por	6.56E-07	0.180009841	3
Saa4	7.14E-07	0.211117869	3
Cyp27a1	7.27E-07	0.148626787	3
Gadd45g	1.07E-06	0.241386214	3

Hamp	1.53E-06	0.262366589	3
Hgf	3.98E-06	0.198919687	3
Cd164	1.16E-05	0.172552347	3
Entpd5	3.94E-05	0.143109506	3
Apoa2	5.91E-05	0.112295273	3
Mgst1	7.12E-05	0.107026037	3
Adtrp	7.99E-05	0.196803944	3
Gm4756	9.20E-05	0.17354802	3
Dnase1l3	0.000104322	0.184259039	3
Clec4g	0.000166297	0.185959908	3
Cdo1	0.000171315	0.124768872	3
Cyp4a14	0.000175394	0.130375311	3
Akr1c20	0.000259401	0.142844314	3
Slco1a1	0.000269593	0.14932872	3
Ugt2b35	0.000282138	0.164065857	3
H2-Aa	0.000320363	0.110030867	3
Fcna	0.000325478	0.190670993	3
Prlr	0.000351785	0.192608139	3
Alas1	0.000418059	0.195049483	3
C6	0.00060989	0.101651138	3
Nfyb	0.000676129	0.161185165	3
Ugt1a1	0.000685491	0.127664935	3
Plpp3	0.001031716	0.147237301	3
Stab2	0.001239828	0.188087575	3
Akr1c14	0.001302962	0.139547353	3
Bmp2	0.001802449	0.184351607	3
Blvrb	0.002818985	0.114731081	3
Slc29a1	0.002834332	0.153145766	3
Chpt1	0.003052831	0.174850795	3
Ftl1-ps1	0.00400151	0.091288775	3
Lrat	0.004550206	0.145951756	3
Slc10a1	0.004779415	0.14647819	3
Ghr	0.006399737	0.114937226	3
Hsd3b7	0.006774018	0.119636005	3
Phlda1	0.007363319	0.2027183	3
Mrc1	0.011314896	0.164733063	3
Fcgr2b	0.012653167	0.170573507	3
Csad	0.013232579	0.049428247	3
Cdh2	0.016340395	0.152123446	3
Mup12	0.018264574	0.180291339	3
Kit	0.025211466	0.118125059	3
Tinagl1	0.025596264	0.157098314	3
Cd74	0.036781613	0.067089505	3
Serpina3m	0.04767395	0.108613599	3
Oat	2.48E-179	1.580576953	4
Gulo	1.55E-171	1.149333222	4
Slc1a2	1.45E-152	1.519674865	4
Cyp2c29	2.38E-119	0.904760945	4
Cyp1a2	7.37E-117	0.981639741	4
Aldh3a2	2.09E-112	0.862249966	4
Rgn	8.03E-107	0.754365712	4
Cyp2e1	1.18E-100	0.897150123	4
Lect2	2.72E-97	0.928190957	4
Pon1	1.76E-93	0.656061271	4
Fitm1	6.19E-93	0.7650174	4
Slc22a1	4.46E-88	0.730291213	4
Slco1b2	5.90E-79	0.682094106	4
Rnase4	1.11E-78	0.612568828	4

Ces2c	3.49E-76	0.752441757	4
Serpina7	3.91E-74	0.736151908	4
C6	2.44E-71	0.615714436	4
Ang	6.02E-67	0.603981943	4
Tsc22d1	2.66E-64	0.627057686	4
Npr2	1.27E-56	0.649589062	4
Gstm3	1.17E-51	0.584015051	4
Cyp4a14	4.05E-51	0.680433762	4
Gstm2	1.55E-50	0.547487775	4
Lhpp	9.69E-49	0.460823619	4
Nr1i3	4.67E-46	0.523841285	4
Cyb5a	1.71E-45	0.411290428	4
Cyp3a11	3.06E-45	0.426443211	4
Cyp2c54	3.83E-44	0.621476646	4
Cyp7a1	1.07E-43	0.551131293	4
Fabp4	1.38E-42	0.603513421	4
Cyp4a10	1.88E-42	0.508310762	4
Gsta3	1.46E-41	0.385872836	4
Rspo3	1.01E-40	0.489889158	4
Slc13a3	2.38E-39	0.360585039	4
Aqp9	4.74E-39	0.462346726	4
Aldh1a1	2.99E-38	0.461974983	4
Akr1c20	8.74E-38	0.472503334	4
Por	1.98E-37	0.444021749	4
Nxpe2	2.34E-37	0.390568494	4
Cyp2a4	2.55E-37	0.409144172	4
Entpd5	2.73E-37	0.452605042	4
Mup11	1.15E-36	0.499300966	4
Fam25c	2.21E-36	0.467768001	4
Susd4	8.76E-35	0.434110414	4
Ugt2b1	1.20E-33	0.417912985	4
Bsg	7.91E-33	0.425600552	4
Blvrb	1.74E-31	0.448381798	4
Rdh16	6.77E-31	0.42941694	4
Slc16a10	3.22E-30	0.447801752	4
Eci2	9.60E-29	0.376035741	4
Abcc2	1.47E-28	0.388154875	4
Nrn1	5.93E-27	0.359968726	4
Elovl3	1.87E-26	0.397961005	4
Mup17	2.01E-26	0.696119951	4
Vwf	6.24E-25	0.410193443	4
Gstm1	9.76E-25	0.267728372	4
Ugt1a1	1.36E-24	0.352185757	4
Cyp2c55	1.70E-24	0.427048512	4
Acot1	2.40E-24	0.382817333	4
Wnt2	9.75E-24	0.237628056	4
Fmo1	6.57E-23	0.369348192	4
Ahr	1.51E-22	0.350881064	4
Tbx3	3.68E-22	0.307001967	4
Cldn2	8.65E-22	0.367438655	4
Tsku	8.47E-21	0.356441027	4
Csad	1.06E-20	0.292294255	4
Nt5e	1.48E-20	0.265562767	4
Axin2	1.52E-20	0.230745989	4
Slc43a3	1.98E-20	0.370903725	4
Serpina6	2.33E-20	0.334993663	4
Cyp27a1	2.49E-20	0.30102108	4
Pparg	1.84E-19	0.222161282	4
Ces2e	2.97E-19	0.336016177	4

Slc19a2	5.12E-19	0.328776144	4
Tmem243	9.64E-19	0.315914638	4
Plpp1	1.41E-18	0.382976778	4
Sult2a8	3.76E-18	0.274378464	4
Cyp2c68	4.25E-18	0.350020646	4
Fth1	9.01E-18	0.223452795	4
Tpt1	1.52E-17	0.222327233	4
Ftl1-ps1	2.30E-15	0.219261758	4
Cyp2c69	2.34E-15	0.307562931	4
Ugt2b34	2.66E-15	0.28370617	4
Lpl	3.17E-15	0.405790873	4
Acaa1b	6.94E-15	0.204364284	4
Chpt1	7.07E-15	0.334789286	4
Bgn	2.11E-14	0.283406564	4
Aqp1	2.46E-14	0.306426403	4
Ugt2b35	2.93E-14	0.328500226	4
Eng	6.55E-14	0.320406502	4
Kit	7.79E-14	0.256617439	4
Sod1	1.77E-13	0.203610561	4
Jam2	3.19E-13	0.293385533	4
Col3a1	3.27E-13	0.28083475	4
Mgst1	8.38E-13	0.205713219	4
Hsd17b12	1.64E-12	0.260241726	4
Fabp2	2.50E-12	0.278848874	4
Ube2e2	3.33E-12	0.283108281	4
Abcc9	4.85E-12	0.323230991	4
Cs	5.86E-12	0.260499257	4
Spp2	6.09E-12	0.259222201	4
C4bp	7.65E-12	0.214033826	4
Dcn	1.05E-11	0.316793282	4
Plpp3	4.16E-11	0.261860669	4
Adtrp	9.44E-11	0.263164947	4
Notum	1.40E-10	0.249899137	4
Slc16a7	1.87E-10	0.263502879	4
Mup10	2.78E-10	0.345586296	4
Hpd	3.52E-10	0.198348752	4
1810058I24Rik	4.12E-10	0.257819984	4
Cyp7b1	8.91E-10	0.242016328	4
Tinagl1	1.25E-09	0.273815166	4
Nfyb	1.64E-09	0.25568086	4
Ugt3a2	2.99E-09	0.224754361	4
Inhbe	3.08E-09	0.225959941	4
4931406C07Rik	5.78E-09	0.245320486	4
Rmdn2	6.32E-09	0.26743786	4
Fam107b	8.60E-09	0.222469733	4
Csrp3	8.75E-09	0.262275718	4
Plbd1	1.16E-08	0.234548029	4
Vnn1	2.54E-08	0.231569492	4
Reln	3.98E-08	0.249098498	4
Decr1	4.80E-08	0.197172408	4
Egln3	7.56E-08	0.2218894	4
Aldh2	8.46E-08	0.180341953	4
Adgrg3	9.21E-08	0.166166518	4
Arpp19	1.11E-07	0.232115262	4
Ghr	2.10E-07	0.189590948	4
Gde1	2.19E-07	0.250069425	4
Creg1	2.78E-07	0.18508061	4
Rgs5	3.63E-07	0.224755816	4
Gstt1	3.81E-07	0.198093657	4

Olfm1	4.02E-07	0.176877955	4
Amy1	4.19E-07	0.212672157	4
Hsd3b7	7.04E-07	0.209924958	4
Ugt1a5	8.68E-07	0.215639538	4
Sord	9.07E-07	0.167929756	4
Chp1	9.65E-07	0.230275794	4
Fermt2	1.39E-06	0.210109776	4
Ptdss1	1.59E-06	0.22209204	4
Amacr	1.74E-06	0.19971615	4
Hspb6	1.95E-06	0.203215832	4
Pdilt	2.24E-06	0.198338678	4
Prodh	3.14E-06	0.220267143	4
Hist1h2bc	3.64E-06	0.174476201	4
Inmt	3.97E-06	0.165337702	4
Akr1a1	3.98E-06	0.177643851	4
Ephx1	4.00E-06	0.17940713	4
Slco1a1	4.03E-06	0.177383627	4
Slc16a12	4.44E-06	0.203827802	4
Egfl7	5.39E-06	0.21679218	4
Apoa2	5.51E-06	0.139480921	4
Anxa2	6.39E-06	0.220506543	4
Car3	6.69E-06	0.137597813	4
Pxdn	7.63E-06	0.207394417	4
Nat8f2	7.68E-06	0.169173658	4
Prlr	9.44E-06	0.222311654	4
Atp11c	9.46E-06	0.224231595	4
Rbp4	1.24E-05	0.148293739	4
Mbl1	1.39E-05	0.192531064	4
Ecm1	1.40E-05	0.214876688	4
Akr1c14	1.40E-05	0.214339185	4
Idh2	2.28E-05	0.172640798	4
Adgrl4	2.34E-05	0.200059748	4
Stab1	2.84E-05	0.21734988	4
Abcc3	2.93E-05	0.204076198	4
Tmem134	2.96E-05	0.200938586	4
Tex264	3.11E-05	0.216961979	4
Pah	3.29E-05	0.152349697	4
Ces3a	3.64E-05	0.152390753	4
Pctp	5.06E-05	0.20911247	4
Pam	5.94E-05	0.23475515	4
Lgals1	6.74E-05	0.181796812	4
Inhbc	7.84E-05	0.206730505	4
Gba2	0.000159015	0.151605467	4
Olfr1033	0.00024965	0.152527157	4
Ptprb	0.00036691	0.190065804	4
Lum	0.000445381	0.17672367	4
Cpox	0.00045071	0.17983375	4
Lifr	0.000461118	0.176993749	4
Acot4	0.000464537	0.165013118	4
Cyp2c70	0.000496868	0.137249232	4
Lamp2	0.000517157	0.151240535	4
Ehhadh	0.000747639	0.16505532	4
Lamb1	0.000773753	0.155855086	4
Gstm6	0.000789864	0.160882438	4
Abcd3	0.000860349	0.144642834	4
Paqr9	0.001183495	0.143413995	4
D630039A03Rik	0.001269286	0.178722246	4
Eci1	0.00127447	0.16321567	4
Gm4756	0.001494423	0.182752225	4

Slc25a20	0.001676997	0.168757647	4
Alad	0.001822901	0.180071678	4
Cyp2d22	0.001860291	0.169739306	4
Fcgrt	0.001918023	0.168012193	4
BC004004	0.001939704	0.187190698	4
Ccdc162	0.001950249	0.150360502	4
Gatm	0.002106625	0.181060926	4
Cavin2	0.002231373	0.187312384	4
Acsm1	0.002259551	0.154582925	4
Ntn1	0.002501241	0.165844108	4
Tmem56	0.002599612	0.184684571	4
Plxnc1	0.00289236	0.165373722	4
Mettl7b	0.004066011	0.143454226	4
Ccdc107	0.004634033	0.183992491	4
Eif4g2	0.005843158	0.141216893	4
Pfkfb1	0.006303184	0.168158664	4
Cd36	0.007008622	0.17089041	4
Sparc	0.007589156	0.193646828	4
Dnase1l3	0.008159113	0.169252557	4
Cdh2	0.008602419	0.18344098	4
Stab2	0.01268487	0.182301883	4
Nceh1	0.014600161	0.165329529	4
Slc29a1	0.015405217	0.151660369	4
Gas6	0.02044734	0.048752644	4
Slc22a23	0.021245613	0.159703773	4
Igsf11	0.022673062	0.182866924	4
Naca	0.025479338	0.151032806	4
Pigp	0.025569004	0.159844527	4
Hmgcl	0.026290396	0.132384291	4
Pde2a	0.027186223	0.168732534	4
Add3	0.029809631	0.158980472	4
Hspa1b	0.035536103	0.123507649	4
Cd164	0.037405526	0.154845726	4
Hint2	0.038793547	0.152772134	4
Abhd6	0.040284514	0.160202465	4
Habp2	0.041822865	0.154000315	4
Ppm1k	0.048917636	0.146644272	4
Igfbp7	0.049162918	0.137281045	4
Aars	0.049746555	0.14525716	4
Oat	5.82E-124	1.552215587	5
Cyp2e1	4.37E-166	1.533385545	5
Rgn	8.19E-201	1.287053637	5
Cyp2c29	1.42E-182	1.384778081	5
Acaa1b	9.76E-180	1.120846821	5
Aldh3a2	2.51E-176	1.403600925	5
Gstm1	3.65E-168	1.069402183	5
Gsta3	1.65E-150	0.823030415	5
Rnase4	9.36E-143	0.943107249	5
Cyp1a2	3.02E-142	1.333522947	5
Aldh1a1	1.10E-139	1.217477965	5
Gulo	8.25E-135	1.166194329	5
Akr1c6	2.35E-128	1.108467766	5
Slc22a1	8.05E-128	1.147425755	5
Car3	1.46E-127	0.729820403	5
Sord	5.72E-123	0.884975412	5
Inmt	1.27E-121	0.79174032	5
Aldh2	1.23E-120	0.752735768	5
Lect2	4.68E-119	1.112050639	5

Csad	1.11E-118	1.035614467	5
Slco1b2	2.45E-115	0.977112265	5
Pon1	9.27E-115	0.755740228	5
Paqr9	2.94E-108	0.850946349	5
Acox1	3.28E-106	0.640442093	5
Acot1	1.72E-105	0.908778444	5
Ang	3.67E-100	0.834696231	5
Eci2	1.46E-97	0.775185041	5
Aqp9	2.84E-96	0.81745355	5
Cyp7a1	2.85E-96	0.925479401	5
Cyb5a	1.20E-95	0.64106049	5
Rdh16	1.84E-94	0.845135663	5
Blvrb	2.13E-93	0.897481973	5
Cyp27a1	8.40E-93	0.726306433	5
Mgst1	8.03E-92	0.599718972	5
Cldn2	3.83E-91	0.832264824	5
Hpd	1.69E-87	0.585601397	5
Gstm3	2.95E-86	0.975436698	5
Nr1i3	4.30E-86	0.823108794	5
Sult2a8	7.58E-80	0.690663616	5
Nrn1	1.02E-79	0.676231417	5
Fitm1	1.26E-79	0.794999546	5
Gstm2	7.67E-78	0.834386725	5
Akr1c20	9.90E-78	0.790922583	5
Phyh	1.64E-77	0.740860369	5
Cyp2c54	4.30E-76	0.998439376	5
Prodh	1.11E-75	0.860072474	5
Prdx6	3.59E-75	0.63362641	5
Ugt2b1	7.97E-75	0.646359237	5
Cyp4a14	5.31E-74	0.84082798	5
Coq8a	1.60E-73	0.634385081	5
Cyp2a4	6.33E-73	0.774811977	5
C6	5.10E-72	0.698710172	5
Hmgcl	9.81E-72	0.594765185	5
Ugt1a1	1.49E-71	0.702259731	5
Slc19a2	9.62E-71	0.633316708	5
Hmgcs2	6.93E-70	0.56318437	5
Serpina7	4.54E-69	0.91861756	5
Por	1.28E-68	0.653765257	5
Entpd5	3.21E-67	0.591196289	5
Cpox	5.80E-67	0.806702566	5
Scp2	2.84E-65	0.511202133	5
Hsd17b4	8.29E-65	0.562741515	5
Acsm1	2.95E-64	0.589529073	5
Adh1	5.84E-64	0.463976749	5
Fabp1	9.45E-64	0.552779539	5
Slc1a2	5.94E-63	1.359174123	5
Npr2	1.77E-62	0.858740291	5
Ephx2	2.23E-62	0.52757919	5
Gstm6	3.57E-61	0.633698025	5
Susd4	4.10E-61	0.643800843	5
Glud1	4.13E-61	0.504271873	5
Cdo1	5.47E-61	0.520011683	5
Tsc22d1	5.80E-61	0.752408319	5
Decr2	8.68E-61	0.576811385	5
Ehhadh	9.93E-61	0.584099262	5
Lhpp	1.04E-59	0.660973013	5
Cyp4a10	1.80E-59	0.686790775	5
Bdh1	2.11E-59	0.525823259	5

Slc16a10	4.78E-59	0.790230371	5
Tst	9.67E-59	0.615209122	5
Rbp4	2.70E-58	0.433508192	5
Ces2c	3.96E-58	0.861231229	5
Slco1a1	6.16E-58	0.544915541	5
Sod1	3.28E-57	0.409072888	5
Tmem205	8.41E-57	0.49091661	5
Abcc3	1.22E-56	0.693675069	5
Eci1	3.04E-56	0.56333156	5
Nat8f2	5.03E-56	0.559212082	5
Ces1g	5.05E-55	0.593446445	5
Csrp3	5.69E-55	0.625437374	5
Cyp2c69	1.39E-54	0.681914318	5
Gcdh	1.42E-54	0.58736837	5
Hmgcs1	2.60E-54	0.569168623	5
Cyb5b	3.54E-54	0.567770296	5
Hsd3b7	4.02E-54	0.543449952	5
Cyp2c68	5.62E-54	0.643760437	5
Gstz1	1.63E-53	0.566091489	5
Alad	7.74E-53	0.597405826	5
Lonp2	8.22E-53	0.496218123	5
Aldh4a1	1.15E-52	0.603221541	5
Gstt1	6.52E-52	0.516070277	5
Pctp	2.16E-51	0.587659097	5
Abcd3	8.41E-51	0.44736538	5
Fmo1	9.35E-51	0.572085087	5
Cs	1.37E-50	0.547903194	5
Comt	2.30E-50	0.534019921	5
Dhrs1	2.83E-50	0.609667849	5
Slc13a3	8.10E-50	0.505125127	5
Axin2	1.00E-49	0.457688262	5
Aox1	2.74E-49	0.558978435	5
Mup17	6.81E-49	1.11920319	5
Hsd17b10	1.21E-48	0.581210579	5
Iqgap2	1.48E-48	0.590478067	5
Creg1	2.20E-48	0.427409369	5
Egln3	5.23E-48	0.530260892	5
Cyp3a11	8.47E-48	0.470318937	5
Me1	1.41E-46	0.583117791	5
Ugt2b34	3.08E-46	0.568477496	5
Slc27a2	6.77E-46	0.444981711	5
Sardh	3.20E-45	0.476657798	5
Dmgdh	3.41E-45	0.532852859	5
Tbx3	4.89E-45	0.494483604	5
Fabp2	7.11E-45	0.520335991	5
Ugt2b35	7.80E-45	0.577305275	5
Notum	1.06E-44	0.580491051	5
Pah	1.48E-44	0.422578601	5
Hadh	1.72E-44	0.582502917	5
Rmdn2	2.09E-44	0.58229737	5
Itm2b	2.23E-44	0.470064907	5
Mbl1	5.89E-44	0.474346568	5
Acaa2	1.20E-43	0.37826313	5
Acadvl	2.91E-43	0.509427405	5
Gpd1	3.29E-43	0.525927056	5
Nxpe2	1.28E-42	0.458897082	5
Haao	3.37E-42	0.43902425	5
F5	1.24E-41	0.47136697	5
Dgat2	1.27E-41	0.456707822	5

Ccs	2.29E-41	0.514467803	5
Dcaf11	2.91E-41	0.462081327	5
Akr1a1	3.97E-41	0.414858694	5
Sgk2	6.39E-41	0.518876643	5
Ppara	7.59E-41	0.492630667	5
Idh1	8.31E-41	0.447101654	5
Mup11	1.08E-40	0.603592032	5
Papss2	1.09E-40	0.515255096	5
Kif1b	1.43E-40	0.55544144	5
Pank1	1.54E-40	0.513111493	5
Ldhd	2.25E-40	0.523734049	5
Dlat	4.19E-40	0.536403627	5
C2cd2	4.31E-40	0.52052475	5
Sqstm1	4.52E-40	0.452442524	5
Pparg	4.92E-40	0.334034666	5
Akr7a5	7.75E-40	0.473567269	5
Grhpr	8.35E-40	0.47623437	5
Hgd	9.80E-40	0.423255359	5
Sntb1	1.14E-39	0.550112338	5
Bphl	1.60E-39	0.471151135	5
Chp1	4.09E-39	0.535382013	5
Sephs2	4.33E-39	0.470577684	5
Tmem243	6.84E-39	0.486042665	5
Ces1d	3.03E-38	0.466200801	5
Hsd3b3	9.03E-38	0.478598252	5
Gpcpd1	1.11E-37	0.686813809	5
Fkbp8	1.24E-37	0.466755323	5
Tsku	1.36E-37	0.619710161	5
1810058I24Rik	1.46E-37	0.546214156	5
Crot	5.54E-37	0.384450638	5
Amacr	5.74E-37	0.447014246	5
4931406C07Rik	6.26E-37	0.485365461	5
Rarres1	7.73E-37	0.589147854	5
Got2	1.24E-36	0.445430154	5
Eif4g1	1.41E-36	0.447809812	5
Fmo5	1.43E-36	0.446176174	5
Ces3a	1.72E-36	0.353735583	5
Psmb7	3.08E-36	0.471615406	5
Rnf125	4.55E-36	0.388255596	5
Ndrg1	5.56E-36	0.375840089	5
Mocs1	6.71E-36	0.493252077	5
Bckdha	1.07E-35	0.419711285	5
Gjb1	1.38E-35	0.37642622	5
Cmbl	1.57E-35	0.469754122	5
Acox2	2.09E-35	0.397149828	5
Hadha	2.37E-35	0.535718599	5
Tspan12	3.30E-35	0.475393829	5
Serpina6	4.91E-35	0.482190869	5
Elovl3	5.97E-35	0.51998438	5
Mlxipl	7.09E-35	0.472663654	5
Nadk	7.73E-35	0.429995004	5
Acads	1.98E-34	0.444071731	5
Igfbp1	8.07E-34	0.503753447	5
Acat1	9.32E-34	0.470449934	5
Ech1	1.06E-33	0.429853417	5
Arrdc3	1.27E-33	0.506537872	5
Tenm3	1.98E-33	0.30285694	5
Cluh	2.53E-33	0.40555924	5
Akr1d1	2.56E-33	0.485915332	5

Nfic	2.81E-33	0.477650534	5
Acaa1a	3.21E-33	0.522647776	5
Aars	3.26E-33	0.483576608	5
Slc25a10	4.09E-33	0.42586487	5
Hdgf	5.49E-33	0.420586493	5
Mpc2	6.97E-33	0.412539571	5
Pex11a	7.02E-33	0.502681604	5
Pnp	1.01E-32	0.479154928	5
Acadl	1.25E-32	0.410258689	5
Acsm5	1.41E-32	0.53797262	5
Rxra	1.62E-32	0.454230767	5
Bcl9l	2.02E-32	0.488712623	5
Hspd1	3.52E-32	0.396741248	5
Cyb5r3	4.33E-32	0.35769583	5
Grpel1	4.77E-32	0.393097324	5
Atp5b	5.17E-32	0.381063617	5
1600014C10Rik	5.61E-32	0.44198516	5
Aspscr1	6.32E-32	0.489841999	5
Clpx	7.99E-32	0.436737875	5
Chpt1	1.18E-31	0.486485143	5
Apoe	1.40E-31	0.331183556	5
Decr1	2.52E-31	0.357004749	5
Agpat2	2.70E-31	0.439572971	5
Acsm3	3.70E-31	0.454300983	5
Abcg8	5.98E-31	0.46315748	5
Txn1	6.10E-31	0.370996892	5
Abcg2	6.21E-31	0.496760632	5
Vwa8	1.25E-30	0.455682792	5
Nit2	1.30E-30	0.461982448	5
Wdtc1	1.30E-30	0.494716594	5
Etnk2	1.34E-30	0.421547878	5
Ptms	2.25E-30	0.348953431	5
Ndufa6	3.26E-30	0.401453899	5
Acacb	3.61E-30	0.535870758	5
Tmbim6	4.11E-30	0.356494001	5
Pcsk6	4.28E-30	0.461907488	5
Akr1c14	4.48E-30	0.47032564	5
Avpr1a	5.74E-30	0.315486378	5
Idh2	6.19E-30	0.489473381	5
Tex264	6.65E-30	0.441786316	5
Car5a	7.50E-30	0.487625598	5
Ube2e2	9.75E-30	0.406789623	5
Mettl26	9.79E-30	0.434212431	5
Cyp2c70	1.03E-29	0.361180771	5
Hpn	1.04E-29	0.383354834	5
Plpp3	1.10E-29	0.431675828	5
Stard10	1.26E-29	0.330983333	5
Ttpa	2.07E-29	0.363440364	5
Dhcr24	2.85E-29	0.439278894	5
Tmprss6	3.02E-29	0.420904148	5
Aldh9a1	3.18E-29	0.407375953	5
Ak3	5.62E-29	0.473692389	5
Abcc2	8.63E-29	0.421962215	5
Qdpr	1.25E-28	0.372688491	5
Apoc4	1.41E-28	0.326602469	5
Ugt1a5	1.70E-28	0.493082356	5
Acadm	2.29E-28	0.413172699	5
Fbxo21	3.68E-28	0.432687203	5
Ftl1-ps1	4.58E-28	0.265176227	5

Dnaja1	5.55E-28	0.44250324	5
Wrnip1	6.03E-28	0.450487727	5
Agxt2	6.58E-28	0.436109625	5
Pgrmc1	6.71E-28	0.353555799	5
Hsd3b5	7.09E-28	0.462362023	5
Isoc2a	7.52E-28	0.442110455	5
Adk	9.72E-28	0.342503105	5
Shmt2	9.87E-28	0.430422736	5
Acss2	1.15E-27	0.437088221	5
Arhgef19	1.18E-27	0.427288915	5
Pcbd1	1.20E-27	0.35424956	5
Vnn1	1.21E-27	0.412960798	5
D630039A03Rik	1.29E-27	0.40791123	5
Ubxn1	1.98E-27	0.407854961	5
Upb1	2.01E-27	0.38275764	5
Slc16a12	2.44E-27	0.439954612	5
Glrx5	2.64E-27	0.389618632	5
Chchd10	3.30E-27	0.470717317	5
Coasy	5.01E-27	0.441508803	5
Cyc1	5.44E-27	0.369915797	5
Cnbp	6.88E-27	0.314559824	5
Anp32a	1.30E-26	0.355612962	5
Mettl7b	1.36E-26	0.335760827	5
Cyp4f14	1.82E-26	0.3617712	5
Tm7sf2	2.15E-26	0.403362405	5
Echs1	2.40E-26	0.365612929	5
Fbxo3	2.47E-26	0.380175254	5
Pdha1	2.64E-26	0.432644286	5
Mcc	4.73E-26	0.459319929	5
Plcxd2	7.91E-26	0.508629523	5
Msrb1	8.33E-26	0.347509743	5
Hsdl2	8.71E-26	0.402440973	5
Dpys	9.61E-26	0.400542408	5
Rexo2	9.99E-26	0.410488834	5
Ahr	1.09E-25	0.432307121	5
Aco1	1.34E-25	0.394785733	5
Pafah2	1.59E-25	0.41465683	5
Suox	1.63E-25	0.392842083	5
Nadk2	1.72E-25	0.415629745	5
Cyp2d10	1.89E-25	0.415321404	5
Apoa2	2.85E-25	0.256867589	5
Mia3	2.94E-25	0.414679739	5
Hint1	3.60E-25	0.443255132	5
C4bp	3.94E-25	0.283843825	5
Acad11	5.17E-25	0.410182637	5
Ces2e	5.46E-25	0.412376384	5
Anxa7	5.59E-25	0.420086159	5
Pcx	6.14E-25	0.320672478	5
Aldh6a1	6.16E-25	0.309479632	5
Hebp1	6.23E-25	0.380868254	5
Tnfaip8l1	7.50E-25	0.393983123	5
Prdx5	9.23E-25	0.381929505	5
Pink1	9.45E-25	0.437948186	5
Stk16	1.08E-24	0.432844222	5
Ddt	1.25E-24	0.39319251	5
Dhdh	1.77E-24	0.410271523	5
Eef2	1.81E-24	0.350879284	5
Ahcyl1	2.13E-24	0.394166314	5
Etfdh	2.42E-24	0.321699314	5

Xdh	2.43E-24	0.48207769	5
Sema4g	2.77E-24	0.372502043	5
Psmd4	3.21E-24	0.371637973	5
Spp2	3.84E-24	0.351333944	5
Gm4756	3.92E-24	0.407229121	5
Acbd5	4.30E-24	0.409538081	5
Col18a1	4.31E-24	0.392625034	5
Mmd	4.57E-24	0.437444014	5
Grina	6.13E-24	0.406040043	5
Fam107b	6.50E-24	0.40979843	5
Cyp4a12b	8.88E-24	0.388982723	5
Wbp1l	8.95E-24	0.37306881	5
Hagh	9.95E-24	0.35747179	5
Tomm70a	1.14E-23	0.366394812	5
Sec14l2	1.20E-23	0.317462913	5
Atp5a1	1.49E-23	0.301735154	5
Aldh7a1	1.51E-23	0.376280236	5
Gde1	1.67E-23	0.432696276	5
Fpgs	1.76E-23	0.405860982	5
Psmb4	1.87E-23	0.344321236	5
Amy1	2.01E-23	0.317259267	5
Lpin2	2.11E-23	0.400628115	5
Gsr	2.19E-23	0.367600933	5
Crat	2.43E-23	0.459250119	5
Tfr2	2.72E-23	0.354191233	5
Gamt	2.75E-23	0.395831865	5
Slc22a23	3.01E-23	0.42477944	5
Gclm	4.81E-23	0.394635304	5
Dbi	6.20E-23	0.259161946	5
Pcyt2	6.77E-23	0.332241173	5
Glo1	8.83E-23	0.384002712	5
Xylb	9.27E-23	0.367493162	5
Mup10	9.64E-23	0.583638102	5
Nploc4	1.03E-22	0.383873692	5
Pfkfb1	1.28E-22	0.428084531	5
Nipsnap1	1.75E-22	0.357327512	5
Pygl	1.89E-22	0.357769212	5
Atp11c	2.38E-22	0.411161081	5
Hint2	2.59E-22	0.34898571	5
Nr1h4	3.00E-22	0.3892961	5
Rdh7	3.03E-22	0.28464857	5
Slc25a42	3.14E-22	0.406181502	5
Ube4b	3.21E-22	0.401663826	5
Agmat	3.51E-22	0.376030007	5
Gclc	3.77E-22	0.319828565	5
Eps8l2	3.87E-22	0.376143746	5
Eif5	4.64E-22	0.313618311	5
Zfyve1	4.79E-22	0.372655851	5
Fermt2	6.21E-22	0.354563198	5
Chdh	6.37E-22	0.388933218	5
Fasn	6.73E-22	0.435546722	5
Ndfip1	9.57E-22	0.343942381	5
Mocs2	1.38E-21	0.388656933	5
Fdx1	1.49E-21	0.397820173	5
Ilrun	1.58E-21	0.329314264	5
Sec61b	2.71E-21	0.368394339	5
Amfr	2.77E-21	0.365376063	5
Prlr	3.24E-21	0.383893571	5
Rnf187	3.27E-21	0.383854729	5

Ly6g2	3.83E-21	0.335220164	5
Gne	4.01E-21	0.393202918	5
Slc16a7	4.06E-21	0.347901631	5
Pxdc1	4.44E-21	0.40212845	5
Hao1	4.82E-21	0.328820733	5
Ctdsp1	4.94E-21	0.341142896	5
Stard7	5.54E-21	0.375837232	5
Mat1a	5.56E-21	0.285582204	5
Cat	5.64E-21	0.260414088	5
Hspb8	5.86E-21	0.392162839	5
Fam25c	6.16E-21	0.367174297	5
Spr	6.34E-21	0.362005732	5
Lpgat1	6.48E-21	0.373247065	5
Tle5	6.63E-21	0.292333524	5
Etfa	6.67E-21	0.34726744	5
Cebpb	7.68E-21	0.452525377	5
Erbb3	9.08E-21	0.384577017	5
Pex19	9.21E-21	0.408891773	5
Lrp1	1.06E-20	0.425774093	5
Reep6	1.07E-20	0.319249533	5
Gstm4	1.39E-20	0.387249101	5
Ubqln1	1.39E-20	0.336998075	5
Slc25a39	1.48E-20	0.289733938	5
Dhrs3	1.89E-20	0.330246095	5
Fkbp4	2.42E-20	0.385570534	5
Sh3d19	2.64E-20	0.380802313	5
Skp1a	2.71E-20	0.323596363	5
Rack1	3.75E-20	0.274196073	5
Gbe1	3.91E-20	0.347524376	5
Mgll	4.22E-20	0.373334031	5
Vcp	4.28E-20	0.333261063	5
Hectd1	4.45E-20	0.4088366	5
Pdzk1	4.66E-20	0.390158235	5
Alkbh5	4.76E-20	0.37688557	5
Ltbr	5.47E-20	0.36363435	5
Adtrp	5.58E-20	0.371123974	5
Sirt3	6.10E-20	0.409932311	5
Dcxr	7.94E-20	0.367374499	5
Aup1	9.33E-20	0.358801755	5
Tbcel	9.91E-20	0.375722614	5
Pex14	1.23E-19	0.374995259	5
Rapgef4	1.72E-19	0.363920579	5
Mfn2	1.75E-19	0.402612386	5
Iah1	1.88E-19	0.34478586	5
Gpld1	1.91E-19	0.342731119	5
Atox1	1.95E-19	0.348193193	5
Trap1	2.21E-19	0.323103259	5
Acnat1	2.48E-19	0.351287624	5
Ugdh	3.08E-19	0.38030339	5
Ptpmt1	3.08E-19	0.336316439	5
Plbd1	3.20E-19	0.322358249	5
Gchfr	3.26E-19	0.326736381	5
Pim1	3.26E-19	0.325847235	5
Psmb5	3.28E-19	0.368854	5
Hspa1b	3.47E-19	0.306944573	5
Nudc	3.52E-19	0.347480389	5
Sec14l4	3.60E-19	0.3480472	5
Strap	4.32E-19	0.400426683	5
Eif4a2	4.42E-19	0.298138604	5

Prkd3	4.69E-19	0.380204174	5
Rnf43	5.36E-19	0.241575914	5
Pccb	5.42E-19	0.38653719	5
Tspo	5.46E-19	0.326764738	5
Arf6	5.52E-19	0.366218229	5
Ttc38	5.78E-19	0.383140758	5
Adi1	5.92E-19	0.290204065	5
Pgm1	5.97E-19	0.358190692	5
Cpt2	6.08E-19	0.392836679	5
Pex6	6.40E-19	0.38105534	5
Hpgd	6.50E-19	0.42102637	5
Gpd2	6.82E-19	0.341327856	5
Abhd6	7.42E-19	0.35630238	5
Hsd17b11	7.88E-19	0.375466982	5
Slc49a4	8.04E-19	0.369743308	5
Bckdhb	8.96E-19	0.339646743	5
Trp53inp2	1.23E-18	0.375984979	5
Macrod1	1.25E-18	0.337657675	5
Txnrd1	1.40E-18	0.384388996	5
Dhrs4	2.13E-18	0.313887823	5
Hacd3	2.46E-18	0.346177322	5
Pid1	2.63E-18	0.35324889	5
Etfbkmt	2.64E-18	0.397540528	5
Rab43	2.65E-18	0.37025579	5
Abcb4	3.18E-18	0.354824628	5
Gcgr	3.40E-18	0.319897402	5
Tex2	3.45E-18	0.359955188	5
Psmb3	4.40E-18	0.365156066	5
Psmd1	4.53E-18	0.352523624	5
Nqo2	5.55E-18	0.321919674	5
C77080	5.87E-18	0.395044253	5
Pdxk	5.97E-18	0.350020101	5
Tlcd2	6.59E-18	0.394512421	5
Slc27a5	6.61E-18	0.287418688	5
Klf15	7.02E-18	0.389439758	5
Sult1d1	7.18E-18	0.42022356	5
Asgr1	7.45E-18	0.298421877	5
Phb2	7.47E-18	0.367672675	5
Ghr	8.43E-18	0.283971231	5
Prpf8	8.76E-18	0.361908665	5
Naprt	9.34E-18	0.378176641	5
Insig2	9.72E-18	0.319226244	5
Hba-a2	9.99E-18	0.554301838	5
Selenof	1.26E-17	0.333755283	5
Psmb6	1.29E-17	0.341378365	5
Pla2g6	1.42E-17	0.354912346	5
Serpina3m	1.51E-17	0.356368703	5
Rbbp4	1.57E-17	0.35799225	5
Ugp2	1.85E-17	0.367680254	5
Etfb	1.94E-17	0.336486335	5
Lactb2	1.95E-17	0.367444216	5
Pdcd6ip	2.05E-17	0.339949445	5
Slc25a20	2.13E-17	0.356771466	5
Slc25a13	2.17E-17	0.353579218	5
Psmc2	2.22E-17	0.33933929	5
Ppm1k	2.26E-17	0.309570859	5
Park7	2.29E-17	0.320017323	5
Zfp36l2	2.64E-17	0.471155985	5
Sec24a	2.65E-17	0.337235178	5

Hnf4a	2.70E-17	0.32588999	5
Slc25a33	2.95E-17	0.348182125	5
Acsl1	3.01E-17	0.324307917	5
Ghitm	3.12E-17	0.289889807	5
Slc35d1	3.16E-17	0.318449858	5
Ndufa10	3.38E-17	0.333616764	5
Retreg2	3.56E-17	0.340624846	5
Rnpepl1	3.60E-17	0.375653038	5
Fam210b	3.68E-17	0.372138064	5
Pmpcb	3.75E-17	0.304737464	5
Pklr	3.82E-17	0.333204133	5
Ugt2b36	3.85E-17	0.370646303	5
Tcf25	4.44E-17	0.362207996	5
Saraf	4.82E-17	0.331160781	5
Epb41l4b	5.40E-17	0.351386575	5
Tmem56	5.58E-17	0.339591787	5
Rgp1	5.72E-17	0.360317964	5
Txnl1	5.73E-17	0.331634986	5
Cryz	5.76E-17	0.342534713	5
Fech	6.31E-17	0.303043178	5
Cox6a1	6.56E-17	0.304929948	5
Zfp652	6.72E-17	0.343492525	5
Dgcr6	6.79E-17	0.367022764	5
Map1lc3a	7.27E-17	0.372568995	5
Cald1	7.31E-17	0.329673321	5
Atp5g3	7.44E-17	0.264559483	5
Cct8	9.97E-17	0.355092219	5
Gfra1	1.02E-16	0.346481816	5
Gucd1	1.06E-16	0.349807284	5
Rmnd5a	1.09E-16	0.345615444	5
Dpp9	1.21E-16	0.34200986	5
Atp13a3	1.36E-16	0.326575685	5
Eef1a1	1.43E-16	0.21400286	5
Slc47a1	1.44E-16	0.365431301	5
Rassf8	1.45E-16	0.35087603	5
Cpt1a	1.59E-16	0.34502494	5
Slc25a1	1.75E-16	0.360142418	5
Hspb6	1.79E-16	0.290859974	5
Thnsl2	2.13E-16	0.36355945	5
Ppp1ca	2.24E-16	0.358233978	5
Gatd3a	2.44E-16	0.328919144	5
Atp5d	2.51E-16	0.255657595	5
Mia2	2.53E-16	0.303795851	5
Selenoo	2.72E-16	0.324794324	5
Slc25a21	2.79E-16	0.216001727	5
Arl6ip1	3.11E-16	0.328798951	5
Stard5	3.12E-16	0.365863722	5
Hnrnpab	3.46E-16	0.310057452	5
Lypla1	4.04E-16	0.318785227	5
Mttp	4.20E-16	0.327138866	5
Acot4	4.28E-16	0.314409496	5
Egln2	4.76E-16	0.340048518	5
Hacl1	4.95E-16	0.312134176	5
Rnf10	5.06E-16	0.324568767	5
Aamp	6.68E-16	0.305706608	5
Ndufs2	6.79E-16	0.290695973	5
Agpat3	7.11E-16	0.297626193	5
Akip1	7.37E-16	0.34735103	5
Bbox1	7.45E-16	0.36317147	5

Isoc1	8.08E-16	0.329001898	5
Gpat4	9.66E-16	0.312149609	5
Htatip2	9.84E-16	0.325032384	5
Prkaa2	1.05E-15	0.361003976	5
Psma7	1.09E-15	0.355155771	5
Cd302	1.11E-15	0.256681468	5
Pipox	1.12E-15	0.307715803	5
Sepsecs	1.29E-15	0.351068365	5
Gpt2	1.46E-15	0.352863997	5
Map3k5	1.46E-15	0.349728317	5
Ubr3	1.55E-15	0.33592677	5
Abcc6	1.56E-15	0.33472479	5
Aldh1a7	1.56E-15	0.309413621	5
Pebp1	1.65E-15	0.335062448	5
Dusp6	1.72E-15	0.312997522	5
Optn	1.87E-15	0.338065514	5
Gatad2a	2.03E-15	0.38044584	5
Plxnb2	2.05E-15	0.343147217	5
Urod	2.10E-15	0.357571349	5
Pnpla7	2.12E-15	0.299004973	5
Psma1	2.31E-15	0.349418605	5
Psen2	2.33E-15	0.310161703	5
Suds3	2.45E-15	0.330967131	5
Adh5	2.47E-15	0.265636012	5
Fcgrt	2.60E-15	0.294908774	5
Gnas	2.62E-15	0.315137472	5
Hrg	2.62E-15	0.256877108	5
Pex5	2.71E-15	0.325369596	5
Rnaseh2c	2.85E-15	0.330520619	5
Hspa9	3.07E-15	0.269228355	5
Cmas	3.34E-15	0.347751828	5
Cct3	3.39E-15	0.324793262	5
Pdk4	3.53E-15	0.248759195	5
Atf5	3.60E-15	0.318407977	5
Pex7	4.05E-15	0.340562099	5
Pdilt	4.12E-15	0.291970988	5
Gm4951	4.15E-15	0.321603822	5
Galk1	4.23E-15	0.341989883	5
Slc4a4	4.38E-15	0.353450704	5
Lgals9	4.68E-15	0.274158779	5
Ptprd	4.90E-15	0.360306706	5
Cib1	4.90E-15	0.300739047	5
Metap1d	5.04E-15	0.316837332	5
Shmt1	5.07E-15	0.328148547	5
Kank1	5.49E-15	0.325244387	5
Gpam	5.89E-15	0.345560892	5
Onecut2	6.01E-15	0.365913589	5
Gabarapl1	6.21E-15	0.299007245	5
G0s2	7.97E-15	0.355810035	5
Hdlbp	8.04E-15	0.279015038	5
Fah	8.31E-15	0.244841058	5
Msra	8.73E-15	0.27903062	5
Gch1	9.24E-15	0.293508798	5
Hbb-bs	1.13E-14	0.623481399	5
Cyp2d22	1.32E-14	0.288731295	5
Mif	1.42E-14	0.305875294	5
Fgf1	1.42E-14	0.33742936	5
Afmid	1.45E-14	0.350881561	5
Gba2	1.56E-14	0.24877405	5

Cyp2j5	1.68E-14	0.26884231	5
Smdt1	1.71E-14	0.328171367	5
Oaz1	1.72E-14	0.297443461	5
Nlrp6	2.04E-14	0.310841566	5
Ddx3x	2.08E-14	0.292835776	5
Hprt	2.15E-14	0.332850623	5
Dennd5b	2.17E-14	0.335858169	5
Micos13	2.35E-14	0.301010954	5
Atl3	2.45E-14	0.306511734	5
Hectd3	2.58E-14	0.361346089	5
Aadat	2.64E-14	0.312459672	5
Psmc4	2.86E-14	0.324574134	5
Pde3b	3.66E-14	0.288867064	5
Cdh2	3.71E-14	0.316571248	5
Nectin1	4.11E-14	0.309355779	5
Npc1	4.16E-14	0.3166963	5
Faah	4.24E-14	0.286424467	5
Sdr42e1	4.38E-14	0.348466961	5
Cpeb4	4.39E-14	0.315092874	5
Prpf19	4.52E-14	0.342446339	5
Colec12	4.54E-14	0.28242047	5
Mettl7a1	4.82E-14	0.291842415	5
Palmd	4.96E-14	0.325212246	5
Ide	5.05E-14	0.296220793	5
Ndufa8	5.78E-14	0.322870346	5
Gstm7	5.98E-14	0.334933026	5
Cdc34	6.07E-14	0.344388488	5
Caprin1	7.06E-14	0.261980729	5
Chuk	7.74E-14	0.309186738	5
Olfr1033	7.93E-14	0.258192902	5
Car8	9.52E-14	0.313789724	5
Kif1c	1.00E-13	0.330384999	5
Echdc2	1.01E-13	0.281522298	5
Tango2	1.33E-13	0.332737582	5
Bhmt2	1.37E-13	0.301297891	5
Cisd1	1.44E-13	0.294668322	5
Fads1	1.77E-13	0.31707721	5
Urah	1.88E-13	0.236270746	5
Ablim3	1.93E-13	0.296912567	5
Pemt	1.94E-13	0.243368433	5
Otulin	2.10E-13	0.322756856	5
Tle1	2.25E-13	0.304325118	5
Ubl3	2.33E-13	0.296146218	5
Lifr	2.35E-13	0.302642727	5
Aacs	2.52E-13	0.368574982	5
Psmd2	2.56E-13	0.335653181	5
Nt5e	2.79E-13	0.226900898	5
Pex16	2.99E-13	0.335362698	5
Adra1b	3.30E-13	0.322721472	5
Uqcrc1	3.53E-13	0.30245899	5
Clmn	3.61E-13	0.321730644	5
Glyctk	3.75E-13	0.330551063	5
Hadhb	3.83E-13	0.302343538	5
Psme4	4.33E-13	0.355411737	5
Nucks1	4.54E-13	0.327729975	5
C130074G19Rik	4.56E-13	0.291740687	5
Spc24	5.41E-13	0.267735613	5
Oplah	5.55E-13	0.361360449	5
Pnpo	6.87E-13	0.304973469	5

Edem1	7.19E-13	0.25859806	5
Mapkapk2	7.23E-13	0.309176028	5
Yy1	7.31E-13	0.312031001	5
Bpnt1	7.54E-13	0.306119886	5
Slc29a1	7.82E-13	0.272799782	5
Sh3bgrl2	8.07E-13	0.283845664	5
Wdr6	8.21E-13	0.217800062	5
Gck	8.61E-13	0.287667976	5
Dio1	8.66E-13	0.32934468	5
Gfm1	9.10E-13	0.293553405	5
Dynlrb1	9.35E-13	0.318603902	5
Psma5	9.49E-13	0.318740822	5
Eif4h	9.59E-13	0.297228778	5
Serp1	1.04E-12	0.269447117	5
Aifm1	1.09E-12	0.323408837	5
Spg20	1.20E-12	0.309113743	5
Shld2	1.20E-12	0.36196843	5
Hoga1	1.21E-12	0.28481647	5
Fbxo22	1.34E-12	0.320086248	5
Dus1l	1.43E-12	0.285874219	5
Tbc1d9b	1.54E-12	0.330230136	5
Ccdc107	1.55E-12	0.316956188	5
Dock4	1.56E-12	0.29521197	5
Sbk1	1.62E-12	0.194286665	5
Rcbtb1	1.75E-12	0.291502609	5
Dynll2	2.19E-12	0.254318585	5
Cltb	2.31E-12	0.303093162	5
Acot8	2.37E-12	0.305455266	5
Rab7	2.43E-12	0.295257646	5
Ephx1	2.44E-12	0.239563969	5
Csk	2.46E-12	0.306950378	5
Selenot	2.59E-12	0.270445167	5
Lims2	2.71E-12	0.314248225	5
Cyp4a32	2.78E-12	0.297009247	5
Cobll1	2.84E-12	0.299380499	5
Nedd4	3.61E-12	0.257867382	5
Sar1b	3.73E-12	0.259632529	5
Lrp5	3.76E-12	0.28577027	5
Igfbp4	3.91E-12	0.238368088	5
Ptp4a2	4.07E-12	0.304903087	5
Prdx3	4.10E-12	0.320024698	5
Flot1	4.21E-12	0.286576083	5
Ptprf	4.29E-12	0.289358567	5
Fam210a	4.37E-12	0.318502581	5
Znrf3	4.55E-12	0.244540409	5
Pik3r1	4.56E-12	0.342111349	5
Apoh	4.65E-12	0.200304418	5
Gda	4.76E-12	0.306590355	5
Mbl2	4.80E-12	0.302484507	5
Pla2g12a	4.93E-12	0.286883189	5
Nbr1	5.22E-12	0.319027235	5
Sptbn2	5.60E-12	0.304028333	5
Pir	6.21E-12	0.283788413	5
A1cf	6.26E-12	0.247660882	5
Rab11a	6.59E-12	0.271251016	5
Tmem30a	6.87E-12	0.31764866	5
Lpl	7.11E-12	0.386308688	5
Tmem134	7.15E-12	0.306960624	5
BC004004	7.39E-12	0.263451488	5

Nceh1	7.48E-12	0.275174028	5
Irs1	7.53E-12	0.258302389	5
Usp47	8.23E-12	0.343319477	5
Nav2	8.36E-12	0.306653573	5
Dars	8.43E-12	0.312783428	5
Psmd7	8.92E-12	0.296799429	5
Grb7	9.38E-12	0.309802668	5
Gm16286	9.38E-12	0.309271843	5
Fgfrl1	9.61E-12	0.332524575	5
Tapt1	1.02E-11	0.317405392	5
Pmvk	1.03E-11	0.350476937	5
Rab5if	1.04E-11	0.289456603	5
Ahctf1	1.14E-11	0.27170558	5
Cdk6	1.23E-11	0.289399236	5
Glyat	1.32E-11	0.279106443	5
Map2k2	1.43E-11	0.294676955	5
Ppp2r5a	1.52E-11	0.280847802	5
Gm4952	1.69E-11	0.240209741	5
Phldb2	1.73E-11	0.32201923	5
Abhd14b	1.77E-11	0.30245767	5
Pabpc1	1.82E-11	0.23761967	5
Aldh5a1	1.85E-11	0.298414052	5
Ppp1r3b	1.87E-11	0.283785322	5
Fitm2	1.90E-11	0.300596633	5
Cebpa	1.96E-11	0.256625422	5
Selenoi	2.14E-11	0.290051696	5
Ugt1a6b	2.19E-11	0.302082815	5
Apoc1	2.26E-11	0.1803553	5
Cyp2c55	2.28E-11	0.265485586	5
Akr1c12	2.31E-11	0.292174225	5
Ldah	2.33E-11	0.280704192	5
Ubap1	2.33E-11	0.280501338	5
Nudt12	2.35E-11	0.258967227	5
Camk2n1	2.39E-11	0.290687951	5
Dnajc22	2.59E-11	0.294311801	5
Lman2	2.65E-11	0.252100939	5
Mfsd4b1	2.67E-11	0.317458536	5
Trp53inp1	2.71E-11	0.323443677	5
Dmxl2	2.81E-11	0.282331355	5
Abhd15	3.04E-11	0.288956207	5
Cd81	3.04E-11	0.216012478	5
Ttc36	3.10E-11	0.202044967	5
Ppp6r1	3.14E-11	0.320006546	5
Hspe1	3.16E-11	0.286752997	5
Suclg2	3.24E-11	0.289966304	5
Mindy1	3.28E-11	0.323883343	5
Capn1	3.31E-11	0.278407994	5
Acot12	3.45E-11	0.295184233	5
Usp24	3.68E-11	0.309243639	5
Pitpnm2	3.83E-11	0.299291007	5
Rbm47	3.83E-11	0.315902272	5
Nr2f6	3.91E-11	0.297516708	5
Aldh8a1	3.92E-11	0.242377352	5
Fggy	4.03E-11	0.305694259	5
Pnpla8	4.44E-11	0.313950039	5
Kdm5c	4.45E-11	0.305284736	5
Srrm2	4.56E-11	0.274567565	5
Bri3	5.04E-11	0.292418569	5
Mpdu1	5.09E-11	0.278991212	5

Pxmp4	6.06E-11	0.286740293	5
Birc6	6.32E-11	0.277153915	5
Slc25a30	7.51E-11	0.2596823	5
Khk	7.65E-11	0.220133783	5
Huwe1	7.66E-11	0.300664029	5
Cep85	8.43E-11	0.300141317	5
Adh4	8.79E-11	0.315236106	5
Slc16a2	9.31E-11	0.278564502	5
Psma6	1.02E-10	0.262814854	5
Timm13	1.14E-10	0.272515197	5
Trim14	1.17E-10	0.28331961	5
Gphn	1.21E-10	0.304252922	5
Ndst1	1.29E-10	0.300415696	5
Spint2	1.38E-10	0.309056737	5
Tmem150a	1.38E-10	0.2984685	5
Fbxo31	1.45E-10	0.287135963	5
Gtf2i	1.57E-10	0.282257018	5
Slc6a9	1.69E-10	0.280922527	5
Sidt2	1.71E-10	0.296032839	5
Adhfe1	1.77E-10	0.287484684	5
Prxl2c	1.82E-10	0.299690661	5
Alas1	1.84E-10	0.555767486	5
Furin	1.85E-10	0.263823038	5
Brap	1.86E-10	0.30501776	5
Farp2	1.86E-10	0.22715733	5
Impad1	1.86E-10	0.287694087	5
Abcf1	1.92E-10	0.26768727	5
Dnaja2	2.09E-10	0.280642163	5
Ubr4	2.21E-10	0.305825025	5
Gstt3	2.26E-10	0.284112262	5
Yaf2	2.30E-10	0.200001549	5
Rab5a	2.36E-10	0.254899987	5
Snx3	2.37E-10	0.25744033	5
Eif4b	2.44E-10	0.245414091	5
Rab17	2.46E-10	0.224376032	5
Dazap2	2.55E-10	0.287464168	5
Chchd3	2.71E-10	0.260455991	5
Psmd8	2.78E-10	0.262910576	5
Pqlc1	2.91E-10	0.293926546	5
Man2a2	2.99E-10	0.253594316	5
Slc35e2	3.03E-10	0.281492853	5
Rex1bd	3.31E-10	0.288974967	5
Nfe2l1	3.39E-10	0.285905458	5
Psmd5	3.47E-10	0.270062039	5
Ndrg2	3.48E-10	0.224076635	5
Ndufs1	3.48E-10	0.278930875	5
Fetub	3.49E-10	0.204771534	5
Arf1	3.56E-10	0.275062377	5
Mtfr1	3.75E-10	0.27671518	5
Atp5c1	3.84E-10	0.282108193	5
Pdk2	4.10E-10	0.275687779	5
Fbxo9	4.10E-10	0.288275061	5
Slc17a8	4.20E-10	0.221146819	5
Psmc3	4.40E-10	0.275315419	5
Mknk2	4.52E-10	0.275457274	5
Nsdhl	4.53E-10	0.301434157	5
Cd1d1	5.04E-10	0.305630692	5
Sec23a	5.28E-10	0.257987012	5
Lipa	5.33E-10	0.254848283	5

Mmut	5.38E-10	0.245976823	5
Coq9	5.40E-10	0.276212619	5
Pnkd	5.41E-10	0.267125474	5
Sem1	5.64E-10	0.247700511	5
Hmbs	5.87E-10	0.263528028	5
Pard3	5.96E-10	0.269307231	5
Ndufs5	5.97E-10	0.264741433	5
Bcar3	6.20E-10	0.270036649	5
Retreg3	6.23E-10	0.27430978	5
Arel1	6.56E-10	0.280821995	5
Rdx	6.75E-10	0.278383244	5
Eef1b2	6.87E-10	0.203620769	5
Impa1	6.90E-10	0.267185266	5
Tgfa	7.27E-10	0.252850903	5
Gspt1	7.39E-10	0.28784362	5
Rspo3	7.74E-10	0.27113358	5
Cct5	7.87E-10	0.270851239	5
Ccn1	7.88E-10	0.211198641	5
Mfhas1	8.05E-10	0.258952909	5
Hnrnpul2	8.05E-10	0.290987688	5
Akap1	8.24E-10	0.293767667	5
Vamp8	8.26E-10	0.206780443	5
Agfg2	8.32E-10	0.245016161	5
Pa2g4	8.91E-10	0.270129088	5
Immt	9.05E-10	0.272152657	5
Psmd13	1.00E-09	0.273073991	5
Scap	1.04E-09	0.262148264	5
Slc35a3	1.05E-09	0.278478066	5
Bccip	1.07E-09	0.25022233	5
Preb	1.09E-09	0.280430661	5
Farsb	1.11E-09	0.285633215	5
Bsg	1.11E-09	0.189878253	5
Fbxl5	1.12E-09	0.29623497	5
Pim3	1.12E-09	0.33252032	5
Erap1	1.13E-09	0.285484641	5
Mbd3	1.15E-09	0.253373725	5
Prkcz	1.20E-09	0.176416087	5
Hs6st1	1.27E-09	0.32231189	5
Malsu1	1.29E-09	0.274028522	5
Abcb6	1.30E-09	0.261535823	5
Csnk2a1	1.32E-09	0.281098349	5
Sult1b1	1.36E-09	0.226796536	5
Net1	1.41E-09	0.303386751	5
Irf2bp2	1.46E-09	0.290587272	5
Ndufa12	1.46E-09	0.302487684	5
Oxsm	1.51E-09	0.27291263	5
Ap1g1	1.52E-09	0.283231423	5
Sec24c	1.55E-09	0.261109081	5
Arl5a	1.55E-09	0.282435145	5
Poldip2	1.57E-09	0.276152667	5
Mbd2	1.60E-09	0.251888741	5
Lonp1	1.61E-09	0.285636368	5
Mthfd1	1.89E-09	0.252624441	5
Arpp19	1.91E-09	0.305434569	5
Rnh1	1.95E-09	0.289724631	5
Cpn1	1.96E-09	0.21559791	5
Habp2	2.02E-09	0.233452675	5
Smagp	2.08E-09	0.288724784	5
Clpb	2.14E-09	0.280174842	5

Pnrc1	2.15E-09	0.316146042	5
Kpnb1	2.26E-09	0.267346187	5
Irs2	2.31E-09	0.250377557	5
Tsta3	2.36E-09	0.269672402	5
Copa	2.41E-09	0.258133613	5
Psmd3	2.49E-09	0.250946913	5
Dhx9	2.63E-09	0.273648971	5
Cops5	2.65E-09	0.273364807	5
Psmd12	2.74E-09	0.248499196	5
Maged1	2.88E-09	0.299299727	5
Josd1	2.91E-09	0.243720116	5
Timmdc1	2.92E-09	0.288432425	5
Sra1	2.95E-09	0.231876541	5
Map2k1	2.98E-09	0.298724521	5
Ndufv3	2.99E-09	0.278290946	5
N4bp2	3.03E-09	0.265172193	5
Leprotl1	3.05E-09	0.275131214	5
Lgalsl	3.17E-09	0.289649666	5
Wwc1	3.28E-09	0.271465534	5
5-Mar	3.34E-09	0.256727566	5
Rad23b	3.36E-09	0.278169291	5
Sema4a	3.42E-09	0.296679231	5
Coa6	3.47E-09	0.241164978	5
Mbtps1	3.71E-09	0.271615098	5
Cct6a	3.92E-09	0.263969563	5
Wasl	3.97E-09	0.268402895	5
Coq10a	4.06E-09	0.300175333	5
Ccdc85b	4.10E-09	0.243960339	5
Tmem238	4.27E-09	0.234485493	5
Agl	4.48E-09	0.262455531	5
Fbxo8	4.54E-09	0.282811656	5
Psma3	4.78E-09	0.249530591	5
N4bp2l2	4.86E-09	0.265559978	5
Ahcy	4.87E-09	0.281431619	5
Pmpca	5.31E-09	0.261621623	5
Foxa1	5.63E-09	0.22366627	5
Tmem19	5.90E-09	0.293950625	5
Scamp3	6.26E-09	0.241413823	5
Nfyb	6.43E-09	0.264695004	5
Washc2	6.45E-09	0.264100631	5
Dbt	6.63E-09	0.286941075	5
Tcp1	6.72E-09	0.253669576	5
Pex13	6.81E-09	0.277562512	5
Usp10	6.83E-09	0.275816962	5
Riok3	6.95E-09	0.246328402	5
Dnajb2	7.05E-09	0.256210878	5
Fads2	7.05E-09	0.248942221	5
Bnip3	7.18E-09	0.250973616	5
Pgls	7.19E-09	0.279071994	5
Sgms2	7.46E-09	0.268966342	5
Hhex	7.55E-09	0.307026045	5
Ankrd40	7.57E-09	0.286041475	5
1300017J02Rik	7.77E-09	0.199392369	5
Prkar2a	7.77E-09	0.277940664	5
Iars2	8.44E-09	0.285497624	5
Lmf1	8.59E-09	0.26756094	5
Sec31a	8.77E-09	0.25944857	5
Mical3	8.79E-09	0.223539936	5
Hectd4	8.81E-09	0.253917096	5

Cnpy2	9.17E-09	0.269325306	5
Acsl5	9.59E-09	0.249087388	5
Psmc6	9.67E-09	0.290491843	5
Mrap	9.77E-09	0.291699067	5
1810055G02Rik	9.85E-09	0.239808425	5
Zdhhc9	9.87E-09	0.309574619	5
Fkbp2	1.02E-08	0.237945463	5
Nr1i2	1.06E-08	0.251239673	5
Slc31a1	1.07E-08	0.264703457	5
Rhbdd3	1.08E-08	0.227536197	5
Cox8a	1.12E-08	0.245425738	5
Chd3	1.12E-08	0.286308472	5
Pdap1	1.13E-08	0.266626126	5
Coa5	1.14E-08	0.258141733	5
Pbld1	1.19E-08	0.232663613	5
Taok3	1.32E-08	0.27191956	5
Ugt3a2	1.37E-08	0.229890364	5
Cdc37	1.41E-08	0.245774414	5
Gstk1	1.41E-08	0.254281115	5
Nsmf	1.49E-08	0.239034003	5
Eif2ak1	1.57E-08	0.274199252	5
Dhrs7	1.58E-08	0.249125067	5
Dera	1.86E-08	0.276460539	5
Ubxn4	1.92E-08	0.254056801	5
Atg5	1.96E-08	0.26700193	5
Snx9	2.01E-08	0.294544129	5
Rad54l2	2.08E-08	0.246183536	5
Hist1h2bc	2.13E-08	0.206814937	5
Inhbe	2.29E-08	0.27310433	5
Mrfap1	2.31E-08	0.252799273	5
Pum1	2.33E-08	0.297549069	5
Glt1d1	2.38E-08	0.166329804	5
Agmo	2.40E-08	0.258468778	5
Ecsit	2.56E-08	0.276612546	5
Snd1	2.56E-08	0.254168143	5
Glod4	2.58E-08	0.27085735	5
Sod2	2.74E-08	0.227944816	5
Srsf6	2.86E-08	0.274733083	5
Abcg5	2.95E-08	0.295790415	5
Hspa4	3.06E-08	0.263683455	5
Mcfd2	3.35E-08	0.236618323	5
Gcat	3.53E-08	0.270091086	5
Dnpep	3.66E-08	0.248397081	5
Cct7	3.74E-08	0.266121794	5
Wdr26	3.86E-08	0.2487234	5
Eef1g	4.05E-08	0.229777531	5
Gorasp1	4.06E-08	0.238648892	5
Hsd17b7	4.17E-08	0.261070279	5
Ptpa	4.31E-08	0.238917293	5
Usf2	4.41E-08	0.262718017	5
Slc25a37	4.41E-08	0.267420989	5
H6pd	4.95E-08	0.25676312	5
Dpy19l1	4.98E-08	0.279403943	5
Esrp2	4.98E-08	0.232789488	5
Mtfr1l	5.03E-08	0.259944932	5
Zfand5	5.08E-08	0.286814352	5
Hdac11	5.20E-08	0.230397378	5
Ptk2b	5.33E-08	0.27654868	5
Uqcrfs1	5.47E-08	0.186371721	5

Spop	5.49E-08	0.242480643	5
Nars2	5.51E-08	0.225531375	5
Larp1b	5.71E-08	0.249392388	5
Dpp3	5.89E-08	0.285284093	5
Larp1	6.02E-08	0.260437865	5
Mapk14	6.19E-08	0.2619445	5
Psmb2	6.22E-08	0.252856902	5
Lap3	6.24E-08	0.210000293	5
Rab5b	6.45E-08	0.231245041	5
Atoh8	6.48E-08	0.287032548	5
Trip4	7.19E-08	0.280244649	5
Cideb	7.87E-08	0.19672575	5
Lgals8	8.01E-08	0.247834313	5
Sf3b2	8.17E-08	0.24194453	5
Gna12	8.40E-08	0.293936179	5
Dsc2	9.11E-08	0.237985313	5
Gtf2h5	9.13E-08	0.259121631	5
Bola1	9.27E-08	0.240825162	5
Uqcrc2	9.44E-08	0.217304147	5
Hgs	9.49E-08	0.246933827	5
Naa38	9.53E-08	0.243206889	5
Ube2r2	1.03E-07	0.235343315	5
Wipi2	1.03E-07	0.254213189	5
Ywhae	1.03E-07	0.260179762	5
Cox19	1.04E-07	0.256421943	5
Fahd1	1.05E-07	0.229681678	5
Atp5f1	1.05E-07	0.266129984	5
Rnf217	1.08E-07	0.238411933	5
Usp9x	1.10E-07	0.281201992	5
2810459M11Rik	1.15E-07	0.243368675	5
Ccdc162	1.21E-07	0.202760129	5
Ssrp1	1.22E-07	0.24745727	5
Phf10	1.25E-07	0.226127721	5
Arfgef1	1.28E-07	0.254459067	5
Txn2	1.36E-07	0.206439118	5
Pnpla2	1.53E-07	0.268720286	5
Cds2	1.55E-07	0.25938898	5
Myo1b	1.57E-07	0.233212714	5
Thrsp	1.60E-07	0.269449571	5
Vps13d	1.61E-07	0.269123472	5
Psmd6	1.64E-07	0.246579172	5
Bscl2	1.71E-07	0.245844582	5
Reep3	1.72E-07	0.264428728	5
Shroom2	1.73E-07	0.223684962	5
Wbp2	1.76E-07	0.229669295	5
Tmem14c	1.83E-07	0.219093236	5
Zfp740	1.85E-07	0.231648006	5
Lias	1.95E-07	0.258647544	5
Clpp	1.98E-07	0.259301425	5
Kdm5b	2.02E-07	0.199629674	5
Met	2.08E-07	0.249965561	5
Gbf1	2.09E-07	0.278957615	5
Mlycd	2.11E-07	0.227015651	5
Wdr18	2.11E-07	0.282226321	5
Trip12	2.45E-07	0.242767068	5
Scd1	2.47E-07	0.402448537	5
Tmem59	2.48E-07	0.215276576	5
Mpst	2.48E-07	0.26767935	5
Aasdhppt	2.55E-07	0.230300555	5

Atp6v1h	2.56E-07	0.268466494	5
Tpmt	2.62E-07	0.250799247	5
Ccny	2.71E-07	0.240459394	5
Ubp1	2.94E-07	0.239255951	5
Rhou	3.00E-07	0.248170377	5
Bco2	3.00E-07	0.208056527	5
Efr3a	3.03E-07	0.238876681	5
Rer1	3.10E-07	0.198745212	5
Zbtb20	3.17E-07	0.234123734	5
Phb	3.18E-07	0.235642227	5
Vps41	3.25E-07	0.242697764	5
Arhgef3	3.32E-07	0.227773385	5
Elmo3	3.56E-07	0.172500428	5
Lurap1l	3.58E-07	0.267970714	5
Gak	3.59E-07	0.252379615	5
Rffl	3.63E-07	0.2300867	5
Ptpn11	4.06E-07	0.222784352	5
Numa1	4.07E-07	0.217611675	5
Trim28	4.08E-07	0.244713994	5
Slc45a3	4.18E-07	0.248846659	5
Vmp1	4.25E-07	0.244780925	5
Hist1h1c	4.32E-07	0.21674867	5
Efhd2	4.35E-07	0.251016763	5
Tmem37	4.53E-07	0.26556047	5
Slc36a1	4.64E-07	0.234410033	5
Psma4	4.73E-07	0.247165204	5
Glrx	4.81E-07	0.204665811	5
Asb13	4.91E-07	0.244917729	5
Adipor2	4.92E-07	0.2026241	5
Lamp2	4.97E-07	0.175795178	5
Foxn3	5.19E-07	0.230322276	5
Abcb10	5.22E-07	0.257353573	5
Pfdn2	5.38E-07	0.243829652	5
Trir	5.45E-07	0.250877021	5
Gpt	5.46E-07	0.258561707	5
Suclg1	5.56E-07	0.234761053	5
Arhgef12	5.61E-07	0.245240979	5
Pcbp1	5.70E-07	0.236758528	5
Lpcat3	5.79E-07	0.247101171	5
L2hgdh	5.79E-07	0.212084603	5
Stat5b	5.86E-07	0.268244873	5
Klhl24	6.04E-07	0.248214574	5
Ngly1	6.10E-07	0.229374638	5
Vps35	6.26E-07	0.245740345	5
4932438A13Rik	6.31E-07	0.229498379	5
Mtmr4	6.34E-07	0.22599126	5
Akt1s1	6.39E-07	0.222995385	5
Mfsd1	6.74E-07	0.238797314	5
Spcs1	6.81E-07	0.239262634	5
Creld1	7.08E-07	0.238425491	5
Snap47	7.11E-07	0.256196835	5
Nsun2	7.33E-07	0.265934236	5
Dstn	7.37E-07	0.233744177	5
Ak2	7.65E-07	0.234808569	5
Ndufs7	7.76E-07	0.225688704	5
Mdh2	7.78E-07	0.259581219	5
Gfer	8.22E-07	0.234402592	5
Ppip5k2	8.39E-07	0.236379682	5
Gabarapl2	8.65E-07	0.226974477	5

Serbp1	8.70E-07	0.244734828	5
Med25	8.96E-07	0.248993856	5
Phlda1	9.09E-07	0.408913857	5
Rnf103	9.15E-07	0.232284693	5
Rad23a	9.18E-07	0.26087226	5
Epn1	9.58E-07	0.244896061	5
Eif3k	9.63E-07	0.215511614	5
Atg7	9.99E-07	0.216861018	5
Osbpl9	1.03E-06	0.280627436	5
Eif4ebp2	1.03E-06	0.212508053	5
Acsl4	1.05E-06	0.249196697	5
Ugt2a3	1.12E-06	0.230318432	5
Retsat	1.20E-06	0.221711298	5
Erg28	1.21E-06	0.237868935	5
Tmem184a	1.21E-06	0.197865555	5
Vps26a	1.21E-06	0.224637707	5
Abhd17c	1.26E-06	0.226944588	5
Mlec	1.27E-06	0.23557046	5
Ibtk	1.31E-06	0.25644072	5
Opa1	1.34E-06	0.24970293	5
Ndufb6	1.38E-06	0.213941607	5
Gatc	1.38E-06	0.191756022	5
Lamtor4	1.39E-06	0.219077957	5
1-Mar	1.40E-06	0.193014042	5
Ube2l6	1.43E-06	0.266296151	5
D230025D16Rik	1.44E-06	0.279284431	5
Pdk1	1.48E-06	0.255646699	5
Rnf123	1.49E-06	0.263360503	5
Eif4g2	1.52E-06	0.187697648	5
Phyhd1	1.53E-06	0.226139775	5
Plg	1.54E-06	0.162990303	5
Cflar	1.54E-06	0.22729153	5
Slco2b1	1.55E-06	0.27448452	5
Snrnp70	1.55E-06	0.240270019	5
Depdc7	1.57E-06	0.234761925	5
Ces2a	1.59E-06	0.193592205	5
Vkorc1	1.66E-06	0.216591882	5
Acadsb	1.69E-06	0.247514098	5
Ranbp9	1.70E-06	0.255944256	5
Zfp445	1.71E-06	0.241657149	5
Cenpb	1.71E-06	0.249765666	5
Ei24	1.85E-06	0.207189391	5
Epg5	1.87E-06	0.183716883	5
Ndufv1	2.07E-06	0.209054768	5
Smcr8	2.07E-06	0.206998721	5
Brd4	2.15E-06	0.267866074	5
Spag9	2.16E-06	0.236105017	5
Hnrnpu	2.16E-06	0.221642426	5
Osbpl1a	2.17E-06	0.214968983	5
Eaf1	2.27E-06	0.21764544	5
Tob1	2.29E-06	0.2273365	5
Onecut1	2.30E-06	0.413095366	5
Mcat	2.31E-06	0.204222124	5
Pon3	2.32E-06	0.244408141	5
S100a10	2.32E-06	0.245519339	5
Pja1	2.34E-06	0.217168227	5
Rab1b	2.36E-06	0.234046805	5
Crls1	2.38E-06	0.223010954	5
Galnt4	2.41E-06	0.183563213	5

Fam91a1	2.42E-06	0.234643519	5
Wipi1	2.45E-06	0.222155434	5
Gstt2	2.48E-06	0.256857604	5
Brd2	2.49E-06	0.24034625	5
Chchd1	2.53E-06	0.226723278	5
Arid1b	2.74E-06	0.219636059	5
Lrpprc	2.79E-06	0.250715877	5
Ugt2b5	2.89E-06	0.199892485	5
Tysnd1	2.94E-06	0.214957134	5
Slc38a10	2.99E-06	0.232467208	5
Sgta	3.09E-06	0.240519258	5
Zfp598	3.29E-06	0.249912309	5
Hibch	3.31E-06	0.237469352	5
Cebpz	3.31E-06	0.179625743	5
Ywhag	3.44E-06	0.238775794	5
Gys2	3.46E-06	0.215949011	5
Proc	3.56E-06	0.179779436	5
Chkb	3.58E-06	0.254131828	5
Uqcc3	3.61E-06	0.221393137	5
Scand1	3.61E-06	0.209481487	5
L3hypdh	3.67E-06	0.215701065	5
Nfe2l2	3.74E-06	0.237627011	5
Mgam	3.78E-06	0.247596632	5
Ube2m	3.82E-06	0.223543144	5
Sdha	3.88E-06	0.202160488	5
Dctn3	3.89E-06	0.245674275	5
Bhmt	3.92E-06	0.272941648	5
Cenpx	4.01E-06	0.243699916	5
Bet1	4.08E-06	0.21743512	5
Nr1h3	4.09E-06	0.222330743	5
Nol9	4.10E-06	0.163555053	5
Slc48a1	4.32E-06	0.216093576	5
Smim4	4.34E-06	0.232423869	5
Jtb	4.39E-06	0.259606839	5
Iscu	4.55E-06	0.22928728	5
Hipk2	4.65E-06	0.264183594	5
Sar1a	4.73E-06	0.223534165	5
Klf12	4.79E-06	0.183065402	5
Mrnip	4.79E-06	0.180400472	5
Taf15	4.85E-06	0.251058368	5
Edc3	4.86E-06	0.219322005	5
Hsd17b2	5.04E-06	0.210410411	5
Traf4	5.13E-06	0.207210733	5
Cyp2c23	5.14E-06	0.177728408	5
Psmf1	5.16E-06	0.239015152	5
Eif1	5.40E-06	0.240463895	5
Nedd8	5.52E-06	0.205059379	5
Dcun1d1	5.61E-06	0.242808286	5
Xpnpep1	5.65E-06	0.213459297	5
Upf1	5.75E-06	0.222175787	5
Fzd7	5.81E-06	0.199057447	5
Stau1	5.85E-06	0.209066096	5
Ormdl3	5.85E-06	0.192436246	5
Tmed7	5.88E-06	0.218715256	5
Dpyd	5.89E-06	0.204625305	5
Daam1	6.11E-06	0.215084016	5
Id3	6.14E-06	0.290621802	5
Gpatch2	6.29E-06	0.217355668	5
AU022252	6.62E-06	0.230420925	5

Cops6	6.91E-06	0.202714574	5
Yap1	6.91E-06	0.230291154	5
Rab4a	6.97E-06	0.23803456	5
Slc9a3r1	7.05E-06	0.227406026	5
Srebf2	7.14E-06	0.232094153	5
Atg2b	7.24E-06	0.20824807	5
Drap1	7.32E-06	0.208030315	5
Scly	7.36E-06	0.228314127	5
Hsd17b12	7.85E-06	0.190173955	5
Sigmar1	8.06E-06	0.242415849	5
5031439G07Rik	8.23E-06	0.222437705	5
Mospd3	8.27E-06	0.238600931	5
Cmah	8.46E-06	0.227283326	5
Mettl27	8.55E-06	0.200601443	5
Irgq	8.61E-06	0.180118447	5
Abce1	8.67E-06	0.214737846	5
Csde1	8.67E-06	0.209345859	5
Slc2a2	8.69E-06	0.230906337	5
Naxd	8.70E-06	0.241833373	5
Tprkb	9.03E-06	0.240821838	5
Tor1a	9.05E-06	0.204945333	5
Slc25a5	9.10E-06	0.201199461	5
Polr2m	9.25E-06	0.231076385	5
Garem1	9.45E-06	0.205392582	5
Cacul1	9.52E-06	0.224901532	5
Csnk1d	9.55E-06	0.22095126	5
Sec16a	9.68E-06	0.229960849	5
Bahcc1	9.74E-06	0.150385708	5
Hyal2	1.00E-05	0.222969782	5
Babam1	1.05E-05	0.252732572	5
Enpp3	1.05E-05	0.209044422	5
Map3k13	1.06E-05	0.195529885	5
Hsp90aa1	1.06E-05	0.217910907	5
Klhl2	1.09E-05	0.266899538	5
Atg9a	1.11E-05	0.238226162	5
Iigp1	1.15E-05	0.327402581	5
Gadd45gip1	1.16E-05	0.231750526	5
C1ra	1.17E-05	0.239426115	5
Sorbs1	1.17E-05	0.242725865	5
Pitrm1	1.27E-05	0.226093253	5
Tmtc2	1.27E-05	0.147786955	5
Api5	1.28E-05	0.219238697	5
Larp4b	1.29E-05	0.244118642	5
Ndufs6	1.31E-05	0.254612935	5
Maco1	1.32E-05	0.213021976	5
Ccdc6	1.37E-05	0.212725566	5
0610040J01Rik	1.41E-05	0.16500096	5
Uqcr11	1.43E-05	0.191108502	5
1190005I06Rik	1.43E-05	0.145013047	5
Nelfe	1.45E-05	0.212370624	5
Cgnl1	1.46E-05	0.237801273	5
Opa3	1.47E-05	0.22956214	5
Pex1	1.48E-05	0.225078939	5
Actb	1.50E-05	0.182298335	5
Trim25	1.51E-05	0.234718468	5
Nubp2	1.51E-05	0.235358456	5
Nsd3	1.53E-05	0.209004024	5
Cdc42ep4	1.54E-05	0.228015998	5
Amotl2	1.55E-05	0.207461759	5

Znhit1	1.57E-05	0.234485493	5
Mtln	1.59E-05	0.22900256	5
Ppdpf	1.62E-05	0.250936902	5
Polr2f	1.64E-05	0.223298658	5
Trim11	1.65E-05	0.216909296	5
Dap3	1.67E-05	0.237900761	5
Prpf6	1.68E-05	0.220211362	5
Psmd14	1.71E-05	0.220604146	5
Ulk2	1.73E-05	0.25293973	5
Cog4	1.76E-05	0.217835868	5
Tut7	1.78E-05	0.200673019	5
Clcn2	1.86E-05	0.193893645	5
Pecr	1.90E-05	0.236060202	5
St3gal3	1.91E-05	0.195950424	5
Pdcd2	1.96E-05	0.187074841	5
Wdr61	1.98E-05	0.21179443	5
Abat	1.99E-05	0.21639001	5
Stk11	2.05E-05	0.219673223	5
Hnrnpl	2.05E-05	0.230918051	5
Tmem208	2.12E-05	0.22822259	5
Tmem64	2.15E-05	0.218201833	5
Tspan31	2.18E-05	0.184060876	5
Mcm10	2.19E-05	0.249473615	5
Bst2	2.20E-05	0.195818697	5
Trpc4ap	2.22E-05	0.235246856	5
Tcf20	2.22E-05	0.222238067	5
Sirt7	2.26E-05	0.227848041	5
Igsf11	2.28E-05	0.23127183	5
Nfix	2.30E-05	0.238155956	5
Bag4	2.32E-05	0.194647774	5
Tbl3	2.37E-05	0.182438746	5
Hp	2.37E-05	0.197770639	5
Pde4dip	2.38E-05	0.199897892	5
Als2	2.38E-05	0.192768357	5
Ccnd3	2.39E-05	0.191073232	5
Smim19	2.42E-05	0.189499941	5
Crebzf	2.45E-05	0.226311562	5
Rtcb	2.52E-05	0.197273426	5
Akr1c13	2.59E-05	0.237344786	5
Syne1	2.65E-05	0.19547724	5
Rufy3	2.66E-05	0.220173078	5
Ddi2	2.66E-05	0.262012692	5
Vti1b	2.67E-05	0.234403678	5
Crk	2.67E-05	0.207512009	5
Nars	2.69E-05	0.216949407	5
Atp9a	2.76E-05	0.221175964	5
Cbr4	2.80E-05	0.219738459	5
Sdhc	2.86E-05	0.180468896	5
Pgpep1	2.94E-05	0.209160415	5
Txndc5	2.96E-05	0.204640081	5
Rab1a	2.99E-05	0.190685856	5
Cox7a2	3.09E-05	0.177069046	5
Slc25a45	3.23E-05	0.215009788	5
Hint3	3.25E-05	0.236930262	5
Lrrc58	3.25E-05	0.193879383	5
Mmadhc	3.30E-05	0.223360355	5
Ap2a2	3.39E-05	0.214706756	5
Rheb	3.42E-05	0.204030973	5
Dyrk2	3.43E-05	0.252761517	5

Sphk2	3.49E-05	0.221993107	5
Erbin	3.51E-05	0.217862998	5
Polr2e	3.51E-05	0.22170938	5
Ptdss1	3.51E-05	0.255545609	5
Rab14	3.53E-05	0.207993236	5
Txnrd2	3.57E-05	0.222493516	5
Thap12	3.65E-05	0.20682915	5
Aass	3.73E-05	0.201646323	5
Grtp1	3.78E-05	0.220134008	5
Ap3m1	3.85E-05	0.231853722	5
Rnf114	3.91E-05	0.209699097	5
Krt8	4.03E-05	0.200371705	5
Surf4	4.11E-05	0.193865622	5
Ccng2	4.24E-05	0.227093835	5
Cgrrf1	4.24E-05	0.234903242	5
Slc17a4	4.25E-05	0.225494376	5
Prkacb	4.29E-05	0.214508782	5
Rnf11	4.34E-05	0.216353243	5
Mif4gd	4.47E-05	0.234798224	5
Elk4	4.57E-05	0.2142561	5
Gatb	4.58E-05	0.205709117	5
Tor1aip1	4.69E-05	0.216810601	5
Get4	4.86E-05	0.208682554	5
Golga4	4.89E-05	0.22535693	5
Eif3l	5.06E-05	0.240483572	5
Ube2b	5.12E-05	0.215461483	5
Ddah1	5.15E-05	0.252344015	5
Rrn3	5.19E-05	0.199970118	5
Grcc10	5.21E-05	0.23439448	5
Acat2	5.38E-05	0.255386252	5
Fam8a1	5.43E-05	0.220382323	5
Tm2d2	5.46E-05	0.192880693	5
Msl1	5.56E-05	0.227860017	5
Slc12a7	5.73E-05	0.214907912	5
Klhl5	5.78E-05	0.204193904	5
Churc1	5.79E-05	0.208336945	5
Eif3f	5.83E-05	0.185246743	5
Aurkaip1	6.21E-05	0.211000122	5
Cox15	6.25E-05	0.184985728	5
Lactb	6.35E-05	0.210873896	5
Ddrgk1	6.39E-05	0.219675212	5
Tubb4b	6.46E-05	0.258900141	5
Tmem135	6.49E-05	0.209431207	5
Npepl1	6.51E-05	0.209711651	5
Ptcd3	6.70E-05	0.192515047	5
S1pr1	6.75E-05	0.252491875	5
Plpbp	6.83E-05	0.226038884	5
Rorc	7.03E-05	0.184259755	5
Pef1	7.12E-05	0.191282922	5
Tk1	7.34E-05	0.187170303	5
Capns1	7.41E-05	0.192869677	5
Paqr7	7.41E-05	0.217711304	5
Ptp4a1	7.52E-05	0.201749737	5
Senp3	7.55E-05	0.14360169	5
Galm	7.72E-05	0.195321226	5
Phactr4	7.79E-05	0.186657894	5
Pgam1	7.91E-05	0.211047931	5
Man1a	8.04E-05	0.195205408	5
Krcc1	8.05E-05	0.21551658	5

Atp6v0d1	8.08E-05	0.204464507	5
Khdrbs3	8.24E-05	0.17088236	5
Dtx3l	8.34E-05	0.23523553	5
Commd6	8.41E-05	0.217320682	5
Apba3	8.46E-05	0.213746689	5
Pskh1	8.54E-05	0.192655378	5
Rtp3	8.59E-05	0.221717615	5
Mars	8.75E-05	0.204176087	5
Kit	8.88E-05	0.169980722	5
Mb21d2	9.09E-05	0.197726943	5
Dtnbp1	9.26E-05	0.2067837	5
Zfhx3	9.55E-05	0.216519369	5
Hdhd3	9.72E-05	0.214850171	5
Cyp8b1	9.74E-05	0.189679239	5
Fbxo6	9.79E-05	0.230490309	5
Rab3ip	9.80E-05	0.219102154	5
Rnf13	0.000100751	0.19748171	5
Yme1l1	0.000100871	0.189467347	5
Uba1	0.000101147	0.19854815	5
Polg	0.000102865	0.20124993	5
Ammecr1l	0.000103966	0.215842905	5
Sdf4	0.000106736	0.161981525	5
Mtus1	0.000108081	0.234645251	5
Ppp1r16a	0.000112834	0.1551451	5
Tmem223	0.000115346	0.203656102	5
Cul4a	0.000115943	0.246075905	5
Pdpr	0.000118637	0.181514179	5
Nit1	0.000119474	0.204770399	5
Hsph1	0.000119704	0.208535802	5
Dap	0.000120476	0.216638319	5
Adcy9	0.000123661	0.159687707	5
Slc38a2	0.000123958	0.244396377	5
Lrrc14	0.000124999	0.151366774	5
Unkl	0.000125149	0.154548997	5
Ube2o	0.000125867	0.138689932	5
Cd164	0.000127992	0.164929338	5
Mlf2	0.000134352	0.223424616	5
Rab8a	0.00013526	0.19671496	5
Map1lc3b	0.000135724	0.191602808	5
Srsf5	0.000136894	0.202020594	5
Igf2r	0.000137118	0.19844575	5
Srr	0.000137155	0.188739903	5
Dctn2	0.000138788	0.221839384	5
Cltc	0.000139117	0.185471401	5
2610507B11Rik	0.000139858	0.215856114	5
Sephs1	0.000141092	0.224847411	5
Mocos	0.000141317	0.262331046	5
Aplp2	0.000142906	0.164668957	5
Ndufb1-ps	0.000145814	0.166260964	5
Rtf2	0.000146107	0.237796427	5
Prcc	0.00014835	0.194946017	5
Tjp3	0.000152467	0.170809105	5
Cradd	0.000153141	0.200950395	5
Arhgap35	0.000153364	0.206229143	5
Dtymk	0.000154775	0.19632284	5
Atrn	0.000156252	0.20036344	5
Sox6	0.000161185	0.161742026	5
Tsc2	0.000161563	0.177929269	5
Scrn2	0.000161746	0.206315016	5

Ap2m1	0.00016218	0.184224189	5
Gcn1	0.000164917	0.204594616	5
Plk3	0.000167863	0.24980772	5
Pxmp2	0.000167865	0.208010217	5
Abca6	0.000168372	0.184748239	5
Borcs5	0.000168878	0.169335583	5
Rnf167	0.000169875	0.210408763	5
Actr1b	0.000171801	0.185247624	5
Nt5dc1	0.00017415	0.208577323	5
Pum2	0.000175422	0.185526588	5
Usp46	0.000178716	0.14271021	5
Ikbkb	0.000180491	0.218086618	5
Nmt2	0.000181873	0.185567055	5
R3hdm4	0.000183218	0.236910701	5
Napa	0.00018326	0.217234926	5
Eif4a1	0.00018738	0.248018649	5
Slc35e3	0.000188945	0.18346149	5
Ankrd13c	0.0001892	0.165167209	5
Cenpv	0.000190201	0.18227365	5
Mylk	0.000192722	0.202718448	5
Nfs1	0.000193523	0.223254919	5
Echdc3	0.000193857	0.209509497	5
Ggact	0.000194316	0.214057015	5
Ythdf1	0.000194853	0.213225493	5
Ndufa9	0.000194874	0.183085777	5
Chmp4b	0.000197566	0.230148241	5
H2-Ke6	0.000199852	0.188879365	5
Tm9sf3	0.000205194	0.202212831	5
Ube2g2	0.00020568	0.213473179	5
Mtpn	0.000208658	0.206693118	5
Flot2	0.000209592	0.2156946	5
Mrtfb	0.000210358	0.188358391	5
Pafah1b1	0.000211044	0.197719578	5
Psmc1	0.000213309	0.230734859	5
Ireb2	0.000213931	0.192677028	5
Map3k7	0.000215758	0.227217493	5
Nme1	0.000216651	0.223046656	5
Sgms1	0.000217076	0.176362845	5
C1galt1	0.000217838	0.210876519	5
BC005537	0.00022034	0.206791384	5
Tdrp	0.000227246	0.148934961	5
Retreg1	0.000229515	0.222325246	5
Spon2	0.000230664	0.196737136	5
Trim2	0.000231341	0.187077836	5
Ppp1r37	0.00023276	0.209155274	5
Oxr1	0.00023567	0.212476568	5
Pithd1	0.000237745	0.215469567	5
Pnkp	0.000239506	0.177094541	5
Ifnar2	0.000239602	0.206055525	5
Tmem214	0.000240843	0.198797832	5
Tufm	0.000242968	0.218616655	5
Vapb	0.000245027	0.211642791	5
Smpd2	0.000246448	0.185916593	5
Akt2	0.00024985	0.213328842	5
Smim10l1	0.000250239	0.222590067	5
Tax1bp1	0.000252695	0.189233852	5
Emc1	0.00025447	0.216810601	5
Tmed5	0.000256259	0.204251575	5
Ndufb9	0.000256953	0.207747225	5

Timm17b	0.000258991	0.160931902	5
Lrg1	0.000260582	0.170816714	5
Nrd1	0.00026383	0.196803499	5
Ttc39b	0.000264208	0.178663583	5
Use1	0.000264396	0.177695112	5
Swi5	0.000264617	0.205268135	5
Gnb2	0.000264959	0.173491426	5
Calcoco1	0.000265442	0.161422031	5
Pcdh1	0.000268458	0.207097626	5
Hbs1l	0.000272831	0.205095657	5
Lrit1	0.000273287	0.135287233	5
Gnai2	0.000277005	0.180953369	5
Hacd1	0.000278969	0.187586641	5
Rnf44	0.000279118	0.208619103	5
Ppp1r15b	0.000279985	0.225333795	5
Bcl6	0.000282492	0.211365612	5
Ppm1g	0.000289466	0.197956278	5
Lrrfip2	0.000295591	0.192053175	5
Gsk3a	0.000305727	0.229153035	5
Atp5j	0.000307656	0.171399573	5
Mcrip2	0.000310141	0.199940572	5
Ctbp1	0.000312099	0.204628807	5
Fabp5	0.000313494	0.301193239	5
Laptm4a	0.00031678	0.181936651	5
Romo1	0.000323985	0.208792515	5
Edem2	0.000324678	0.188495275	5
Chpf2	0.000330045	0.220821579	5
Trappc1	0.000338077	0.202064016	5
Ubr5	0.00034326	0.207202739	5
Tubb2a	0.000345423	0.21135094	5
Akr1c19	0.00035417	0.202653292	5
Rtn3	0.000357726	0.198676611	5
Hlf	0.000358721	0.201677027	5
Commd7	0.000364026	0.235298181	5
Uso1	0.000374313	0.205064233	5
Rad51d	0.000376823	0.148603688	5
Inhbc	0.000381051	0.204480591	5
Chn2	0.000382065	0.203626945	5
Zkscan3	0.00038318	0.206320778	5
Cttn	0.000395072	0.186431847	5
Rdh10	0.000395433	0.196913367	5
Arhgap5	0.000404339	0.199634213	5
Klc4	0.000407553	0.205791912	5
Gnl1	0.000411295	0.17533128	5
Srpr	0.000418158	0.190459093	5
Med8	0.000426127	0.201769317	5
Ap3d1	0.000426775	0.205462592	5
Selenbp1	0.000433404	0.209532814	5
Nags	0.000437429	0.213697471	5
Grb14	0.000443818	0.229271555	5
Hsf2	0.000446337	0.180092702	5
Cipc	0.000448512	0.193486046	5
Nfkbib	0.000448784	0.179112757	5
Slc10a1	0.000453089	0.178051893	5
Entpd8	0.000454871	0.227771538	5
Rmdn3	0.000458126	0.170055779	5
Ddx1	0.000460498	0.209108641	5
Lamtor2	0.000462553	0.186738284	5
Nfx1	0.000465049	0.198485294	5

Cfl2	0.000469501	0.205451193	5
Vapa	0.000487529	0.216057865	5
Cox5b	0.000498866	0.176811261	5
Micos10	0.000510306	0.206085468	5
Nr1h2	0.000510953	0.204231278	5
Prox1	0.000512439	0.188361246	5
Klhdc3	0.000514623	0.213792689	5
Zfpm1	0.000515328	0.209593883	5
Anp32b	0.000517271	0.173848232	5
Slc25a16	0.000525954	0.201277484	5
1110065P20Rik	0.000530987	0.173428925	5
Eif2a	0.000531476	0.194225505	5
Dcaf6	0.000532407	0.183496005	5
Pdcd6	0.000544256	0.215441122	5
Eif3e	0.000544702	0.184633622	5
Aagab	0.000551881	0.211386208	5
Srp72	0.000556684	0.213116008	5
C030006K11Rik	0.00056715	0.185708299	5
Saa4	0.000573053	0.266705322	5
Syncrip	0.000573684	0.199632948	5
Mospd2	0.000586204	0.195277307	5
Atraid	0.000590225	0.208356112	5
Fnip1	0.000593489	0.169351891	5
Eif2b5	0.000600089	0.196763232	5
Coq3	0.000609751	0.208474987	5
Tbc1d15	0.000618679	0.194410999	5
Arf4	0.000621423	0.169659775	5
Tkt	0.000622478	0.187845448	5
Ergic3	0.000625344	0.196718389	5
Gckr	0.000625871	0.182407779	5
Dlst	0.000626775	0.218695668	5
Supt5	0.000628021	0.207182349	5
Insr	0.000643911	0.230424819	5
Yars	0.000653172	0.187360957	5
Map7	0.00065595	0.167823001	5
Smarca2	0.00066313	0.190033776	5
Cdk5rap3	0.000664361	0.213690902	5
Sowahb	0.000675146	0.139620629	5
Scarb2	0.00067561	0.20237531	5
Kxd1	0.000680151	0.203328013	5
Tmem245	0.000699833	0.187814311	5
Dis3l	0.000704932	0.178410617	5
Vezt	0.000714862	0.17229296	5
Ociad1	0.000716957	0.196763232	5
Supt6	0.000719182	0.193988487	5
Psme1	0.000734592	0.208595014	5
Acot7	0.000741383	0.180770344	5
Eif3i	0.000747127	0.205566578	5
Bcl2l1	0.000755389	0.164230322	5
Ccdc25	0.000759368	0.187319	5
Ubc	0.000767187	0.158006578	5
Bmp1	0.000778765	0.202948988	5
Rab5c	0.000788399	0.209618573	5
Sf3b5	0.000790311	0.194697479	5
Eif3a	0.000800195	0.200919474	5
Zadh2	0.000810201	0.195070355	5
Ppp2cb	0.000835928	0.204521304	5
Esd	0.000841846	0.193550746	5
Mtmr1	0.000856743	0.202621364	5

Cfl1	0.000876132	0.173880703	5
Rgs16	0.000876513	0.194490796	5
Banf1	0.000899632	0.203068328	5
Capn10	0.000924048	0.185007561	5
Fkrp	0.000925446	0.127806597	5
Gc	0.000935329	0.170321144	5
Usp25	0.000940223	0.158212143	5
Magi3	0.00096649	0.15775746	5
Serinc2	0.000974302	0.16692961	5
Cyhr1	0.000977734	0.221066519	5
Prrc2a	0.000995094	0.221587211	5
Otud5	0.00099819	0.186430521	5
Dsp	0.001003753	0.230990782	5
Uhrf1bp1l	0.001012552	0.185842853	5
Atxn7l3	0.001013208	0.169791438	5
Abtb2	0.001018818	0.162292664	5
Pdia4	0.001026777	0.176063537	5
Tomm40	0.001075084	0.190430313	5
Kpna1	0.001075897	0.210720301	5
Pla2g12b	0.001078763	0.181373077	5
Idh3a	0.001098334	0.1864145	5
Myo5b	0.001116653	0.159171059	5
Ranbp2	0.001119769	0.213429714	5
Rab2a	0.001130486	0.207008854	5
Nop53	0.001149495	0.18688803	5
Fam102a	0.001159745	0.164241415	5
Tlcd1	0.001160557	0.184303716	5
Mllt6	0.001165564	0.171999944	5
Fibp	0.001170513	0.164137527	5
Rhot2	0.001174368	0.180613061	5
Sec16b	0.00117797	0.19856172	5
Dixdc1	0.001192469	0.158374165	5
Manba	0.001195796	0.16577051	5
Dnajb1	0.001206591	0.148952679	5
Slc30a1	0.001207385	0.203944974	5
Rnf6	0.001215756	0.173893343	5
Mbd1	0.001228547	0.169319395	5
Ap1m1	0.0012308	0.208901446	5
Mdm2	0.001233369	0.22714413	5
Creb3l3	0.001249369	0.166913458	5
Xpr1	0.001264209	0.169958713	5
Mri1	0.001297494	0.202898788	5
R3hdm2	0.001300382	0.179316023	5
Pcnx3	0.001301747	0.196321122	5
Rpn2	0.001305885	0.185973024	5
Shprh	0.001315444	0.157252389	5
Ecpas	0.001325333	0.191377337	5
Cers2	0.001370227	0.191868249	5
Mpnd	0.001370991	0.184362014	5
Derl1	0.001379686	0.200518095	5
Sos1	0.001381287	0.18210015	5
Mtmr10	0.001383475	0.212195717	5
Ddb1	0.001383601	0.194432373	5
H1f0	0.001399227	0.182352689	5
Thtpa	0.001413457	0.165352237	5
D8Ertd738e	0.001436183	0.204999766	5
Dpm3	0.001458276	0.20040929	5
Commd3	0.001460893	0.201482233	5
Strbp	0.001474024	0.197736236	5

Fmc1	0.001481008	0.18798883	5
Tial1	0.0015134	0.183185505	5
Trmt1	0.001523946	0.17097404	5
Sin3b	0.001528688	0.194337155	5
Slc17a5	0.001549162	0.208080415	5
Pepd	0.001555674	0.184963774	5
Idnk	0.0015645	0.207856922	5
Tkfc	0.001585094	0.15276615	5
Pcyt1a	0.001653595	0.179210362	5
Adipor1	0.001664294	0.190458405	5
Fbxw9	0.001675161	0.187631394	5
Fam126b	0.001686862	0.171728742	5
Cops3	0.001707687	0.208068012	5
Psmc5	0.00171282	0.212336695	5
Tor1aip2	0.001727018	0.206711316	5
Clptm1l	0.00173865	0.215409231	5
Inf2	0.001766633	0.207825991	5
Pex2	0.001770796	0.181189474	5
Psmg3	0.001787229	0.16208233	5
Prelid1	0.001791325	0.191962877	5
Irf3	0.001806341	0.206583219	5
Lztr1	0.001834885	0.199001329	5
Trim8	0.001848743	0.163255996	5
Rapgef1	0.001857701	0.200179371	5
Oxa1l	0.001871866	0.188762354	5
Diaph1	0.001877344	0.231058863	5
Cd59a	0.001883733	0.189948711	5
Arpc5l	0.001906447	0.189391581	5
Hcfc1r1	0.001923415	0.196118252	5
Zfyve21	0.00194563	0.18603947	5
Sec63	0.001963014	0.203141458	5
Hnrnpm	0.001964148	0.205294375	5
Rabep1	0.001979855	0.181910645	5
Casp9	0.001980123	0.128346343	5
Ermp1	0.001984411	0.173073857	5
Klf9	0.002002856	0.230971009	5
Fam114a2	0.002013909	0.200807944	5
Sympk	0.002014511	0.177887301	5
Gpr108	0.002017651	0.176891371	5
Cndp2	0.002030329	0.183044108	5
Mdfic	0.002033241	0.171010405	5
Ankrd52	0.002054615	0.193393028	5
Tmf1	0.002055225	0.174975756	5
Ttll4	0.002071471	0.148125209	5
Phf3	0.002086988	0.190823117	5
Zdhhc6	0.002126233	0.174156498	5
Mecr	0.002141882	0.188876114	5
Wdr45	0.002195285	0.165380045	5
Prpf18	0.002196348	0.205066894	5
0610012G03Rik	0.002197943	0.17783498	5
Hykk	0.002246287	0.218589586	5
Zdhhc7	0.002251103	0.170266688	5
Rabggtb	0.002256799	0.199860051	5
Hes6	0.002309645	0.199664855	5
Aptx	0.002310908	0.126427918	5
Mzt2	0.002314172	0.185463663	5
Ndufaf2	0.002321914	0.188040949	5
Pex26	0.002348595	0.184729405	5
Keg1	0.00237697	0.172621035	5

Ypel5	0.002387115	0.195824928	5
Zfp655	0.00241511	0.188984189	5
Dtd2	0.002416248	0.17051894	5
Prodh2	0.002428819	0.206895848	5
Pgghg	0.002429486	0.166607199	5
Usp4	0.00243826	0.191196017	5
Mat2a	0.002463828	0.194286665	5
Cisd3	0.0024999	0.18469181	5
L3mbtl2	0.002537695	0.164428801	5
Slc39a7	0.002539043	0.17839511	5
Ehmt1	0.002583788	0.157817978	5
Nfia	0.002614417	0.188157508	5
Il15ra	0.002626612	0.209259517	5
Zfp266	0.002636725	0.174807159	5
Rars	0.002638523	0.175260569	5
C1qbp	0.00264999	0.179978848	5
Skiv2l	0.002713561	0.221938651	5
Slc35b2	0.002724364	0.191764618	5
Iars	0.002727306	0.168307609	5
Dnm2	0.00277503	0.212808717	5
Kars	0.002829035	0.177225437	5
Pak2	0.002916918	0.171487836	5
Fnip2	0.002922081	0.167375466	5
Fto	0.003036703	0.190734672	5
Tmem131	0.003085304	0.179315405	5
Klhdc7a	0.003086015	0.149223614	5
Atat1	0.003088227	0.17431556	5
Ddx23	0.003107179	0.196430423	5
Srp9	0.00312659	0.203437632	5
Cul1	0.003136012	0.201630188	5
Rprd1a	0.003151513	0.168046052	5
Spsb2	0.003199536	0.148448945	5
Anxa11	0.003207162	0.185516948	5
Tmem259	0.003208199	0.20174767	5
Clta	0.003220776	0.178113818	5
Mob2	0.003256088	0.173673752	5
Sec62	0.003301835	0.183413603	5
Prdx2	0.00332665	0.226248703	5
Grpel2	0.003333335	0.182797885	5
Arl1	0.003352603	0.17588502	5
Bri3bp	0.003404361	0.191031076	5
Surf1	0.003433229	0.204954521	5
Plekha6	0.003468248	0.172752927	5
Cdip1	0.003546056	0.197837723	5
Atp6v1e1	0.003553194	0.163278874	5
Slc25a46	0.003612923	0.172106382	5
Afdn	0.003644518	0.184103639	5
Usp14	0.003670676	0.204160541	5
Sp1	0.003682545	0.165796652	5
Mvk	0.003765621	0.178573862	5
Ndufs4	0.003773287	0.163771661	5
Masp2	0.003789066	0.160809906	5
Wwp1	0.003832711	0.152471333	5
Gk	0.003850199	0.189566993	5
Nek7	0.0038522	0.192870662	5
2310039H08Rik	0.003863146	0.194777662	5
Spag7	0.003880176	0.18120231	5
Naa60	0.003909435	0.202980303	5
Edf1	0.003948937	0.197958366	5

Paics	0.003951364	0.151246001	5
Tulp4	0.003974047	0.17370333	5
Tcta	0.003992034	0.169602915	5
Tsen34	0.004040809	0.183040607	5
Csnk2b	0.004052866	0.189128068	5
Tnrc6a	0.004060979	0.191740224	5
Lcat	0.004062901	0.136225137	5
Pdxdc1	0.004063074	0.185486819	5
Rab9	0.004084626	0.196489049	5
Tef	0.004120698	0.169469697	5
Gpaa1	0.004178566	0.163950554	5
Litaf	0.004185309	0.167063334	5
Ube3a	0.004195536	0.191096045	5
Dph1	0.00419772	0.180164908	5
Rnf152	0.004237492	0.142615723	5
Asna1	0.004300636	0.182497848	5
Baat	0.004340569	0.175470299	5
Erh	0.004387296	0.176087235	5
Ndufa7	0.004411895	0.18604975	5
Ash1l	0.004504033	0.193932611	5
Cnot1	0.004504597	0.181866982	5
Tram1	0.004623698	0.145328354	5
Eif3h	0.004682632	0.198643005	5
Tbc1d13	0.004730451	0.184436883	5
Rab18	0.004776148	0.190026849	5
Zfand3	0.004885735	0.19804689	5
Clcn3	0.004886304	0.18134705	5
Flii	0.004923872	0.220521836	5
Bud31	0.004944127	0.18910305	5
Ppp4c	0.00495839	0.20073572	5
Tmed2	0.005057336	0.192280002	5
9530068E07Rik	0.005142534	0.187165139	5
Cct2	0.005165402	0.174745676	5
Bpgm	0.005187237	0.143707622	5
Twsg1	0.005264316	0.182523829	5
Stub1	0.005269067	0.204506555	5
Polr1c	0.005309598	0.173538317	5
Fem1a	0.005318447	0.146998315	5
Kcmf1	0.005337971	0.186191704	5
Drg1	0.005339151	0.180342662	5
Dcun1d4	0.005373251	0.180780457	5
Mapre3	0.005416403	0.174755702	5
Mkln1	0.005475624	0.193917048	5
Myorg	0.005498297	0.159923983	5
Rras2	0.005522912	0.175206108	5
Snrpd3	0.005625242	0.168036261	5
Ctnnd1	0.005654543	0.188335966	5
Anapc2	0.005789946	0.181495714	5
Safb	0.005800677	0.171437042	5
Nudt3	0.005803437	0.18882602	5
Stk25	0.005808459	0.199901781	5
Myh14	0.005895218	0.139095383	5
Grb2	0.005895956	0.178964332	5
Cmtm8	0.005935101	0.203104682	5
Pura	0.005954057	0.171570923	5
Nucb1	0.006029835	0.191527112	5
Arid5b	0.006142636	0.257185093	5
Rragc	0.006193449	0.175944485	5
Gps1	0.006221643	0.196417452	5

Cpsf3	0.006298389	0.17085705	5
Usp5	0.006348606	0.224917609	5
Tmem33	0.006368885	0.192356003	5
Ppp2ca	0.006397205	0.16082905	5
Hmox2	0.006398053	0.194261924	5
Vars2	0.006424925	0.1791482	5
Nelfb	0.006436743	0.168660857	5
Rbl2	0.00649631	0.176797257	5
Gigyf1	0.006507926	0.169426183	5
Plaa	0.006549776	0.166802911	5
Cldn3	0.006560722	0.175312678	5
Eif3g	0.006597122	0.175776297	5
Tgds	0.006626476	0.15322181	5
Eif4ebp1	0.006654304	0.198435896	5
Dld	0.006671845	0.18857311	5
Tmem11	0.006686219	0.170851752	5
Pfdn1	0.006836037	0.183673472	5
Nln	0.006874003	0.188700008	5
Fam174a	0.006916381	0.16224202	5
Plcg1	0.006977223	0.130666867	5
Lcor	0.006986551	0.157907698	5
Parp9	0.007076244	0.191028047	5
2900026A02Rik	0.007162182	0.196578477	5
Pi4kb	0.007173572	0.179743935	5
Nmt1	0.007296067	0.167657559	5
Fis1	0.007442498	0.165423858	5
Mfsd5	0.007447681	0.168154681	5
Aarsd1	0.007456353	0.184482026	5
Kdm4a	0.007469006	0.177989881	5
Foxo1	0.0074713	0.207335482	5
Atxn7l3b	0.007526193	0.166355319	5
Pcbp2	0.007597693	0.150617841	5
Snu13	0.007662414	0.184938155	5
Hnrnpul1	0.007732088	0.195003638	5
Atg101	0.00782083	0.200769248	5
Ndufs8	0.007826056	0.143654345	5
Arpc1a	0.007898462	0.178133705	5
Ltn1	0.007915284	0.200567384	5
Top1mt	0.007928954	0.153782298	5
Nek4	0.008016505	0.134475132	5
Cyp7b1	0.008063459	0.192635232	5
Cep350	0.008074865	0.157143648	5
Paip1	0.008081234	0.184150055	5
Spg7	0.008157671	0.185886747	5
Nrep	0.008223209	0.18699299	5
Tmem53	0.008249707	0.179194908	5
Vegfa	0.008292797	0.176507383	5
Lsm6	0.008328785	0.186890922	5
Dnajc21	0.008350031	0.183636352	5
Ctsh	0.008379793	0.156493094	5
Tfam	0.008469323	0.198049235	5
Carhsp1	0.00849641	0.178425767	5
Usp38	0.008524759	0.183948101	5
Tnpo3	0.008641801	0.168011015	5
Raf1	0.008767788	0.170946473	5
Ell2	0.008849305	0.202723433	5
Shc1	0.008945968	0.185229111	5
Heca	0.008965521	0.172447535	5
Mogs	0.009144518	0.193599709	5

Ebag9	0.009146944	0.132553123	5
Dock7	0.009149461	0.12191433	5
Mfn1	0.009152149	0.17767099	5
Pdzd8	0.009511883	0.179804938	5
Atp6v1c1	0.00957509	0.207238946	5
Ninj1	0.009599071	0.161127574	5
Gpr180	0.009672283	0.184181776	5
Bfar	0.009739148	0.192274243	5
Cdc123	0.00990004	0.18367053	5
Ncor2	0.009945952	0.216787668	5
Zfp91	0.009948472	0.191827464	5
Stoml2	0.009990211	0.169746295	5
Pcyox1	0.010190141	0.168839003	5
Tbl1xr1	0.010302111	0.204709856	5
Timm23	0.010305029	0.190623075	5
Slc25a28	0.010309503	0.178663583	5
Deptor	0.010332348	0.186356126	5
Wdr81	0.010558476	0.179234588	5
Cul3	0.010756583	0.168830712	5
Mllt10	0.01103748	0.14389517	5
Marf1	0.011122935	0.160802982	5
Ddc	0.011189694	0.212219218	5
Smim14	0.01121187	0.184357019	5
Dido1	0.011388987	0.181932419	5
Hs3st3b1	0.011652017	0.199935543	5
Hexa	0.01171151	0.184646963	5
Tbc1d20	0.011748659	0.214913438	5
Shank2	0.011762116	0.135150682	5
Mapkap1	0.011857705	0.170176461	5
Rnpep	0.011908236	0.182769326	5
Nek6	0.012104473	0.189423835	5
Mtch1	0.012214022	0.146265477	5
Gpn3	0.012305665	0.151471443	5
Srsf4	0.012452213	0.199194537	5
Atf4	0.012483902	0.20592417	5
Tfe3	0.012581483	0.133034685	5
Ubqln2	0.012638249	0.192827168	5
Dpp4	0.012759237	0.18750526	5
Exosc5	0.012779405	0.196493758	5
Coq5	0.012821706	0.187216251	5
Rin3	0.012853318	0.149133526	5
Fn1	0.012930298	0.141318298	5
Sfr1	0.013042953	0.197998174	5
Elavl1	0.013075042	0.177749749	5
Wdr73	0.013200947	0.148047124	5
Cxxc5	0.01320653	0.171580791	5
Zfand6	0.013303433	0.198827553	5
Pqbp1	0.013313718	0.172906231	5
Ppp6r3	0.013444399	0.169051319	5
Ptbp1	0.013617746	0.174576106	5
Hook1	0.013649503	0.144392892	5
Cyp2r1	0.013657515	0.158557202	5
Zkscan17	0.013798517	0.173830335	5
Tollip	0.013813993	0.201646811	5
Dhrs7b	0.01434926	0.141522474	5
Arhgef11	0.014357922	0.169408661	5
Dicer1	0.014402185	0.166528109	5
H3f3b	0.014485354	0.154426567	5
Fam160b1	0.014623646	0.158408793	5

Kremen1	0.014856297	0.131048382	5
Hp1bp3	0.014926044	0.173434226	5
Nrip1	0.015161026	0.133758002	5
Smarcd2	0.015348549	0.177646113	5
Acox3	0.015498269	0.178122039	5
Dapk3	0.015503895	0.163110381	5
Rnf130	0.015540467	0.209363659	5
Habp4	0.015809188	0.177177911	5
Tor2a	0.015829111	0.185510562	5
Mtor	0.015980753	0.172447535	5
Dhx37	0.016030761	0.159371404	5
Sacm1l	0.016050705	0.211893503	5
Ptpra	0.016091662	0.178827307	5
Oard1	0.016101792	0.169971395	5
Slc30a4	0.016174624	0.132233331	5
Anapc5	0.016370964	0.152192232	5
Epb41	0.016437959	0.197686783	5
Cep68	0.016541676	0.143640421	5
Nipsnap3b	0.016726534	0.211442348	5
Tsc22d2	0.016829787	0.130770111	5
Ttc7	0.016985959	0.158047121	5
Denr	0.017090414	0.193060775	5
Borcs8	0.017457771	0.172641684	5
Usp48	0.017460134	0.168961921	5
Ipo8	0.017778837	0.180662388	5
Rp9	0.017851099	0.183804735	5
Bub3	0.017984681	0.151742431	5
Tmem234	0.018049943	0.189489323	5
Miga2	0.01812152	0.179291456	5
Gabpa	0.018140878	0.187431134	5
Lrrc8d	0.018247935	0.168927028	5
Ncl	0.01849945	0.167709525	5
Adar	0.018952832	0.171776346	5
Eif2b4	0.019198719	0.159469348	5
Cyp4f15	0.019213056	0.159041626	5
Cdc14b	0.019396761	0.170273907	5
Naglu	0.019695691	0.145324848	5
Flnb	0.019803476	0.137107832	5
Fxyd1	0.019853983	0.14390147	5
Enpp1	0.019929398	0.14483448	5
Dnmt3a	0.020296979	0.151966261	5
Dad1	0.020316492	0.201227913	5
Pop5	0.020556646	0.181328261	5
Trrap	0.020569011	0.141070258	5
Fam120a	0.020570145	0.189829613	5
Stat5a	0.020760351	0.156359552	5
Nap1l1	0.021088223	0.178361493	5
Syngr2	0.021089768	0.174770575	5
Ssr3	0.021413785	0.178018542	5
Trabd	0.021470605	0.18058223	5
Slc25a17	0.021774898	0.183836602	5
Ube2k	0.021814073	0.186714511	5
Dram2	0.021916691	0.200131539	5
Pgp	0.021993007	0.17324199	5
Pole4	0.022039075	0.180175194	5
Bag1	0.022062297	0.155358493	5
Blcap	0.022155321	0.133240128	5
Cldnd1	0.022201458	0.165403942	5
Sirt2	0.022798475	0.15187726	5

Ndufab1	0.022807583	0.178446506	5
Elp3	0.02287338	0.124061504	5
Emc7	0.022881746	0.189245579	5
Lmbrd2	0.022907409	0.182461879	5
Pdhb	0.022916597	0.168152956	5
Cox16	0.023215529	0.162946149	5
Eva1a	0.023389547	0.155183522	5
Zcchc14	0.023392657	0.167080519	5
Gosr1	0.023454398	0.155792862	5
Creb3	0.023506595	0.204759911	5
Alkbh3	0.0235069	0.179500422	5
Tmem242	0.023512421	0.207663288	5
Znrf1	0.023527091	0.186882606	5
Mvb12a	0.023620262	0.177854119	5
Rsl1d1	0.023624165	0.14984493	5
Ampd2	0.023851854	0.182386531	5
Lats1	0.023942319	0.174585103	5
Eml3	0.024128839	0.142290447	5
Abtb1	0.024138098	0.192567634	5
Foxa2	0.024310392	0.178100772	5
Cdk17	0.02438517	0.166840672	5
Sart1	0.024551636	0.154181015	5
Mterf3	0.024673261	0.1286222	5
Ppa2	0.024916011	0.16261204	5
Ces1e	0.025576207	0.195579465	5
Amt	0.025635729	0.158247783	5
Nudt4	0.025636943	0.159341107	5
Zhx3	0.025790224	0.187785521	5
Azin1	0.025805046	0.158530888	5
Thoc7	0.025852271	0.162381407	5
Fkbp15	0.025871203	0.171990633	5
F12	0.025881472	0.137264587	5
Cbx4	0.025992804	0.167119793	5
Rbck1	0.026260826	0.203559812	5
Hook3	0.026291984	0.171964409	5
Bsdc1	0.026373225	0.156920827	5
Afg3l1	0.026410014	0.19179568	5
Mbnl1	0.026576824	0.166945535	5
Arih1	0.026590668	0.205570615	5
Snx2	0.02696376	0.166856096	5
Mink1	0.027072577	0.15398715	5
Itfg1	0.02707594	0.168976142	5
Cuta	0.027100665	0.168080149	5
Ndufv2	0.027118438	0.170384912	5
1700123O20Rik	0.027300909	0.173152219	5
BC029722	0.027435576	0.167137688	5
Slain2	0.027555289	0.163798795	5
Bach1	0.027591129	0.174479038	5
Xpo6	0.02766214	0.170932503	5
Sugct	0.028235114	0.222072413	5
Prpsap1	0.028469554	0.178458818	5
Cited2	0.028533348	0.164389366	5
Dnajc25	0.02873764	0.184939838	5
Pex3	0.028823994	0.188772935	5
Xiap	0.028862226	0.199193796	5
Mmab	0.028870243	0.151067502	5
Sppl2a	0.028912285	0.158160698	5
Stk40	0.028915948	0.174590809	5
Strn4	0.02921753	0.185427851	5

Abca1	0.029218511	0.175551063	5
Pomt1	0.029335089	0.149042828	5
Nckap1	0.029634857	0.160760881	5
Yif1a	0.029765864	0.179101684	5
Tpr	0.029787435	0.17671825	5
Myo18a	0.030107824	0.186976044	5
Sipa1l1	0.030628769	0.120166969	5
Coa3	0.030726089	0.184214272	5
Phlpp1	0.030935565	0.203598986	5
Ctso	0.031352408	0.19668672	5
Hnrnpa2b1	0.031379239	0.166728482	5
Slc35b4	0.031493166	0.151182847	5
Dmac1	0.031672232	0.172812544	5
Os9	0.031690402	0.166469211	5
Smad3	0.031698945	0.125834698	5
Dolpp1	0.031819852	0.129664847	5
Dcun1d5	0.032101933	0.156051618	5
Kif21a	0.032491124	0.159346154	5
Dars2	0.03254451	0.128396694	5
Stradb	0.032648808	0.204693344	5
Alg1	0.032729451	0.163755546	5
Chmp6	0.032941229	0.15563887	5
Tmed4	0.033356739	0.15425976	5
Cd47	0.0339394	0.177211845	5
Zmpste24	0.034011859	0.175551365	5
Polr2b	0.034108539	0.187850156	5
Desi1	0.034214598	0.178889674	5
Daglb	0.034332469	0.184636944	5
Zfp637	0.034344458	0.143563648	5
Gpc4	0.034727924	0.180335902	5
Fig4	0.035212951	0.153040766	5
Cwc15	0.036063215	0.190792903	5
Yes1	0.036097963	0.163784327	5
Sars	0.036229768	0.172262012	5
Fuca1	0.036528841	0.201284391	5
Pycrl	0.036641432	0.164017937	5
Cct4	0.037000344	0.137963677	5
Pdss2	0.037004089	0.139510442	5
Ubn1	0.037008257	0.149509268	5
Zfp871	0.037024297	0.204012287	5
Etf1	0.03716394	0.163985863	5
Sbds	0.037171984	0.167172593	5
Rarres2	0.037379088	0.132597154	5
Nup153	0.037469719	0.168804466	5
Rbx1	0.037681965	0.156616537	5
Yipf4	0.037759092	0.169937044	5
Pip4k2c	0.038125241	0.16123189	5
Gorasp2	0.038463707	0.158639405	5
Aip	0.039058815	0.135873736	5
4933434E20Rik	0.039225963	0.1649378	5
Nt5c2	0.039492822	0.160704986	5
Aldh16a1	0.040197917	0.116299462	5
Plin5	0.040272566	0.13090456	5
Zkscan7	0.040405036	0.129594377	5
Vps4b	0.040689129	0.144343475	5
Tcea3	0.040899773	0.201272631	5
Ctnna1	0.041490318	0.148714238	5
Atp6v0a1	0.042397126	0.15772181	5
Vps16	0.042665637	0.135491135	5

Ppp6c	0.043083931	0.174173939	5
Usf3	0.043511383	0.181536613	5
Fam222b	0.043939376	0.163934056	5
Dexi	0.04456887	0.170617631	5
Letm1	0.045725625	0.155595999	5
Auh	0.045798866	0.172839252	5
Rcl1	0.045930879	0.178804658	5
Capn15	0.046191078	0.170527727	5
Clip1	0.04627705	0.177368758	5
Cdc42bpb	0.046437941	0.185515255	5
Wdr11	0.047112943	0.163244767	5
Cnn3	0.047773864	0.168604871	5
Cep85l	0.04792563	0.183713863	5
Arhgef10l	0.048527847	0.178013653	5
Mien1	0.048696269	0.171694439	5
Ubfd1	0.049285896	0.156217512	5
Cdc16	0.049817052	0.1672118	5
Gnai3	0.049880006	0.170777259	5
Spp1	1.41E-157	3.008228809	6
Mgp	4.75E-221	1.332984252	6
Gsn	2.03E-191	1.55206279	6
Dpt	1.19E-178	1.515219253	6
C7	5.04E-171	1.088519097	6
Scara3	5.10E-159	0.607931357	6
Acta2	2.39E-157	1.261235948	6
Dpep1	5.64E-155	0.579524804	6
Emp1	1.69E-153	0.63646214	6
Fbln2	9.86E-150	0.785628221	6
Fmod	4.82E-148	0.678558528	6
Crispld2	4.21E-131	0.450271577	6
Gja5	5.77E-130	0.529161241	6
Fbln1	6.45E-129	0.455956283	6
Atp1a2	4.08E-127	0.523063597	6
Eln	4.45E-127	1.341489603	6
Col1a1	2.22E-124	1.648073199	6
Epcam	5.64E-116	0.586660861	6
Lamc3	6.02E-113	0.451790809	6
Myh11	2.09E-112	0.676933507	6
Mmrn1	1.11E-111	0.471845662	6
S100a6	2.01E-111	1.04363537	6
Tagln	8.91E-111	1.517242942	6
Tspan8	4.32E-109	0.524659675	6
Prom1	4.78E-107	0.43603643	6
Gpx3	5.56E-106	1.153492347	6
Bcam	7.36E-104	0.685320482	6
Spon1	8.04E-98	0.440152539	6
Htra3	2.38E-97	0.609367813	6
Fbln5	2.94E-95	0.937113404	6
Mfap4	4.72E-95	0.515426905	6
Fmo2	4.93E-95	1.019862749	6
Col3a1	5.10E-95	1.940900258	6
Col6a2	5.40E-94	0.874265571	6
Bicc1	1.27E-92	0.670009165	6
Svep1	4.47E-92	0.434436813	6
Gas6	1.07E-87	1.72505525	6
Krt7	5.08E-85	0.389422712	6
Cav1	3.98E-81	0.594730592	6
Igfbp5	5.79E-81	0.517395011	6

Tmsb4x	3.23E-79	1.189912427	6
Ccn2	1.69E-78	0.977127417	6
Ehd2	7.77E-75	0.63764982	6
Sparcl1	2.20E-74	0.711561812	6
Col1a2	4.94E-74	1.45040886	6
Cavin3	1.93E-73	0.406705992	6
Loxl1	2.33E-73	0.384009216	6
Smoc2	5.43E-73	0.553581868	6
Myl9	5.43E-68	0.646166639	6
Cd74	1.35E-67	1.647178689	6
Apoa4	1.41E-66	1.005404128	6
Prss23	1.33E-64	0.837790537	6
Timp2	6.90E-64	1.029650901	6
Vim	1.46E-63	1.18066261	6
Cd34	2.03E-63	0.492060957	6
Timp3	1.17E-62	1.010283694	6
Ahnak	2.90E-62	1.063014056	6
Aldh1b1	1.39E-61	1.130730116	6
Crip1	4.95E-61	1.052137018	6
Col6a3	5.33E-60	0.733336965	6
Thbs1	4.05E-59	0.551369569	6
Adamts2	6.21E-59	0.677740046	6
Lama5	9.99E-59	0.432099741	6
Tnxb	1.82E-54	0.648557355	6
Mfge8	1.96E-54	0.762345884	6
Cst3	2.27E-53	1.170058914	6
Ly6e	2.57E-52	0.840108842	6
Mup20	3.52E-52	0.76971255	6
Adamtsl2	2.43E-51	0.341729645	6
H2-Aa	2.72E-51	1.577696655	6
Flna	4.46E-51	1.136948602	6
H2-Eb1	9.69E-51	1.396896192	6
App	9.76E-51	0.939902022	6
Cd24a	2.51E-50	0.363809262	6
Vwf	5.96E-50	0.837983609	6
S100a11	8.99E-50	0.767159734	6
Naaa	6.88E-47	0.876675292	6
Tagln2	2.13E-46	0.937623214	6
Rap1gap	1.07E-45	0.423588317	6
Adgrg6	1.25E-45	0.406703296	6
Slc25a4	3.28E-45	0.685971739	6
Anxa5	4.24E-45	0.956299637	6
Bgn	8.85E-45	1.024008975	6
Pkhd1	2.20E-44	0.49636818	6
Podn	2.06E-42	0.469584948	6
Igfbp3	5.40E-42	1.022033211	6
Ezr	7.86E-42	0.568115292	6
Hsd17b6	9.34E-42	0.820954272	6
Thbs2	1.30E-41	0.513551903	6
Fgl2	1.81E-41	0.733099004	6
Cryab	1.15E-40	0.560516752	6
Col6a1	1.32E-40	0.580663006	6
Myof	1.44E-39	0.338642629	6
Cyp2f2	4.18E-39	0.647345059	6
H2-Ab1	7.61E-39	1.458727041	6
Tm4sf4	9.04E-39	0.780232542	6
Adamts5	4.29E-38	0.348953422	6
Fbn1	5.26E-38	0.44165081	6
Scd2	6.06E-38	0.451607867	6

Fstl1	7.08E-38	0.432873496	6
Pdzk1ip1	3.85E-37	0.628946307	6
Sptbn1	4.30E-37	0.793185677	6
Col5a2	7.01E-37	0.469481811	6
Sparc	7.74E-37	0.908280749	6
Ndufa4l2	9.59E-37	0.375325749	6
Ccr2	1.00E-36	0.376693563	6
Des	1.25E-36	0.637182947	6
Tpm2	1.26E-36	0.855004728	6
Ltbp4	1.45E-36	0.810273971	6
Fblim1	4.46E-36	0.350084965	6
Tspan3	5.37E-36	0.50792498	6
Igfbp7	6.37E-36	0.757041777	6
Sds	1.61E-35	0.733352385	6
Thy1	1.64E-35	0.382758135	6
Sh3bgrl3	8.28E-35	0.900449608	6
Hsd17b13	1.09E-34	0.666343198	6
Galnt15	8.98E-34	0.47413195	6
Lsp1	1.94E-33	0.575334241	6
Serpinh1	2.78E-33	0.671924419	6
Heyl	3.35E-33	0.459908895	6
Tns1	3.83E-33	0.898337461	6
Atp1b1	4.23E-33	0.753798972	6
Ciita	1.11E-32	0.450125424	6
Gdf10	1.56E-32	0.530124718	6
Ptpn14	1.57E-32	0.381233893	6
Lamb2	2.13E-32	0.301171645	6
Tm4sf1	1.68E-31	0.841407462	6
Tstd1	4.69E-31	0.610382467	6
Apobec3	5.11E-31	0.468479117	6
Lhfp	9.70E-31	0.672710576	6
Anxa3	3.33E-30	0.623754884	6
Casp12	6.30E-30	0.375844092	6
Irf8	6.90E-30	0.679635371	6
Col4a1	7.08E-30	0.698363543	6
Cygb	7.46E-30	0.431010878	6
Fgfr1	1.47E-29	0.434818227	6
Ppp1r14a	4.44E-29	0.566593675	6
Anxa2	4.64E-29	0.644123455	6
Etnppl	2.12E-28	0.740010915	6
Sox9	2.53E-27	0.579404431	6
Ehd4	3.17E-27	0.550959928	6
Dtx3	6.24E-27	0.405757737	6
Ptgfrn	7.55E-27	0.297889964	6
Jag1	1.30E-26	0.510206324	6
Pamr1	1.45E-26	0.334281158	6
Cbfa2t3	8.76E-26	0.477369654	6
Pdgfa	1.44E-25	0.419829995	6
P3h3	1.57E-25	0.305062713	6
Cavin1	3.18E-25	0.329695833	6
Ednrb	4.46E-25	0.554099636	6
Synpo	6.69E-25	0.452388771	6
Pck1	7.41E-25	0.558150496	6
Ugt2b38	1.16E-24	0.628012361	6
Fam129b	1.38E-24	0.390729527	6
Gm13889	1.48E-24	0.391014532	6
Col14a1	1.84E-24	0.690339322	6
Anxa1	2.30E-24	0.384469149	6
Pfkp	8.75E-24	0.311831847	6

Clu	1.29E-23	0.454651005	6
Abhd2	4.48E-23	0.644991904	6
Clstn1	5.57E-23	0.389868562	6
Pcolce	6.75E-23	0.663556309	6
Ly6a	2.52E-22	0.303882938	6
Iqgap1	2.59E-22	0.460770208	6
Tpm1	2.90E-22	0.762844514	6
Itih5	3.25E-22	0.444106809	6
Kifc3	4.04E-22	0.630189948	6
Csrp1	7.48E-22	0.637942587	6
Ntn4	9.95E-22	0.512181361	6
Ptpn18	1.01E-21	0.433450682	6
Heg1	1.32E-21	0.585267069	6
H2-DMb1	2.14E-21	0.39315631	6
Gabbr1	7.18E-21	0.331050274	6
Plcb4	1.21E-20	0.332600801	6
Ucp2	1.27E-20	0.596607903	6
Gls	1.35E-20	0.490703981	6
Pdgfra	1.36E-20	0.30970848	6
Hpx	1.68E-20	0.469938657	6
Vegfc	3.61E-20	0.372444235	6
Txnip	3.98E-20	0.657862501	6
Lum	4.61E-20	0.521234742	6
Selenom	4.99E-20	0.304790579	6
Coro1a	7.88E-20	0.497590075	6
Bmyc	1.03E-19	0.394361762	6
Ppp1r9b	2.21E-19	0.526968038	6
Lbh	2.35E-19	0.398066611	6
Shroom3	2.65E-19	0.406051387	6
Myh9	2.95E-19	0.529605052	6
Fxyd5	5.06E-19	0.49518085	6
Id1	1.63E-18	0.538477708	6
Cdh1	1.98E-18	0.54165848	6
Jam2	2.44E-18	0.477920497	6
Pip4k2a	2.67E-18	0.488225271	6
Ngf	4.82E-18	0.35858999	6
Cavin2	6.03E-18	0.480052833	6
Acvrl1	6.47E-18	0.301786531	6
Cd93	7.85E-18	0.466429919	6
Marcks	8.09E-18	0.543289682	6
Gpc6	8.41E-18	0.367037146	6
Ppt1	1.68E-17	0.555533948	6
Gls2	1.76E-17	0.49458398	6
Actb	3.58E-17	0.522566891	6
Col4a2	3.65E-17	0.587448082	6
Utrn	5.28E-17	0.392734562	6
Myh10	8.34E-17	0.379339061	6
Jund	8.44E-17	0.489352597	6
Psap	9.10E-17	0.499412088	6
Cd52	1.40E-16	0.711617024	6
Lgals1	2.42E-16	0.430173829	6
Hal	2.46E-16	0.485090437	6
Pdgfrb	2.62E-16	0.285762158	6
Slc43a3	3.16E-16	0.545872998	6
Clec9a	3.87E-16	0.34533554	6
Mcam	6.91E-16	0.274601252	6
BC028528	7.19E-16	0.2839439	6
Adgrg1	7.48E-16	0.286021215	6
H2-DMa	7.86E-16	0.543095011	6

Itgb5	8.38E-16	0.554478964	6
Itga8	1.28E-15	0.269141092	6
Vegfd	1.79E-15	0.303718457	6
Zyx	2.42E-15	0.555569491	6
Pam	2.87E-15	0.539010463	6
Ccnd1	3.24E-15	0.520983097	6
Ifngr1	4.62E-15	0.450351711	6
Prelp	4.81E-15	0.577203997	6
Adam15	7.10E-15	0.295432034	6
Ptp4a3	1.01E-14	0.512020884	6
Cav2	1.06E-14	0.404853025	6
Pak1	1.08E-14	0.328761718	6
Gng11	1.88E-14	0.448096721	6
Igf1r	2.01E-14	0.245925284	6
Adamts10	2.10E-14	0.41836041	6
Plxnd1	2.56E-14	0.47115716	6
G6pc	3.40E-14	0.417612347	6
Nnmt	4.00E-14	0.408097413	6
Fbp1	6.60E-14	0.371679496	6
Rhbdf1	7.26E-14	0.306476499	6
Pigr	8.03E-14	0.396715623	6
She	1.07E-13	0.336019036	6
Tgm2	1.08E-13	0.533022678	6
Crim1	1.17E-13	0.417094895	6
Spry1	1.25E-13	0.382971507	6
Sdc1	1.49E-13	0.429338834	6
Clec2h	1.56E-13	0.414932554	6
Apoc2	1.80E-13	0.477516251	6
Arl4c	1.96E-13	0.32890134	6
Reep5	2.13E-13	0.443429743	6
Serpine2	2.41E-13	0.249785652	6
Npdc1	3.67E-13	0.294458451	6
Pear1	4.61E-13	0.344526098	6
Cdc42ep1	4.71E-13	0.445545698	6
Laptm5	5.66E-13	0.505393075	6
Podxl	6.25E-13	0.413616825	6
Socs5	6.60E-13	0.282222649	6
Dst	6.65E-13	0.330480089	6
Rcn3	8.47E-13	0.313171436	6
Cnn2	8.48E-13	0.470051563	6
Rhoj	9.44E-13	0.384362242	6
Slc3a1	1.10E-12	0.471327154	6
Lcn2	2.12E-12	0.234363355	6
Aldob	2.13E-12	0.334480382	6
Cmklr1	2.16E-12	0.32790827	6
Myo1c	2.38E-12	0.430508489	6
Adamts9	2.46E-12	0.293718118	6
Gja4	2.86E-12	0.349417222	6
Myadm	3.16E-12	0.378181018	6
Setd7	3.73E-12	0.322344099	6
Pea15a	3.80E-12	0.280073869	6
Sptan1	5.12E-12	0.477627546	6
Defb1	5.60E-12	0.303284663	6
Alcam	5.85E-12	0.417615477	6
Wls	5.93E-12	0.398844023	6
Fam129a	6.10E-12	0.264207443	6
Prex2	6.16E-12	0.457220442	6
Ptprc	6.20E-12	0.467098786	6
Agrn	6.90E-12	0.400459066	6

Tm6sf1	7.58E-12	0.245147594	6
Rgs10	7.80E-12	0.313068631	6
Kctd10	8.39E-12	0.332880257	6
Emp3	1.03E-11	0.337382209	6
Lamc1	1.03E-11	0.370609052	6
Nedd4	1.14E-11	0.397538528	6
Itgb2	1.27E-11	0.329356042	6
Hexb	1.32E-11	0.367771079	6
Tap2	1.44E-11	0.441982848	6
Tgfbr2	2.19E-11	0.401341637	6
Hk1	2.21E-11	0.315141369	6
Orm1	2.57E-11	0.336874353	6
Dab2ip	2.97E-11	0.342174196	6
Ccnd2	3.08E-11	0.351661678	6
Golm1	3.27E-11	0.234422409	6
Rgs3	3.67E-11	0.268098051	6
Gltp	3.74E-11	0.25044368	6
Zmiz1	4.22E-11	0.401172725	6
Amdhd1	4.28E-11	0.350595432	6
Tns3	4.53E-11	0.426735189	6
Fgfr3	4.84E-11	0.489986027	6
Arhgef2	7.19E-11	0.310855361	6
Cystm1	7.87E-11	0.36619809	6
Aldoa	1.01E-10	0.43391531	6
Gucy1a2	1.14E-10	0.242706374	6
Capns1	1.17E-10	0.362426601	6
Efhd2	1.26E-10	0.475395394	6
Gbp4	1.33E-10	0.28111016	6
Ift122	1.39E-10	0.232444299	6
Itm2c	1.44E-10	0.441683184	6
St3gal4	1.49E-10	0.406397706	6
Rapgef5	1.52E-10	0.372575632	6
Cryl1	1.76E-10	0.351677329	6
Pbx1	2.02E-10	0.385033236	6
Sfxn1	2.05E-10	0.418393113	6
Acly	2.24E-10	0.372102135	6
Ski	3.04E-10	0.354835809	6
Atp1a1	3.40E-10	0.380502289	6
Gnai2	3.90E-10	0.431290058	6
Evl	4.77E-10	0.276343051	6
Zeb2	5.17E-10	0.380392949	6
Clic1	5.18E-10	0.450497785	6
Jcad	5.40E-10	0.299197512	6
Ctsc	5.70E-10	0.389435829	6
Trim47	5.97E-10	0.251870028	6
Anxa6	7.60E-10	0.430553586	6
Ptprs	7.76E-10	0.215298323	6
Hcls1	9.21E-10	0.303640689	6
Arhgdia	9.55E-10	0.410339248	6
Atp11a	9.65E-10	0.260995971	6
Plekhg2	1.32E-09	0.274269368	6
Ccdc80	1.42E-09	0.428262169	6
Slc9a3r2	1.57E-09	0.454951266	6
Klf6	1.73E-09	0.335867878	6
Pdlim7	1.82E-09	0.319135634	6
Pld1	1.83E-09	0.346647812	6
Itpripl2	1.95E-09	0.277424868	6
Bdh2	2.29E-09	0.350426941	6
Lgals3	2.38E-09	0.367189723	6

Cd9	2.44E-09	0.355888723	6
Plec	2.61E-09	0.418460653	6
Osmr	3.71E-09	0.260888582	6
Dock10	4.24E-09	0.290028445	6
Got1	4.33E-09	0.360640883	6
Gm2a	4.59E-09	0.391832461	6
Filip1l	4.83E-09	0.288349655	6
Anxa4	5.44E-09	0.475780898	6
Lims1	5.52E-09	0.271845708	6
Sult1a1	6.36E-09	0.328853548	6
Alox5ap	6.45E-09	0.269454243	6
Cotl1	8.26E-09	0.392451335	6
Prrc2c	8.57E-09	0.368405906	6
Tlr3	9.04E-09	0.266671843	6
Wasf2	9.17E-09	0.374287711	6
Tln1	1.01E-08	0.424470343	6
Slco3a1	1.05E-08	0.254071806	6
Ccn1	1.25E-08	0.278771604	6
Cyba	1.27E-08	0.412334962	6
Slc6a6	1.28E-08	0.386214503	6
Serinc3	1.31E-08	0.339287822	6
Sod3	1.39E-08	0.467288922	6
Nid1	1.55E-08	0.32761154	6
Rgs2	1.66E-08	0.306109087	6
Lmo2	1.91E-08	0.267609427	6
Arrb1	1.96E-08	0.268343729	6
Pakap.1	2.27E-08	0.287875804	6
Arhgap6	2.49E-08	0.274195618	6
Gucy1a1	2.55E-08	0.27592404	6
Mmrn2	2.71E-08	0.34389407	6
Ctnna1	2.86E-08	0.343474205	6
Stx7	3.15E-08	0.328961185	6
Add1	3.30E-08	0.309467198	6
Ddit4	3.56E-08	0.259373995	6
Fzd1	4.03E-08	0.266163412	6
H2-D1	4.23E-08	0.341941499	6
Mprip	4.30E-08	0.359738467	6
Patz1	5.35E-08	0.258028125	6
Cib3	6.08E-08	0.295175367	6
Puf60	6.26E-08	0.325507974	6
Psmb9	7.57E-08	0.403385069	6
Ptprm	7.57E-08	0.275878506	6
Slc44a2	7.65E-08	0.304451013	6
Selplg	8.10E-08	0.260907972	6
Dek	8.11E-08	0.371770747	6
Prkar1a	8.84E-08	0.353080546	6
Oxct1	1.03E-07	0.262484046	6
H3f3b	1.07E-07	0.372358763	6
Dpysl2	1.11E-07	0.27812359	6
Nenf	1.20E-07	0.370268615	6
Prnp	1.22E-07	0.314001456	6
Cd53	1.23E-07	0.315422131	6
Ctsb	1.41E-07	0.319722268	6
Hnrnpa1	1.43E-07	0.36530043	6
Col5a1	1.56E-07	0.237134191	6
Gbp2	1.91E-07	0.194252387	6
Efemp2	2.10E-07	0.248499135	6
Gnb2	2.16E-07	0.331178109	6
Psmb8	2.21E-07	0.407128446	6

Map3k1	2.30E-07	0.395229532	6
Pck2	2.43E-07	0.269697631	6
Arpc1b	2.61E-07	0.404218478	6
Fgfr2	2.63E-07	0.327045609	6
Wsb1	2.67E-07	0.338944854	6
Map4	2.69E-07	0.347282403	6
Ddx5	2.86E-07	0.333922068	6
Vasp	3.10E-07	0.360651385	6
Ywhaz	3.11E-07	0.331216484	6
Mbnl1	3.43E-07	0.36561221	6
Ano6	3.74E-07	0.245894936	6
Abcc5	4.28E-07	0.232909605	6
Fnbp1	4.33E-07	0.333291464	6
Hbb-bt	4.51E-07	0.928174256	6
Dlgap4	4.64E-07	0.345553221	6
Nap1l1	4.67E-07	0.384988864	6
Angptl2	4.96E-07	0.218198932	6
Spag9	4.99E-07	0.348481693	6
F11r	5.07E-07	0.352386573	6
Egfl7	5.18E-07	0.391729062	6
Tdo2	5.32E-07	0.298626593	6
Prrc2b	5.50E-07	0.326191911	6
Msn	6.38E-07	0.393082429	6
Srgap2	6.86E-07	0.332490718	6
Lama4	7.22E-07	0.247577457	6
Smim22	7.29E-07	0.255994424	6
Fkbp1a	7.73E-07	0.331011963	6
Adgre5	8.10E-07	0.29386689	6
Esyt1	8.48E-07	0.291760865	6
Pbxip1	8.55E-07	0.297680549	6
Actr3	1.06E-06	0.345679952	6
Myl12a	1.20E-06	0.378325004	6
Tmem173	1.31E-06	0.222691635	6
Plekho1	1.46E-06	0.318919575	6
Cd36	1.64E-06	0.350539909	6
Thra	1.72E-06	0.247851343	6
Gpsm3	1.80E-06	0.248859108	6
Dsg2	1.93E-06	0.36138539	6
Plekho2	2.00E-06	0.316870863	6
Pik3ap1	2.06E-06	0.376342711	6
Apoc3	2.20E-06	0.258932203	6
Laptm4a	2.39E-06	0.335730996	6
Tead1	2.49E-06	0.210773534	6
Cap1	2.52E-06	0.338663511	6
Serpina12	2.61E-06	0.460629837	6
Entpd1	2.62E-06	0.283573265	6
Map4k4	2.66E-06	0.24978233	6
Sorbs2	2.86E-06	0.344694732	6
Asl	2.87E-06	0.279633517	6
Exoc6	2.89E-06	0.280202218	6
Rtn4	2.91E-06	0.309946097	6
Plac8	2.91E-06	0.376026376	6
Brd8	3.18E-06	0.274089366	6
Nfib	3.37E-06	0.342495721	6
Apoa5	3.47E-06	0.259256563	6
Ptgr1	3.88E-06	0.230133344	6
Ivns1abp	4.13E-06	0.344998711	6
Tcf7l1	4.26E-06	0.253361163	6
P4ha1	4.57E-06	0.229692257	6

Hnrnpul1	5.02E-06	0.32057867	6
Tmem51	5.30E-06	0.233798402	6
Gcnt2	5.33E-06	0.286607825	6
Cfl1	5.50E-06	0.325764075	6
Samhd1	5.52E-06	0.278368888	6
Tnfrsf12a	5.62E-06	0.247399952	6
Calu	5.63E-06	0.321095339	6
Unc93b1	5.74E-06	0.35675606	6
Mylk	5.91E-06	0.362401059	6
Camk2d	5.96E-06	0.280070636	6
Hnrnpa0	6.06E-06	0.290579287	6
Snx1	6.16E-06	0.282500742	6
Ehbp1l1	6.42E-06	0.247959163	6
Lrrc32	6.48E-06	0.295828402	6
Son	6.54E-06	0.312098426	6
Mcl1	6.73E-06	0.327218255	6
Cdk19	7.00E-06	0.265883858	6
Epb41l2	7.19E-06	0.30387799	6
Tspan7	7.28E-06	0.298984923	6
Vgll4	7.50E-06	0.308393152	6
S100a16	7.61E-06	0.3106878	6
Rida	7.63E-06	0.297165962	6
Gnas	8.18E-06	0.308083838	6
Plekhb2	8.96E-06	0.192528162	6
Pak2	9.06E-06	0.29010155	6
Lamb1	9.19E-06	0.24074039	6
Kifap3	9.31E-06	0.215314073	6
Trim30a	9.68E-06	0.271583995	6
Cp	1.01E-05	0.270292244	6
Atoh8	1.05E-05	0.330009667	6
Sfpq	1.05E-05	0.313676583	6
Gas2	1.11E-05	0.336394302	6
Jup	1.14E-05	0.32859752	6
Sh2b3	1.16E-05	0.29299295	6
H2-Q6	1.23E-05	0.290309948	6
Rtl8b	1.25E-05	0.264034808	6
Hnrnpl	1.29E-05	0.31966975	6
Tshz2	1.42E-05	0.33823953	6
Efnb1	1.43E-05	0.287458807	6
Map3k20	1.46E-05	0.194321108	6
Tuba1a	1.50E-05	0.251420547	6
Rgs5	1.53E-05	0.607527408	6
Hnf1b	1.56E-05	0.252753002	6
Plscr3	1.60E-05	0.237450175	6
Actn1	1.60E-05	0.360671897	6
Rbm39	1.60E-05	0.284528123	6
Rsu1	1.72E-05	0.301165051	6
Eppk1	1.91E-05	0.278595988	6
Sri	1.93E-05	0.338269804	6
Ppic	1.98E-05	0.221927443	6
Cxcl12	1.99E-05	0.359237858	6
Cbs	2.08E-05	0.287383421	6
Ankfy1	2.15E-05	0.247599933	6
Esyt2	2.20E-05	0.314763115	6
Cyp39a1	2.24E-05	0.323602684	6
Cmtm7	2.31E-05	0.236075787	6
Fam117b	2.37E-05	0.230557171	6
Bmp6	2.41E-05	0.30716977	6
Tent5c	2.42E-05	0.259754309	6

Npc2	2.65E-05	0.312396606	6
Nipbl	2.74E-05	0.291428498	6
Adgra2	3.03E-05	0.227362024	6
Nr1d1	3.04E-05	0.341820947	6
Rasgef1b	3.10E-05	0.261048639	6
Inpp5d	3.37E-05	0.256362147	6
Shisa5	3.47E-05	0.250121506	6
Ralb	3.62E-05	0.228179734	6
Ifi27l2a	3.92E-05	0.304150082	6
Epas1	4.08E-05	0.323757808	6
Spint2	4.09E-05	0.332059285	6
Fes	4.23E-05	0.238727835	6
Ptar1	4.26E-05	0.217654621	6
Cadm1	4.45E-05	0.342676194	6
Galnt10	4.51E-05	0.189429906	6
Fnbp1l	4.59E-05	0.240258133	6
Ctss	4.65E-05	0.342133495	6
Adgrf5	5.01E-05	0.332447501	6
Ralgapa1	5.34E-05	0.301699271	6
Gnb1	5.35E-05	0.339464673	6
Eid1	5.51E-05	0.321994785	6
Pabpc1	5.53E-05	0.278812361	6
Fam3c	5.91E-05	0.308110798	6
Fst	6.03E-05	0.211354131	6
Vasn	6.06E-05	0.261500878	6
Csnk1a1	6.17E-05	0.300155374	6
Gas1	7.00E-05	0.215604433	6
Tsc22d4	7.47E-05	0.302713115	6
Map4k3	8.25E-05	0.280570179	6
Cldn5	8.30E-05	0.260030131	6
Foxp1	8.32E-05	0.290760077	6
Irf5	8.33E-05	0.272993876	6
Camk1	8.71E-05	0.291888122	6
Lmo4	9.03E-05	0.287019634	6
Amotl1	9.80E-05	0.273846061	6
Pan3	0.000100257	0.240246692	6
Isyna1	0.000100373	0.182164869	6
Fgd5	0.000101003	0.313717825	6
Pde4b	0.00010212	0.206431295	6
Mpeg1	0.000102151	0.409616677	6
Ccl5	0.000106787	0.319164533	6
Btg1	0.000111792	0.298348618	6
Tax1bp3	0.000118384	0.206273033	6
Vcam1	0.000121667	0.240378549	6
Aspg	0.000125185	0.265787011	6
Anapc5	0.000126808	0.326095154	6
Wwtr1	0.000131391	0.283206048	6
Ablim1	0.000135132	0.237097167	6
Sntb2	0.000135133	0.1959869	6
Arvcf	0.000137205	0.237419619	6
Sirpa	0.000146092	0.318185033	6
Atxn2l	0.000147064	0.310962819	6
Rsrp1	0.00015426	0.264490387	6
Ilk	0.000160529	0.268678825	6
Mxra8	0.000161078	0.215315771	6
Wdr1	0.000165694	0.296228566	6
Dock6	0.000168728	0.296890148	6
Dennd3	0.000200239	0.198299316	6
Stxbp1	0.000222686	0.165728438	6

Irf2bp2	0.000228553	0.328625741	6
Emilin1	0.000236343	0.20382721	6
Hist1h3b	0.000240892	0.282765801	6
Smtn	0.000241468	0.238359219	6
Cald1	0.000247193	0.287744899	6
Ncl	0.000250161	0.29414422	6
Leng8	0.000251987	0.299038036	6
Stard8	0.000262304	0.197390114	6
Necap2	0.00026322	0.235968344	6
Sdsl	0.000268761	0.308224386	6
Elk3	0.000268996	0.285119371	6
Snrnp70	0.000275398	0.319386108	6
Dock1	0.00027939	0.253262979	6
Supt5	0.000281176	0.294550334	6
Tob2	0.000284696	0.326402038	6
Pkm	0.000292433	0.272368016	6
Pum2	0.000295576	0.328902841	6
Kalrn	0.000298483	0.307187344	6
Meis1	0.000312778	0.181528355	6
Unc45a	0.000319965	0.202290423	6
Sult5a1	0.000329677	0.270253239	6
Vezf1	0.000331619	0.221569163	6
Rcc2	0.000371851	0.274780147	6
Plvap	0.000380232	0.397312051	6
Gpi1	0.000397661	0.276127269	6
Dnajc10	0.000398631	0.281291472	6
Nedd9	0.00040698	0.1822838	6
Ppp1r18	0.000435853	0.262390898	6
Ywhaq	0.000445753	0.325248247	6
Ctnnb1	0.000448537	0.268231048	6
Sav1	0.000449018	0.280543126	6
Bhlhe40	0.000454858	0.29807791	6
Pecam1	0.000472891	0.313881064	6
Hist1h1c	0.000477117	0.326281761	6
Tmpo	0.00047726	0.271952025	6
Bptf	0.000478015	0.274814224	6
Tns2	0.000490651	0.315913708	6
Acin1	0.000492373	0.29590268	6
Rp2	0.000499232	0.278031885	6
Rack1	0.000514747	0.249794467	6
Tat	0.000540641	0.232865311	6
Rcor3	0.00055917	0.183816111	6
Lzts2	0.000563303	0.227893759	6
Cyp4v3	0.000572173	0.242712924	6
Prkca	0.000578352	0.184462744	6
Stk24	0.000606502	0.337270935	6
Gja1	0.000607859	0.236664432	6
2310022B05Rik	0.000613122	0.292511953	6
Etv6	0.000616165	0.23323468	6
Peak1	0.000632168	0.191972336	6
Tep1	0.000658275	0.212501987	6
Prkcd	0.000687448	0.232556439	6
Nop53	0.000690837	0.269438436	6
Ppp1r12a	0.000706294	0.288758989	6
Ecscr	0.000742256	0.25837854	6
Msl2	0.000743127	0.258273414	6
Arpc4	0.000774757	0.300793594	6
Akt1	0.000776545	0.307232441	6
Ulk1	0.000797615	0.261798495	6

Mob4	0.000806016	0.279422114	6
Erp29	0.000820543	0.244715629	6
Bmpr2	0.000822699	0.229597029	6
Tap1	0.000860187	0.313324885	6
Cyp17a1	0.00086589	0.211408352	6
Eps15	0.000895597	0.28825193	6
Tmbim1	0.000902247	0.301896766	6
Atpif1	0.00095009	0.297072461	6
4931406P16Rik	0.001000104	0.201659856	6
Grk2	0.001058861	0.306048876	6
Ctla2a	0.00106299	0.245915253	6
Kat7	0.001105056	0.235415623	6
Jdp2	0.001108049	0.204266613	6
Dbp	0.001160186	0.236625385	6
Hspa5	0.001230707	0.241587148	6
Traf7	0.001292443	0.233376638	6
Khdrbs1	0.001332733	0.267647885	6
Nasp	0.001389082	0.224997508	6
Papss1	0.001409955	0.219847867	6
Epc1	0.001416763	0.223882017	6
Phip	0.00142136	0.215650742	6
Dusp3	0.001582174	0.252265196	6
Aif1	0.001596299	0.243826451	6
Tacc1	0.001614014	0.303644963	6
Sik1	0.001620164	0.181681825	6
Hnrnpm	0.001759981	0.279867817	6
Ankrd44	0.001812515	0.204583808	6
Agtpbp1	0.001835396	0.200119142	6
Cd84	0.001857415	0.217139341	6
Hist2h2bb	0.001872622	0.265057914	6
Thoc2	0.001900927	0.26051379	6
Mark2	0.002011718	0.209204208	6
Itpripl1	0.002017383	0.189215426	6
Hnrnpa3	0.002018847	0.28339607	6
Ogt	0.002091571	0.289226242	6
Uox	0.002137465	0.245060472	6
Esam	0.002168821	0.268204477	6
Apbb2	0.002202217	0.209999465	6
Rbms2	0.002220271	0.252228305	6
Cltc	0.002340449	0.249493805	6
Mafk	0.002418545	0.193943905	6
Cpne1	0.002438241	0.186437498	6
Srgn	0.002456704	0.245551919	6
Oasl2	0.002587841	0.249921387	6
Axl	0.002675343	0.314606127	6
H1f0	0.002775216	0.281401785	6
Mrtfa	0.00278276	0.241692758	6
Atp1b3	0.002804129	0.251048219	6
Gnptab	0.002838724	0.19852851	6
Srsf11	0.002845094	0.302096011	6
Sdcbp	0.002868632	0.319243842	6
Ddx17	0.002958934	0.296066799	6
Degs1	0.003052876	0.261905539	6
Rasgrp2	0.003112467	0.256237104	6
Ctcf	0.003178515	0.274780147	6
Kat6a	0.003336729	0.291835311	6
Cobl	0.003347257	0.262022088	6
Ybx3	0.003493062	0.273569601	6
Uhmk1	0.003515096	0.229385072	6

Sike1	0.003552273	0.224743984	6
Hipk1	0.003573048	0.28894992	6
Arg1	0.003679879	0.232913931	6
Nfe2l2	0.003715419	0.241339222	6
Cdk4	0.003746959	0.218286949	6
Snrpe	0.003766255	0.266234581	6
Mbnl2	0.003855501	0.259324004	6
Klc1	0.003945	0.222844008	6
Crtap	0.003959761	0.196179446	6
Tbc1d8	0.004048394	0.222791153	6
Rarres2	0.004146909	0.224726641	6
Foxj3	0.004154696	0.234874188	6
Tgfbr3	0.004171672	0.248307935	6
Lmna	0.004209082	0.275312937	6
Cxcl16	0.004209946	0.220904532	6
Tusc3	0.004278998	0.191710356	6
Ddx6	0.00428033	0.27214117	6
Vcl	0.004616923	0.310428374	6
Bcl7c	0.004627817	0.204113658	6
Rab31	0.004652028	0.23619544	6
Glg1	0.004698765	0.251955712	6
Aplp2	0.004725809	0.260931088	6
Zfp395	0.004746345	0.246196292	6
Notch2	0.004889786	0.283308584	6
Pawr	0.005007847	0.23258223	6
Uroc1	0.00512644	0.257398807	6
Snx14	0.005143951	0.199182528	6
Lnpep	0.005191493	0.258353216	6
Nisch	0.00545402	0.277617968	6
Pelp1	0.005484214	0.170678598	6
Ptbp3	0.005540091	0.273948717	6
Anp32b	0.005552261	0.253481566	6
Ctnnbip1	0.005689244	0.229827932	6
Gm8909	0.00572411	0.257393283	6
Pafah1b3	0.005911777	0.165518581	6
Selenow	0.005952071	0.246561204	6
Clec12a	0.005952261	0.182381413	6
Zfp740	0.006000275	0.217529485	6
H2-K1	0.00613721	0.242145783	6
Kctd12	0.00614192	0.194227576	6
Tjp1	0.006319288	0.263358967	6
Bicd2	0.006375451	0.207238401	6
Resf1	0.00639913	0.205749306	6
Ktn1	0.006592812	0.24159084	6
Gbp3	0.006600454	0.15148056	6
Pdia3	0.006763645	0.226896218	6
F2r	0.006863862	0.239590718	6
C1qa	0.006973182	0.299968479	6
Cyfip1	0.007080922	0.203857309	6
Mapk8ip3	0.007146058	0.225323581	6
Arhgap26	0.007202141	0.226182273	6
Trim28	0.007219749	0.256789999	6
Rap1b	0.007322208	0.285052357	6
Creb3l2	0.007398254	0.245198454	6
Mycbp2	0.007448649	0.277889735	6
Dync1i2	0.007551555	0.287044289	6
Hivep1	0.007596483	0.220679469	6
Srf	0.007609473	0.170875771	6
Nedd4l	0.007712956	0.254801434	6

Fli1	0.008170897	0.238496029	6
Ppp1r12b	0.008197057	0.238650844	6
Plxna2	0.008289666	0.231212842	6
Tinagl1	0.008667811	0.283218117	6
Tmem123	0.008686271	0.27971276	6
Zfp36l2	0.008890035	0.325415825	6
Stard3nl	0.008991404	0.174853933	6
Atp2a2	0.009078571	0.260367819	6
Slc25a47	0.009197977	0.225610014	6
Tacc2	0.009333422	0.19532642	6
Nr1d2	0.009390351	0.231142731	6
Trak2	0.009403211	0.224111225	6
Itgb1	0.009776877	0.302382687	6
Orc6	0.009906998	0.186342182	6
Cd2bp2	0.01052848	0.272384149	6
Mmp15	0.010643022	0.268234439	6
Polr2a	0.010734915	0.252203353	6
Ildr2	0.01108044	0.204006246	6
Ppp2r2a	0.011311638	0.18591595	6
Arhgdib	0.0113497	0.323710971	6
Os9	0.01138097	0.239886475	6
Ighm	0.011656222	0.236426096	6
Klf2	0.011683716	0.307558065	6
Cdc42se2	0.011766069	0.225753957	6
Igf1	0.011778398	0.245087769	6
Fabp4	0.011888561	0.239556502	6
Snrnp200	0.012013882	0.232932709	6
Arpc2	0.012252134	0.244951247	6
Ppp1r12c	0.012474615	0.240915252	6
Hnrnpdl	0.012552742	0.222006064	6
Smad6	0.012782185	0.239524514	6
Man2a1	0.012849643	0.263298179	6
Aqp1	0.013026693	0.211097424	6
Lsm14a	0.013036675	0.256585298	6
Selenop	0.013193011	0.207954917	6
Cd37	0.013207931	0.198851963	6
Anapc11	0.013507918	0.240225589	6
Pgs1	0.013646912	0.161118401	6
Ankrd13a	0.013691278	0.241765089	6
Rbm5	0.013921101	0.306849159	6
Il6st	0.014533348	0.267317151	6
Lurap1l	0.014542325	0.318831275	6
Scaf8	0.014850336	0.196874489	6
Cc2d1a	0.014999539	0.174495973	6
Adarb1	0.015064861	0.194433348	6
Pogz	0.015078974	0.165132452	6
Arhgap18	0.015186988	0.174187166	6
Ubc	0.015218081	0.255027533	6
Mapk3	0.015276566	0.224018156	6
Phf2	0.015685014	0.236267109	6
Myrf	0.015992891	0.27665988	6
Hist1h2bb	0.016507406	0.276872081	6
Zfp68	0.017186631	0.233125737	6
Arhgap45	0.017351567	0.183077563	6
Kidins220	0.017388689	0.252014623	6
Myo9b	0.01763335	0.225821436	6
Cep68	0.017767148	0.168580743	6
Cic	0.017774169	0.197318697	6
Baz2b	0.018606271	0.242648853	6

Pld4	0.018638645	0.289726517	6
Parva	0.018785357	0.261843748	6
Cebpd	0.019222588	0.25824047	6
Trim44	0.019260166	0.271715472	6
Rtn4rl1	0.019584492	0.174606718	6
Fus	0.019756903	0.247815488	6
Lpp	0.019836804	0.271577714	6
Slc25a51	0.019877338	0.225209237	6
Eif3f	0.020446292	0.23211405	6
Spred2	0.020580828	0.185138899	6
Esr1	0.021754048	0.225556031	6
Tpd52	0.022216413	0.160008231	6
Itpr1	0.02236264	0.206972181	6
Chd9	0.022426105	0.176245729	6
Rbm42	0.022521944	0.192317986	6
Etnk1	0.023548012	0.264136101	6
Rere	0.023553748	0.259080992	6
Mob1a	0.023675785	0.270136083	6
C3	0.024143476	0.208434432	6
Ctps2	0.024380512	0.201811272	6
Loxl2	0.02536981	0.189789021	6
Spi1	0.025392989	0.232251751	6
Slc7a2	0.025476358	0.234776351	6
Pkig	0.02613059	0.1608005	6
Cetn2	0.026765438	0.221683331	6
Ppp3cb	0.0272424	0.229419613	6
Plekha1	0.027328412	0.225331721	6
Grn	0.027389692	0.26197469	6
Lgals3bp	0.027450864	0.241035482	6
Parp14	0.028984775	0.217934286	6
Fbh1	0.029644597	0.198437757	6
Eepd1	0.030063352	0.221332968	6
Map7d1	0.030612216	0.278727331	6
Fndc3b	0.030852412	0.245699945	6
Slfn5	0.031124945	0.241165866	6
Hnrnpab	0.031566481	0.282259323	6
Rras	0.031610395	0.234444989	6
St6gal1	0.032404605	0.280676186	6
Tyrobp	0.032424055	0.274141645	6
Smarcb1	0.032529263	0.236191649	6
Carmil1	0.032563571	0.171651018	6
Cab39	0.032800192	0.241551707	6
Cuedc2	0.032911507	0.213521559	6
Cbx7	0.033194193	0.17387599	6
Sqle	0.03343953	0.274519944	6
Prkx	0.033677519	0.176404791	6
Kcnk5	0.034841511	0.169204719	6
Mfsd2a	0.035167161	0.201306962	6
Trio	0.035382685	0.183003096	6
Arhgef40	0.03562712	0.165369944	6
Clca3a1	0.035805161	0.169356594	6
Nckap1l	0.036761653	0.16013135	6
Frg1	0.037035717	0.179652486	6
Dab2	0.03862833	0.26080575	6
Lrrfip1	0.039145939	0.288778627	6
Git1	0.039657404	0.219780374	6
Csgalnact2	0.040322105	0.22286203	6
Phf14	0.040572182	0.222946147	6
Ttc14	0.040760177	0.160932766	6

Tbc1d10a	0.041260794	0.169267315	6
H2afy	0.041690712	0.270098141	6
Srrm2	0.041722541	0.24970117	6
Gna13	0.042944989	0.237105238	6
Fmr1	0.043660676	0.239305345	6
Nectin2	0.045204035	0.238628919	6
Sf3b1	0.045376143	0.293336696	6
Fam49b	0.046243074	0.225825295	6
Naf1	0.046565463	0.174853933	6
Card19	0.047382956	0.184177598	6
Gimap1	0.047860395	0.14991834	6
Il10rb	0.048129847	0.258101405	6
Slco2a1	0.048788575	0.229049058	6
Hnrnpk	0.048984857	0.238341937	6
Stk11	0.049118108	0.231988711	6
Arpin	0.049794169	0.149577112	6
Anapc1	0.049825468	0.243213397	6

Table S2. Differentially expressed genes (DEGs), AEG-1$^{\Delta MAC}$ vs AEG-1$^{fl/fl}$ livers, clusters in ST analysis

	Gene	p_val_adj	avg_log2FC
**Cluster 0**	Alas1	3.29E-276	2.2872267
	Gadd45g	6.99E-284	2.2468132
	Phlda1	1.43E-261	2.1366471
	Onecut1	5.73E-187	1.4794742
	Cish	8.87E-211	1.4627926
	Foxq1	3.85E-191	1.4571857
	Cyp2c54	3.67E-117	1.2160162
	Serpina12	1.22E-118	1.1762268
	Lpin1	1.26E-113	1.1242408
	Dusp1	2.6E-111	1.0947276
	Cebpb	2.89E-131	1.0901127
	Mup12	2.1E-96	1.0159664
	Cyp1a2	2.24E-99	1.0026826
	Rbp1	2.08E-108	-1.168954
	Lpl	1.75E-102	-1.182925
	Scd1	9.96E-152	-1.22696
**Cluster 1**	Alas1	2.18E-136	2.4446433
	Phlda1	2.35E-141	2.2545967
	Gadd45g	5.56E-123	2.1230732
	Errfi1	8.04E-232	2.0800891
	Mup17	2.92E-173	1.8859456
	Onecut1	2.75E-104	1.7666802
	Mup11	4.62E-195	1.6914592
	Mup10	1.06E-174	1.4673254
	Foxq1	1.8E-80	1.4434253
	Cyp2e1	2.36E-136	1.4342409
	Cish	2.68E-102	1.4340702
	Lpin1	5.05E-61	1.2423338
	Dusp1	1.48E-65	1.2379981
	Cyp2c54	6.01E-80	1.2358618
	Hamp	2.81E-69	1.2222701
	Cebpb	7.06E-82	1.1152899
	Arid5b	2.93E-52	1.0966627
	Mup12	8.39E-48	1.024329
	Depp1	3.36E-43	-1.074001
	Fabp5	2.45E-46	-1.150104
	Egr1	5.99E-50	-1.250773
	Lpl	7.05E-74	-1.482268
	Rbp1	5.41E-79	-1.528881
	Scd1	5.84E-92	-1.579411
	Tff3	1.08E-148	-3.066173
**Cluster 2**	Alas1	5.05E-154	2.6252717
	Gadd45g	2.77E-156	2.3511415
	Phlda1	9.54E-145	2.1916089
	Mup11	3.81E-121	2.0325504
	Errfi1	1.37E-192	1.7794336
	Foxq1	4.18E-112	1.7499217
	Onecut1	4.72E-113	1.7112274
	Cish	3.36E-128	1.7063469
	Mup17	5.42E-120	1.6660638
	Cyp2e1	1.17E-50	1.5633386
	Mup10	1.16E-144	1.4905779
	Lpin1	3.22E-78	1.3669351
	Serpina12	2.12E-75	1.3518998
	Dusp1	4.92E-76	1.2944183

	Mup12	1.6E-68	1.2885638
	Arid5b	1.88E-63	1.1565944
	Cyp2c54	1.27E-42	1.079826
	Thrsp	2.98E-54	1.0538951
	Cebpb	3.35E-67	1.0510134
	Hhex	2.32E-58	1.0463354
	Retsat	1.87E-76	1.0381766
	Aacs	6.56E-50	1.0047019
	Fabp5	3.78E-37	-1.021173
	Hbb-bt	4.71E-20	-1.033596
	Egr1	7.21E-45	-1.090216
	Lpl	7.37E-61	-1.218095
	Depp1	2.21E-62	-1.221411
	Rbp1	9.76E-76	-1.41099
	Scd1	8.25E-102	-1.558369
	Tff3	7.49E-161	-3.214271
**Cluster 3**	Mup17	8.89E-155	2.4704162
	Alas1	8.14E-108	2.3505115
	Errfi1	6.16E-150	2.2241751
	Gadd45g	9.5E-106	2.1084741
	Phlda1	2.29E-85	2.1053204
	Mup11	1.11E-151	1.7039299
	Mup10	9.64E-122	1.6525003
	Hamp	5.01E-73	1.6491633
	Cyp2e1	2.61E-142	1.5594293
	Onecut1	2.86E-79	1.4932277
	Cish	2.78E-73	1.4545508
	Foxq1	2.99E-54	1.2978122
	Cyp2c54	1E-53	1.2975486
	Ces3b	2.9E-83	1.188229
	Cd74	2.73E-39	1.1630332
	Mup12	8.15E-41	1.1586038
	Cyp1a2	1.36E-65	1.1463244
	Cebpb	3.98E-49	1.0990518
	Hhex	3.83E-36	1.0544392
	Sult1d1	1.29E-48	1.051771
	Lpin1	3.62E-36	1.03434
	Pck1	3.23E-58	1.0252746
	Dusp1	1.04E-34	1.0072103
	Lpl	3.48E-49	-1.19492
	Scd1	5.42E-91	-1.281305
	Rbp1	4.64E-53	-1.2925
	Tff3	8.44E-89	-2.188889
**Cluster 4**	Mup17	5.58E-205	2.3179509
	Alas1	1.28E-108	2.3026959
	Errfi1	4.63E-157	2.2141407
	Gadd45g	6.41E-108	2.181848
	Phlda1	1.7E-103	2.1287047
	Hamp	3.1E-71	1.6150254
	Mup10	1.56E-138	1.5774149
	Onecut1	8.73E-72	1.5138703
	Cyp2c54	4.53E-72	1.2616921
	Mup11	4.36E-112	1.2451005
	Cish	4.16E-63	1.2403875
	Ces3b	1.14E-90	1.2105215
	Foxq1	9.04E-55	1.1858201
	Lpin1	3.48E-39	1.1238751

	Cd74	3.06E-35	1.1021023
	Cyp2e1	1.44E-114	1.0991153
	Sult1d1	8.11E-59	1.0982775
	Saa4	7.56E-59	1.0784121
	Dusp1	6.67E-40	1.0490042
	Gpcpd1	3.48E-41	1.0423441
	Hhex	3.04E-34	1.0244292
	Pck1	7.57E-56	1.0234463
	Mup12	3.62E-40	1.0042874
	Cyp4a14	8.19E-36	-1.004021
	Rbp1	2.51E-32	-1.062879
	Apoa4	3.95E-11	-1.09928
	Lpl	1.06E-38	-1.139547
	Scd1	4.97E-57	-1.395642
	Tff3	2.54E-97	-2.064665
**Cluster 5**	Alas1	4.25E-100	2.6004439
	Mup17	1.16E-104	2.3849312
	Errfi1	9.76E-130	2.3644474
	Phlda1	1.67E-95	2.2212448
	Gadd45g	4.03E-87	2.1034694
	Onecut1	2.27E-80	1.8819633
	Mup10	7.95E-113	1.7426717
	Hamp	1.26E-32	1.5518675
	Cyp2c54	4.98E-73	1.4381445
	Cish	6.01E-72	1.4251523
	Mup11	1.14E-97	1.3802047
	Lpin1	1.47E-41	1.2789353
	Cyp2e1	2.74E-83	1.1842421
	Ces3b	1.45E-74	1.1606322
	Saa4	1.85E-52	1.1562335
	Mup12	8.17E-38	1.1509575
	Npr2	6.67E-32	1.1473689
	Plk3	6.77E-45	1.1175787
	Dusp1	6.4E-34	1.1171656
	Slc1a2	1.1E-08	1.1116762
	Foxq1	8.8E-35	1.0974433
	Gpcpd1	1.31E-33	1.0946202
	Sult1d1	1.76E-48	1.0871138
	Selenbp2	2.8E-42	1.0639451
	Arid5b	4.4E-32	1.0540529
	Aldh1a1	1.09E-48	1.0493209
	Cebpb	1.11E-43	1.033668
	Hhex	7.76E-36	1.0253675
	Hamp2	1.96E-10	1.0163166
	Pim3	1.68E-32	1.0072178
	Igtp	0.0000886	1.0013131
	Serpina1e	1.32E-45	-1.002705
	Serpina7	4.94E-23	-1.054222
	Depp1	4.75E-33	-1.091411
	Acly	5.18E-41	-1.11441
	Lpl	2.81E-49	-1.227377
	Fabp5	1.35E-42	-1.263506
	Rbp1	5.46E-52	-1.350841
	Scd1	1.02E-86	-1.699537
	Tff3	6.22E-100	-2.326937
**Cluster 6**	Hbb-bs	0.0828124	3.1287509
	Hba-a2	0.0799476	3.1103366

Alas1	7.55E-46	2.4559017
Gadd45g	1.16E-43	2.0833922
Phlda1	2.38E-46	2.0350046
Mup17	1.56E-35	1.7303776
Foxq1	4.16E-28	1.5701636
Mup11	3.62E-25	1.5303443
Errfi1	6.62E-39	1.447989
Serpina12	1.38E-26	1.4407737
Onecut1	3.04E-24	1.3587354
Cyp2e1	9.02E-14	1.3149521
Lpin1	3.55E-17	1.3108528
Hbb-bt	1	1.2580712
Cish	4.73E-26	1.2555715
Mup10	3.66E-23	1.2219768
Thrsp	1.47E-19	1.1737059
Cyp2c54	1.43E-10	1.0147322
Dusp1	3.2E-14	1.0107936
Etnppl	4.58E-17	-1.005038
Marco	6.39E-17	-1.067484
Depp1	1.07E-15	-1.17814
Efna1	4.22E-18	-1.247758
Scd1	5.76E-20	-1.281652
Tff3	6.15E-31	-2.161498

Table S3. Canonical pathways activated or inhibited in AEG-1$^{\Delta MAC}$ vs AEG-1$^{fl/fl}$ livers, cell type clusters in scRNA-seq analysis

**Kupffer Cell-1**	**Canonical Pathways**	**-log(p-value)**	**z-score**	**Molecules**
	Neutrophil degranulation	29.4	-3.371	ADAM8,ARG1,CD63,PSAP
	LPS/IL-1 Mediated Inhibition of RXR Function	23.2	-3.162	ABCG5,ALAS1,ALDH1A1,CYP2E1
	Interferon gamma signaling	5.43	-2.53	VCAM1
	Macrophage Alternative Activation Signaling Pathway	5.42	-2.138	ARG1,CSF1R,LPL
	Neuroinflammation Signaling Pathway	5.17	-3.5	CSF1R,SLC1A2,VCAM1
	MHC class II antigen presentation	5.07	-2.714	CD74
	Sphingolipid metabolism	2.48	-2.449	PSAP
	Hepatic Cholestasis	2.37	-3.162	ABCG5,CYP27A1
	Immunoregulatory interactions, Lymphoid vs non-Lymphoid	2.17	-2.333	VCAM1
	Phagosome Formation	1.62	-3.441	PLD3
	Xenobiotic Metabolism AHR Signaling Pathway	13.2	4.123	ALDH1A1,CYP1A2
	LXR/RXR Activation	11.7	2.887	ABCG5,APOA2,FASN,LPL
	Xenobiotic Metabolism CAR Signaling Pathway	10	4.472	ALDH1A1,CYP1A2
	Phenylalanine and tyrosine metabolism	9.99	2.646	HPD
	Xenobiotic Metabolism PXR Signaling Pathway	9.13	4.359	ALDH1A1
	Phase I - Functionalization of compounds	8.82	2.673	ALDH1A1,CYP1A2,CYP27A1,CYP2E1
	Fatty Acid β-oxidation I	8.5	2.828	SDS
	Superpathway of Citrulline Metabolism	8.48	2.449	ARG1,ASS1,GLS2,OAT
	Peroxisomal protein import	8.19	3.317	ACOX1
	Ethanol Degradation II	7.07	2.646	ALDH1A1
	PXR/RXR Activation	6.95	2.53	ALAS1,ALDH1A1,CYP1A2
	Noradrenaline and Adrenaline Degradation	6.87	2.646	ALDH1A1
	Bile acid and bile salt metabolism	6.21	2.646	CYP27A1
	Estrogen Biosynthesis	6.09	2.121	CYP1A2,CYP2E1,HSD17B13,POR
	Peroxisomal lipid metabolism	5.92	2.449	ACOX1
	Nicotine Degradation II	5.74	2.333	CYP1A2,CYP2E1,POR
	Ethanol Degradation IV	5.56	2.236	ALDH1A1
	DHCR24 Signaling Pathway	5.5	3.464	APOA2,VCAM1
	Bile Acid Biosynthesis, Neutral Pathway	5.34	2.236	CYP27A1
	Valine Degradation I	4.81	2.236	SDS
	Serotonin Degradation	4.55	2.646	ALDH1A1
	Arachidonic acid metabolism	3.06	2.236	CYP1A2

	NR1H2 and NR1H3-mediated signaling	2.85	2.236	ABCG5,FASN
	Selenoamino acid metabolism	2.05	2.449	GNMT
**Stellate Cells**	LPS/IL-1 Mediated Inhibition of RXR Function	20.9	-2.646	ABCG5,ALAS1,ALDH1A1,CYP2E1
	Extracellular matrix organization	12.4	-3.051	COL1A1,COL1A2,COL3A1,COL4A2,C
	Collagen degradation	8.04	-2.828	COL1A1,COL1A2,COL3A1,COL4A2,C
	Collagen biosynthesis and modifying enzymes	7.71	-2.828	COL1A1,COL1A2,COL3A1,COL4A2,C
	Collagen chain trimerization	7.71	-2.646	COL1A1,COL1A2,COL3A1,COL4A2,C
	Apelin Liver Signaling Pathway	4.48	-2	COL1A1,COL1A2,COL3A1
	IL-12 Signaling and Production in Macrophages	3.64	-2.828	APOA2,COL1A1,COL1A2,COL3A1
	Signaling by MET	2.67	-2	COL1A1,COL1A2,COL3A1,LAMB1
	IL-17A Signaling in Fibroblasts	2.51	-2	COL1A1,COL1A2,COL3A1,VCAM1
	Hepatic Fibrosis Signaling Pathway	2.04	-2.121	COL1A1,COL1A2,COL3A1, VCAM1
	Hepatic Cholestasis	1.72	-2.236	ABCG5
	IL-4 Signaling	1.33	-2.828	COL1A1,COL1A2,COL3A1,COL4A2,C
	LXR/RXR Activation	10.3	2.828	ABCG5,APOA2,FASN
	Phenylalanine and tyrosine metabolism	8.19	2.236	HPD
	Xenobiotic Metabolism AHR Signaling Pathway	8.05	3	ALDH1A1,CYP1A2
	Xenobiotic Metabolism CAR Signaling Pathway	7.98	3.464	ALDH1A1,CYP1A2
	Tyrosine Degradation I	7.97	2	HPD
	Xenobiotic Metabolism PXR Signaling Pathway	6.95	3.317	ALDH1A1
	Oxidative Ethanol Degradation III	6.95	2	ALDH1A1,CYP2E1
	DHCR24 Signaling Pathway	6.33	3	APOA2,VCAM1
	Phase I - Functionalization of compounds	6.28	2.121	ALDH1A1,CYP1A2,CYP2E1,POR
	Phenylalanine Degradation IV (Mammalian, via Side Chain)	5.69	2	HPD
	Superpathway of Citrulline Metabolism	5.44	2	ASS1,OAT
	Histamine Degradation	4.93	2	ALDH1A1
	Fatty Acid α-oxidation	4.93	2	ALDH1A1
	Putrescine Degradation III	4.85	2	ALDH1A1
	NR1H2 and NR1H3-mediated signaling	4.62	2.236	ABCG5,FASN
	Tryptophan Degradation X (Mammalian, via Tryptamine)	4.48	2	ALDH1A1
	Gluconeogenesis I	4.48	2	FBP1
	Dopamine Degradation	4.18	2	ALDH1A1
	Ethanol Degradation II	4.02	2	ALDH1A1,

	Selenoamino acid metabolism	4.01	2.449	GNMT
	Noradrenaline and Adrenaline Degradation	3.93	2	ALDH1A1
	Stearate Biosynthesis I (Animals)	3.89	2	CYP2E1,FASN !
	Activation of gene expression by SREBF (SREBP)	3.75	2	FASN
	Glucose metabolism	3.64	2.236	FBP1
	Serotonin Degradation	2.79	2	ALDH1A1
	Plasma lipoprotein assembly, remodeling, and clearance	2.73	2	APOA2
**Endothelial cells**	LPS/IL-1 Mediated Inhibition of RXR Function	25.2	-3.162	ABCG5,ALAS1,ALDH1A1,CYP2E1,FA
	Hepatic Cholestasis	2.26	-3	ABCG5,CYP27A1
	BEX2 Signaling Pathway	1.5	-2	SPP1
	LXR/RXR Activation	11.5	3.464	ABCG5,APOA2,FASN,
	Xenobiotic Metabolism PXR Signaling Pathway	11	3.578	ALDH1A1
	Superpathway of Citrulline Metabolism	10.7	2.646	ARG1,ASS1,OAT
	Urea Cycle	10.2	2.236	ARG1,ASS1
	Xenobiotic Metabolism CAR Signaling Pathway	10.1	2.982	ALDH1A1,CYP1A2
	Phase I - Functionalization of compounds	9.57	3.207	ALDH1A1,CYP1A2,CYP27A1,CYP2E1
	Regulation of IGF transport and uptake by IGFBPs	9.42	3.357	APOA2,SPP1
	PXR/RXR Activation	8.64	2.714	ALAS1,ALDH1A1,CYP1A2
	Phenylalanine and tyrosine metabolism	8.41	2.449	HPD
	Xenobiotic Metabolism AHR Signaling Pathway	8.31	3.464	ALDH1A1,CYP1A2
	Oxidative Ethanol Degradation III	8.14	2	ALDH1A1,CYP2E1
	FXR/RXR Activation	7.79	2	ABCG5,CYP27A1,FASN,FBP1
	Nicotine Degradation II	7.3	2.53	CYP1A2,CYP2E1,POR
	Post-translational protein phosphorylation	7.28	2.887	APOA2,SPP1
	Bile Acid Biosynthesis, Neutral Pathway	7.2	2.449	CYP27A1
	DHCR24 Signaling Pathway	6.96	3.606	APOA2
	Citrulline Biosynthesis	6.83	2	ARG1,OAT
	Nicotine Degradation III	6.7	2.121	CYP1A2,CYP2E1,POR
	Tyrosine Degradation I	6.67	2	HPD
	Bile acid and bile salt metabolism	6.6	2.646	CYP27A1
	Arginine Biosynthesis IV	6.19	2	ASS1,OAT
	Urea cycle	5.28	2	ARG1,ASS1
	NR1H2 and NR1H3-mediated signaling	5.13	2.646	ABCG5,FASN

	Ethanol Degradation IV	4.58	2.236	ALDH1A1
	Selenoamino acid metabolism	4.57	3	GNMT
	Phenylalanine Degradation IV (Mammalian, via Side Chain)	4.41	2	HPD
	Glyoxylate metabolism and glycine degradation	4.33	2.236	GNMT
	Autism Signaling Pathway	4.3	2.324	ALDH1A1,CYP27A1
	Phase II - Conjugation of compounds	4.28	2.646	CYP1A2
	Stearate Biosynthesis I (Animals)	4.11	2.449	CYP2E1,FASN
	Ethanol Degradation II	3.85	2.236	ALDH1A1
	Noradrenaline and Adrenaline Degradation	3.73	2.236	ALDH1A1
	Histamine Degradation	3.67	2	ALDH1A1
	Fatty Acid α-oxidation	3.67	2	ALDH1A1
	Putrescine Degradation III	3.58	2	ALDH1A1
	Tryptophan Degradation X (Mammalian, via Tryptamine)	3.23	2	ALDH1A1
	Dopamine Degradation	2.94	2	ALDH1A1
	Arachidonic acid metabolism	2.39	2	CYP1A2
	Serotonin Degradation	2.38	2.236	ALDH1A1
	Plasma lipoprotein assembly, remodeling, and clearance	2.3	2.236	APOA2
	Glucose metabolism	1.53	2	FBP1
**Pericentral hepatocytes-1**	Response to elevated platelet cytosolic Ca2+	11.9	-2.236	CD63,PSAP
	LXR/RXR Activation	11.5	-2	ABCG5, APOA4,CYP7A1,
	Neutrophil degranulation	10.1	-3.773	ARG1, CD63,LCN2, PSAP
	Plasma lipoprotein assembly, remodeling, and clearance	6.63	-2.111	APOA4
	Regulation of lipid metabolism by PPARalpha	3.96	-2.53	CYP7A1
	Interferon gamma signaling	2.63	-2.646	VCAM1
	Cell surface interactions at the vascular wall	2.55	-2.111	CD74
	Pulmonary Fibrosis Idiopathic Signaling Pathway	1.94	-2.309	EGFR
	Immunoregulatory interactions, Lymphoid vs non-Lymphoid	1.76	-3	VCAM1
	Iron uptake and transport	1.59	-2	LCN2
	Leukocyte Extravasation Signaling	1.46	-2.646	VCAM1
	Hepatic Fibrosis Signaling Pathway	1.43	-2.887	SPP1, VCAM1
	Superpathway of Citrulline Metabolism	11.4	2.828	ARG1,ASS1,GLS2,OAT
	Citrulline Biosynthesis	9.95	2.449	ARG1,GLS2,OAT
	Phenylalanine and tyrosine metabolism	7.66	2.449	HPD,

	Urea Cycle	7.27	2	ARG1
	Nicotine Degradation III	6.67	2.333	CYP1A2, CYP2E1, POR
	PXR/RXR Activation	6.33	2.53	CYP1A2, CYP7A1
	Tyrosine Degradation I	6.17	2	HPD
	Xenobiotic Metabolism CAR Signaling Pathway	5.88	2	CYP1A2,EGFR,Sult1d1
	Urea cycle	4.79	2	ARG1
	Melatonin Degradation I	4.5	2.121	CYP1A2, CYP2E1,POR,Sult1d1
	Phase II - Conjugation of compounds	4.35	2.121	CYP1A2
	Superpathway of Melatonin Degradation	4.26	2.121	CYP1A2, CYP2E1, POR,Sult1d1
	Glyoxylate metabolism and glycine degradation	3.74	2.236	GNMT
**Pericentral hepatocytes-2**	Response to elevated platelet cytosolic Ca2+	7.49	-2.714	CD63,PSAP
	Neutrophil degranulation	5.58	-2	ARG1,CD63,FABP5,LCN2,PSAP
	Degradation of the extracellular matrix	3.22	-2.236	SPP1
	Iron uptake and transport	2.44	-2	LCN2
	TR/RXR Activation	2.23	-2.236	THRSP
	Sphingolipid metabolism	1.34	-2	PSAP
	NRF2-mediated Oxidative Stress Response	15	2.236	Cyp1a2, Cyp2c29, CYP2E1
	Superpathway of Citrulline Metabolism	8.92	2.121	ARG1,ASS1,GLS2, OAT
	FXR/RXR Activation	7.73	2.111	ABCG5,TXNIP
	Xenobiotic Metabolism CAR Signaling Pathway	7.1	2.333	Cyp1a2,EGFR
	Arginine Degradation I (Arginase Pathway)	5.37	2	ARG1 ,OAT
	Tyrosine Degradation I	5.37	2	HPD
	Urea Cycle	4.61	2	ARG1,ASS1,
	NR1H2 and NR1H3-mediated signaling	4	2.236	ABCG5,PCK1
	Urea cycle	3.98	2	ARG1,ASS1
**Periportal hepatocytes-2**	Plasma lipoprotein assembly, remodeling, and clearance	4.97	-2.646	APOA4
	Neutrophil degranulation	4.43	-2.138	ACLY, CD63, LCN2, PSAP
	Cyclophilin Signaling Pathway	3.67	-2.333	SPP1
	Retinoid metabolism and transport	2.6	-2	APOA4
	Sphingolipid metabolism	1.37	-2	PSAP
	FXR/RXR Activation	4.33	2.828	CYP7A1,FASN,TXNIP
	Hematoma Resolution Signaling Pathway	1.64	2.236	DUSP1

**Hepatocytes**	Neutrophil degranulation	14.2	-3.395	ACLY,ARG1,CD63,LCN2
	LXR/RXR Activation	8.06	-2.524	ABCG5,APOA4,CYP7A1,SAA1
	Degradation of the extracellular matrix	4.7	-2.496	SPP1
	Tumor Microenvironment Pathway	4.03	-2.837	ARG1,SPP1
	Ferroptosis Signaling Pathway	4.02	-2.668	GLS2
	Advanced glycosylation endproduct receptor signaling	3.54	-2.236	SAA1
	IL-17A Signaling in Fibroblasts	2.39	-2.111	LCN2,VCAM1
	Plasma lipoprotein assembly, remodeling, and clearance	2.35	-2.53	APOA4
	Hepatic Fibrosis Signaling Pathway	2.33	-2.959	SPP1,VCAM1
	Cargo recognition for clathrin-mediated endocytosis	2.14	-2.309	EGFR
	Iron uptake and transport	2.1	-2.121	LCN2
	Glycolysis I	2	-2.236	PKLR
	Estrogen Receptor Signaling	1.83	-2.117	EGFR
	STAT3 Pathway	1.41	-2.111	EGFR
	ID1 Signaling Pathway	1.34	-2.5	EGFR
	Citrulline Biosynthesis	7.09	2.449	ARG1,GLS2,OAT
	Superpathway of Citrulline Metabolism	6.46	2.646	ARG1,ASS1,GLS2,OAT
	Xenobiotic Metabolism CAR Signaling Pathway	6.34	2.646	CYP1A2,EGFR
	Xenobiotic Metabolism AHR Signaling Pathway	5.91	2.183	CYP1A2
	Glyoxylate metabolism and glycine degradation	5.25	2.333	GNMT
	Phenylalanine and tyrosine metabolism	5.24	2.449	HPD
	Tyrosine Degradation I	4.52	2	HPD
	Phase II - Conjugation of compounds	3.96	2.887	CYP1A2
	Phosphatidylethanolamine Biosynthesis II	3.19	2	CHKA
	Histidine Degradation VI	2.31	2	CYP2E1
	Selenoamino acid metabolism	1.38	2.53	GNMT

:OL6A1,COL6A2,COL6A3,LUM
:OL6A1,COL6A2,COL6A3,MMP14
:OL6A1,COL6A2,COL6A3
:OL6A1,COL6A2,COL6A3

:OL6A1,COL6A2,COL6A3,TIMP3

Table S4. log2 fold change (log2FC) of genes in stellate cells and hepatocytes determined by scRNA-seq of HF/HSD-fed AEG-$1^{fl/fl}$ vs AEG-$1^{\Delta MAC}$ livers.

**Type of cells**	**Gene**	**log2FC AEG-$1^{fl/fl}$ vs AEG-$1^{\Delta MAC}$**
Stellate cells	Col1a1	0.96482
	Col3a1	0.88505
	Col4a2	0.62857
	Col6a2	0.93373
	Mmp14	1.07759
	Lum	1.41367
Hepatocytes	Egfr	1.75439
	Lcn2	1.2065
	Gnmt	-1.086662
	Hpd	-0.815882
